# Structural Diversity and Biological Activities of the Cyclodipeptides from Fungi

**DOI:** 10.3390/molecules22122026

**Published:** 2017-11-23

**Authors:** Xiaohan Wang, Yuying Li, Xuping Zhang, Daowan Lai, Ligang Zhou

**Affiliations:** Department of Plant Pathology, College of Plant Protection, China Agricultural University, Beijing 100193, China; wangxiaohan99@126.com (X.W.); yylimail@163.com (Y.L.); zhangxuping5@163.com (X.Z.); dwlai@cau.edu.cn (D.L.)

**Keywords:** cyclic dipeptides, 2,5-diketopiperazines, epipolythiodioxopiperazines, fungi, biological activities, occurrence, applications

## Abstract

Cyclodipeptides, called 2,5-diketopiperazines (2,5-DKPs), are obtained by the condensation of two amino acids. Fungi have been considered to be a rich source of novel and bioactive cyclodipeptides. This review highlights the occurrence, structures and biological activities of the fungal cyclodipeptides with the literature covered up to July 2017. A total of 635 fungal cyclodipeptides belonging to the groups of tryptophan-proline, tryptophan-tryptophan, tryptophan–Xaa, proline–Xaa, non-tryptophan–non-proline, and thio-analogs have been discussed and reviewed. They were mainly isolated from the genera of *Aspergillus* and *Penicillium*. More and more cyclodipeptides have been isolated from marine-derived and plant endophytic fungi. Some of them were screened to have cytotoxic, phytotoxic, antimicrobial, insecticidal, vasodilator, radical scavenging, antioxidant, brine shrimp lethal, antiviral, nematicidal, antituberculosis, and enzyme-inhibitory activities to show their potential applications in agriculture, medicinal, and food industry.

## 1. Introduction

Cyclodipeptides (or cyclic dipeptides) are usually called 2,5-diketopiperazines (2,5-DKPs) or dioxopiperazines, and result from the condensation of two amino acids such as tryptophan, proline, alanine, histidine, leucine, isoleucine, phenylalanine, serine, and tyrosine [1]. They are the smallest cyclopeptides, and are distributed in many organisms including fungi, bacteria, plants and animals [1,2,3]. Since the first report in 1924, a large number of bioactive cyclodipeptides has been discovered to show cytotoxic, antitumor, antiviral, antifungal, antibacterial, antiprion, antioxidant, antihyperglycemic as well as biofilm and glycosidase inhibitory activities [2,3,4]. Some cyclodipeptides (e.g., tryprostatin A (**103**), tryprostatin B (**104**), FR106969 (**590**), and phenylahistin (**392**)) showed their potential applications [1].

Among the organisms, fungi have been considered as the most important sources of novel and bioactive cyclodipeptides. More and more cyclodipeptides with interesting biological activities have been isolated and characterized from fungi. However, no detailed and comprehensive summary of the fungal cyclodipeptides on their occurrence, structures and biological activities has been reported though chemistry and biology of the cyclodipeptides from either all organisms or a certain class of cyclodipeptides have been documented [3,5,6]. In this review, we aim to describe the diversity of chemical structures and biological activities of the fungal cyclodipeptides and their analogs. A total of 635 fungal cyclodipeptides have been discussed and reviewed with literature covered up to July 2017. According to their biosynthetic origins and structural characters, these cyclodipeptides are classified as tryptophan–proline, tryptophan–tryptophan, tryptophan–Xaa (Xaa is indicated as an unspecified amino acid), proline–Xaa, non-tryptophan–non-proline, and thio analogs. Some special cyclodipeptides (e.g., gunnilactams A–C (**378**–**380**)), which did not belong to 2,5-DKPs, were also included in the group of non-tryptophan-non-proline analogs.

## 2. Tryptophan–Proline Cyclodipeptides

**Figure 1:**
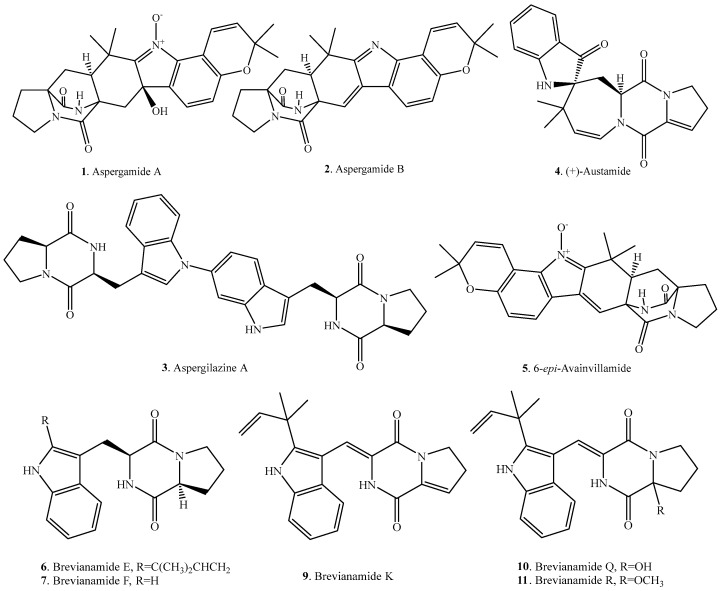

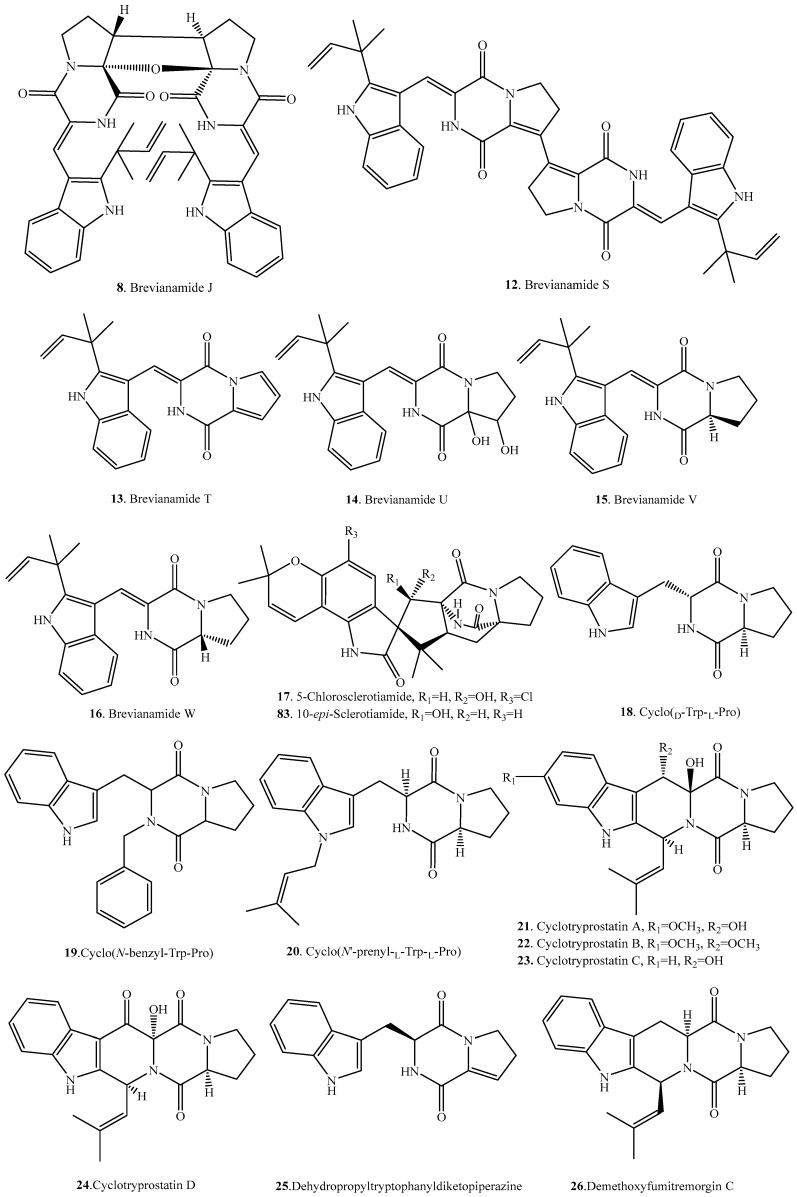

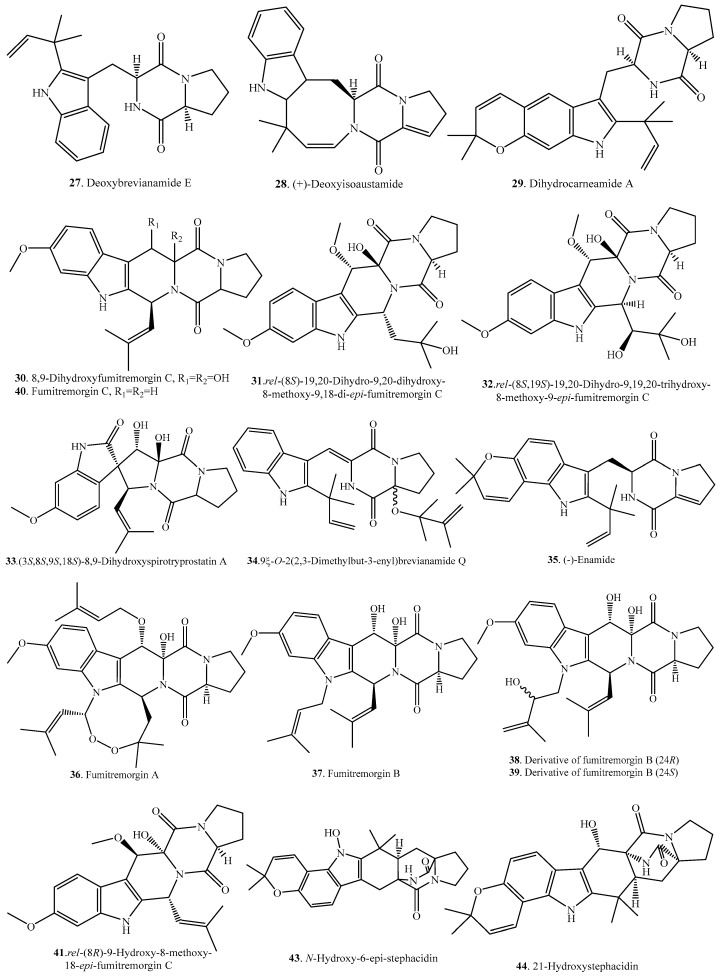

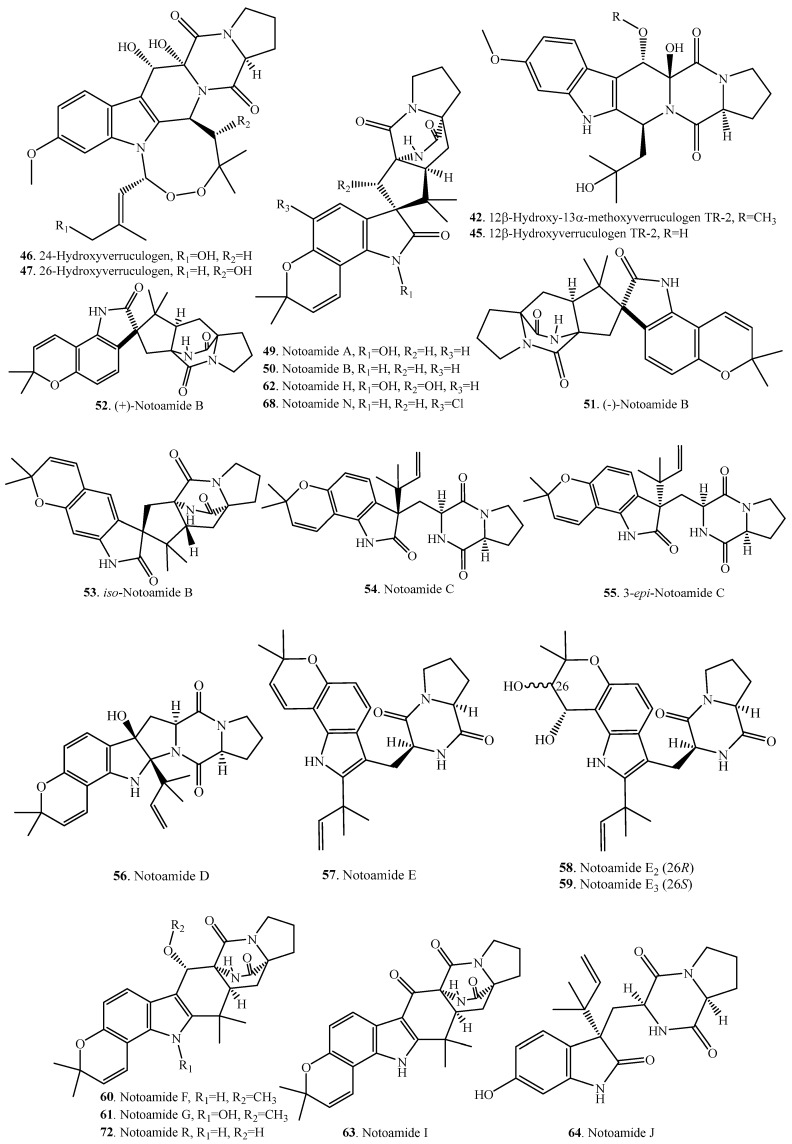

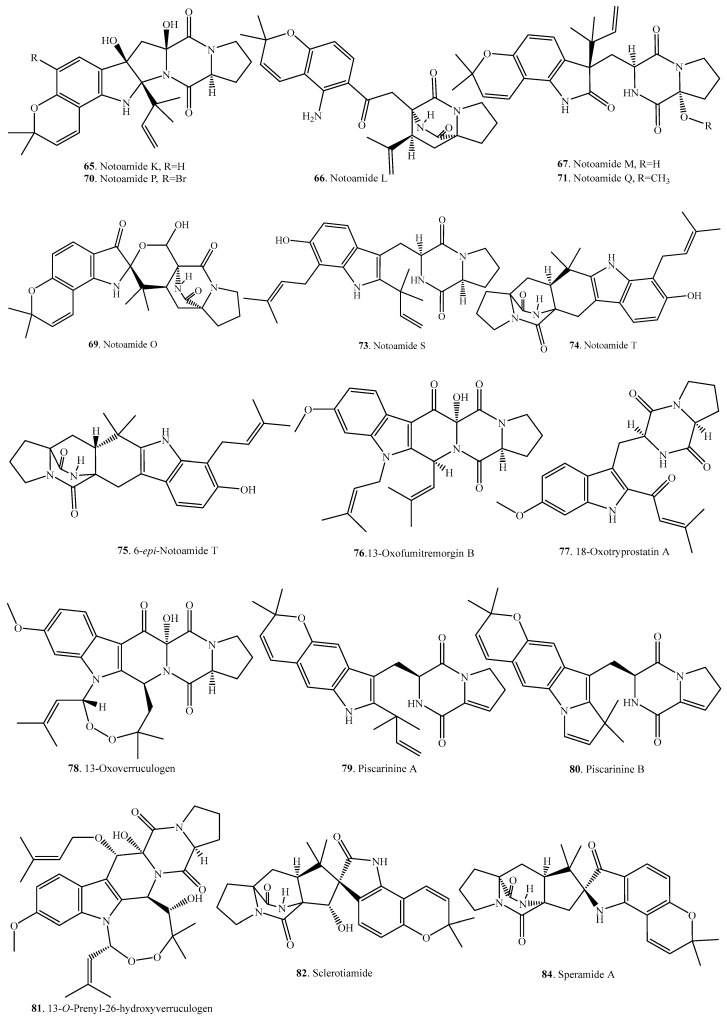

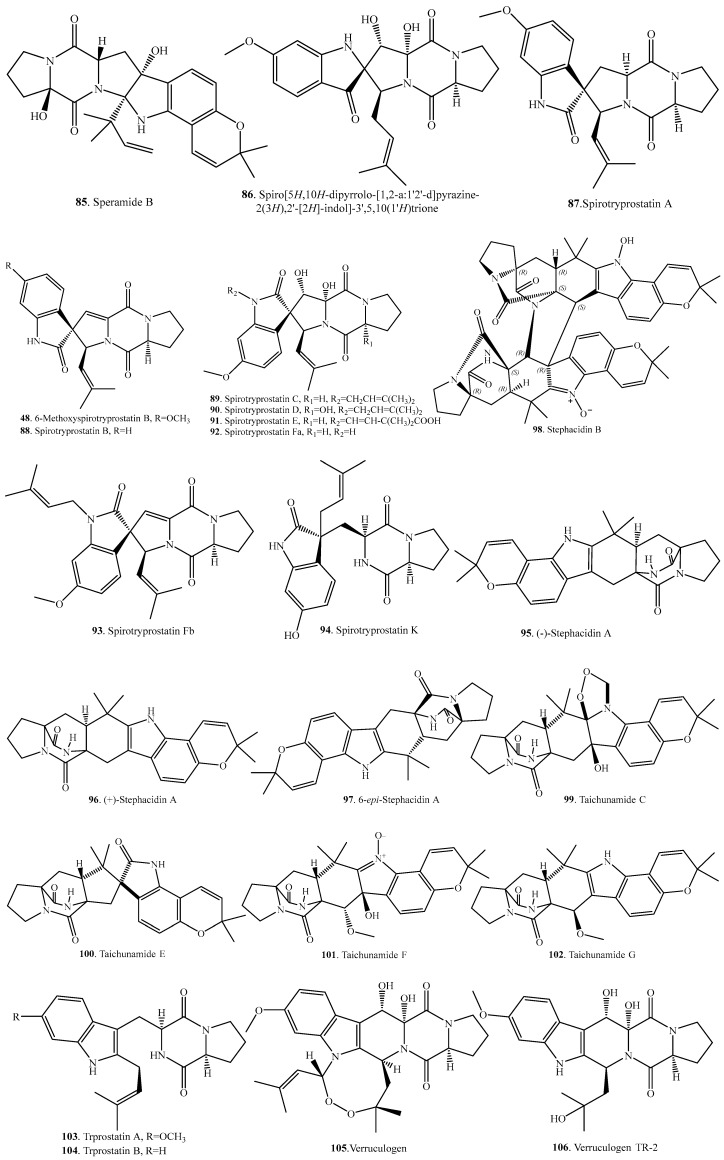

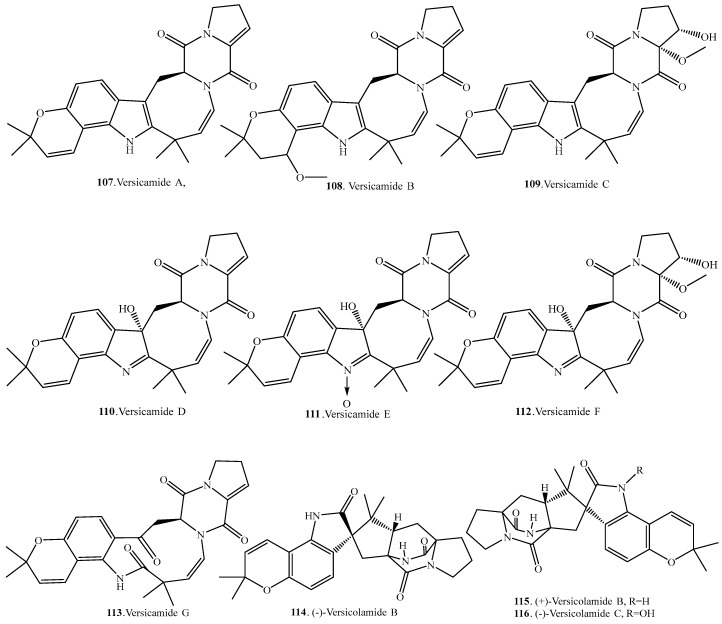
Structures of the tryptophan-proline cyclodipeptide analogs isolated from fungi.

Tryptophan and proline are simultaneously incorporated into the cyclodipeptides in fungi. The proline residue adopts a *cis*-conformation about the Xaa–Pro tertiary amide bond and hence makes the Xaa–Pro sequence prone to cyclodipeptide formation. The tryptophan-proline cyclo(l-Trp–l-Pro) core was derived from condensation of tryptophan and proline residues, and this was often further modified by heterocyclization and isoprenyl addition [6]. The occurrence and biological activities of the tryptophan-proline cyclodipeptides from fungi are listed in Table 1, and their structures are provided in Figure 1.

About 116 tryptophan-proline cyclodipeptides have been isolated from fungi so far. They are mainly distributed in the genera *Aspergillus* and *Penicillium*, and are also distributed in other genera such as *Alternaria*, *Paecilomyces*, and *Pseudallescheria*.

(+)-Austamide (**4**) and (+)-deoxyisoaustamide (**28**) were isolated from the maize meal cultures of the toxigenic fungus *Aspergillus ustus*, and (+)-austamide (**4**) caused acute toxicosis in day-old ducklings [7].

Fumitremorgin C (**40**) and its derivatives (**38**, **39**) were identified in *Aspergillus fumigatus* from the holothurian *Stichopus alternata*. They displayed significant cytotoxic activity against MOLT-4 (human acute lymphoblastic leukemia cells), A-549 (human lung adenocarcinoma epithelial cells), and HL-60 (human promyelocytic leukemia cells), which speculated that this cytotoxic activity may be linked to hydroxyl groups in the side chains of the molecules [8]. Demethoxyfumitremorgin C (**26**) from marine-derived *Aspergillus fumigatus* showed inhibitory activity in the mouse cell cycle against tsFT210, and also inhibited tumor cell cycle arrest at G2/M with a minimum inhibitory concentration (MIC) value of 0.45 μM [9].

18-Oxotryprostatin A (**77**) was isolated from the marine-derived fungus *Aspergillus sydowi* and found to exhibit weak cytotoxic activity against A-549 cells with a median inhibitory concentration (IC_50_) value of 1.28 μM [10]. This compound was also obtained from the endophytic fungus *Aspergillus fumigatus* from *Melia azedarach* to display plant growth inhibitory activity [11].

Spirotryprostatins (**87**–**94**) were isolated from *Aspergillus fumigatus*. These compounds showed cytotoxic activity by inhibiting mammalian cell cycle at G2/M phase [8,12,13].

Stephacidin B (**98**) was isolated from *Aspergillus ochraceus*. This compound exhibited potent cytotoxic activity against LNCaP (a testosterone-dependent prostate cancer cell line), with IC_50_ values from 91 to 621 nM [14].

Both tryprostatins A (**103**) and B (**104**), which are prenylated, were isolated from the fermentation broth of the marine-derived fungus *Aspergillus fumigatus* [15]. Tryprostatin A (**103**) was an inhibitor of the multidrug-resistance breast cancer protein (BCRP) that mediated resistance to chemotherapeutics in breast cancer treatment [16], whereas tryprostatin B (**104**) was a mammalian cell-cycle inhibitor, attractive as a potential anticancer agent [17]. Furthermore, tryprostatin A (**103**) exhibited inhibitory activity on the elongation of lettuce shoots [11].

## 3. Tryptophan–Tryptophan Cyclodipeptides

**Figure 2:**
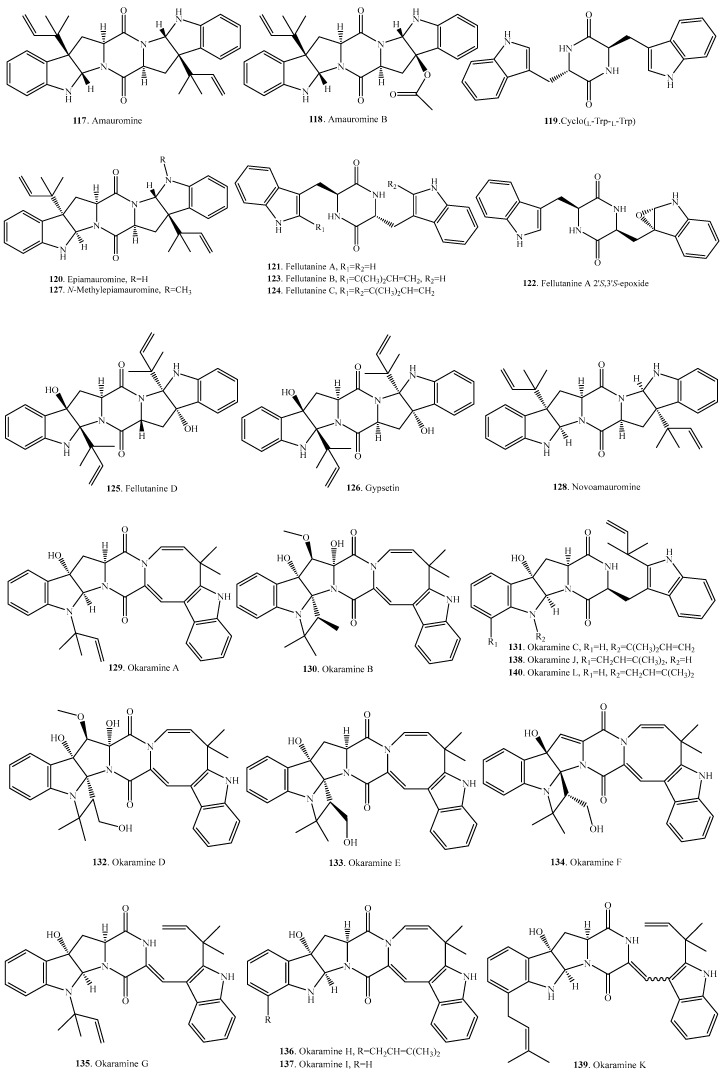

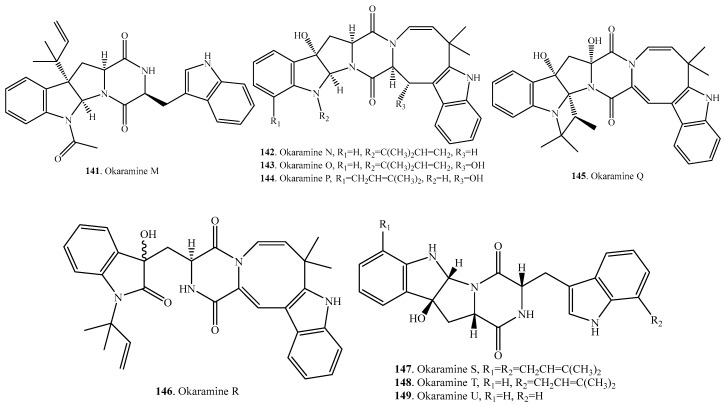
Structures of the tryptophan-tryptophan cyclodipeptide analogs isolated from fungi.

The ditryptophan cyclodipeptides, which have two tryptophan units, are widely distributed in filamentous fungi, especially in the genera *Penicillium* and *Aspergillus*. Their occurrence and biological activities are listed in Table 2, and the structures are provided in Figure 2.

Amauromine (**117**) from *Amauroascus* sp. [54] was identical with nigrifortine (**117**) from *Penicillium nigricans*. It is a diannulated DKP analog which shows hypotensive vasodilating activity [55]. This compound was later isolated from *Auxarthron reticulatum*, and identified as a selective cannabinoid CB1 receptor antagonist [56].

Epiamauromine (**120**) and *N*-methylepiamauromine (**127**) were isolated from the sclerotia of *Aspergillus ochraceus*. They caused moderate reduction in weight gain against the corn earworm *Helicoverpa zea* [57].

Fellutanines A–D (**121**, **123**–**125**), the analogs of cyclo(l-Trp–d-Trp), were isolated from the cultures of *Penicillium fellutanum*. Among them, only fellutanine D (**125**) was diannulated and displayed cytotoxic activity against K-562 (human myeloid leukemia cells), L-929 (mouse fibroblastic cell), and HeLa (human epitheloid cervix carcinoma cells) with IC_50_ values of 9.5, 11.6 and 19.7 μg/mL, respectively [58].

Novoamauromine (**128**) was obtained from *Aspergillus novofumitatus* CBS117520. This compound had inhibitory activity on the cell proliferation of A549, HeLa, LNCap (human prostate carcinoma cells) [59].

Okaramines A–U (**129**–**149**) have been isolated from *Aspergillus aculeatus* [60], *Aspergillus taichungensis* [61], and *Penicillium simplicissimum* [62,63,64,65]. Structure–activity studies indicated the importance of the azetidine and azocine rings to okaramine insecticidal activity [66]. The action of okaramine B (**130**) on silkworm larval neurons using patch-clamp electrophysiology revealed that this compound activated the l-glutamate-gated chloride channel (GluCl) [67].

## 4. Tryptophan–Xaa Cyclodipeptides

**Figure 3:**
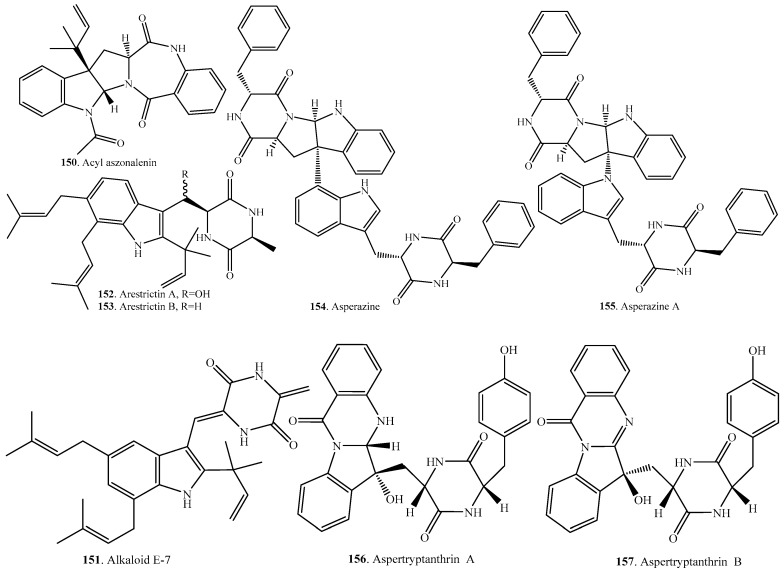

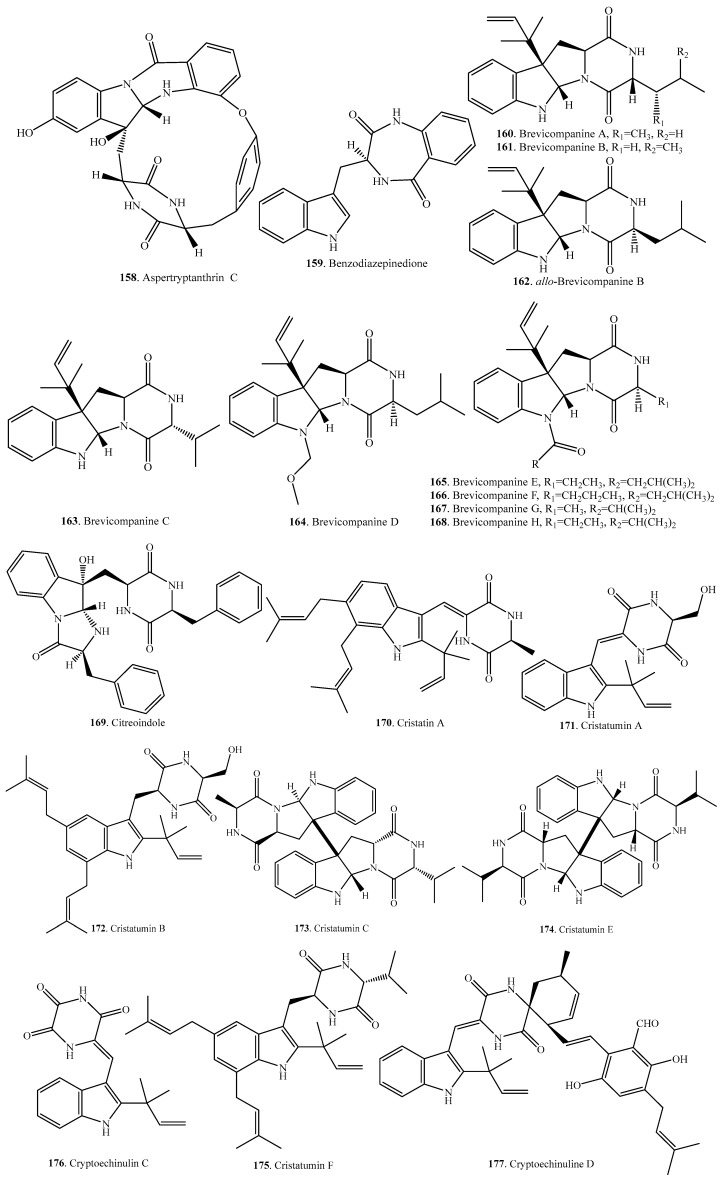

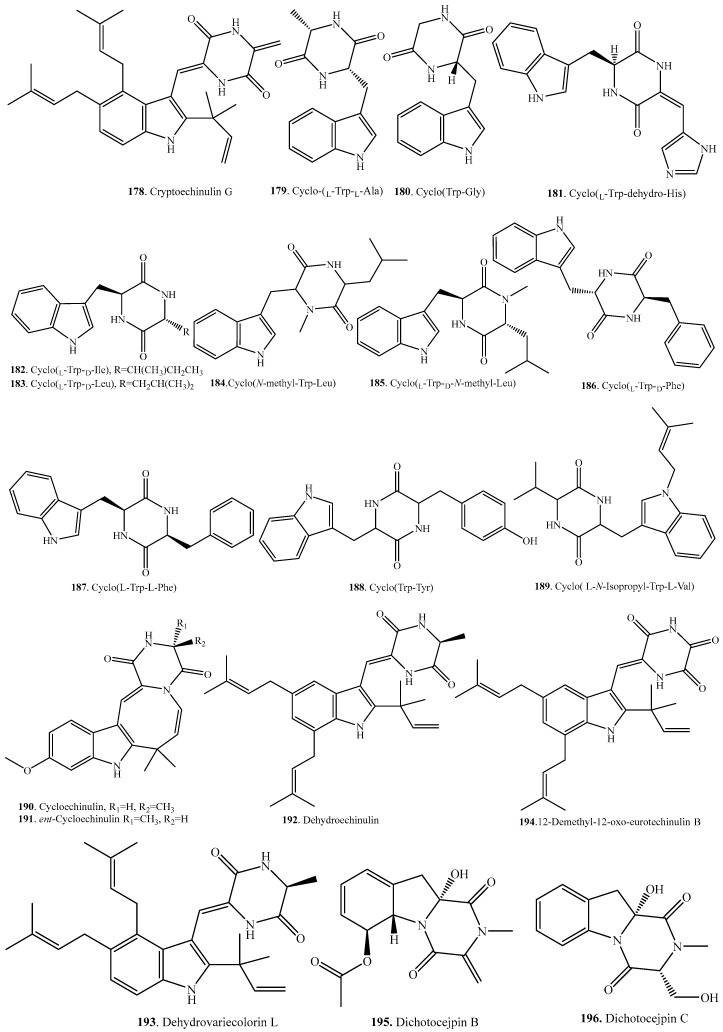

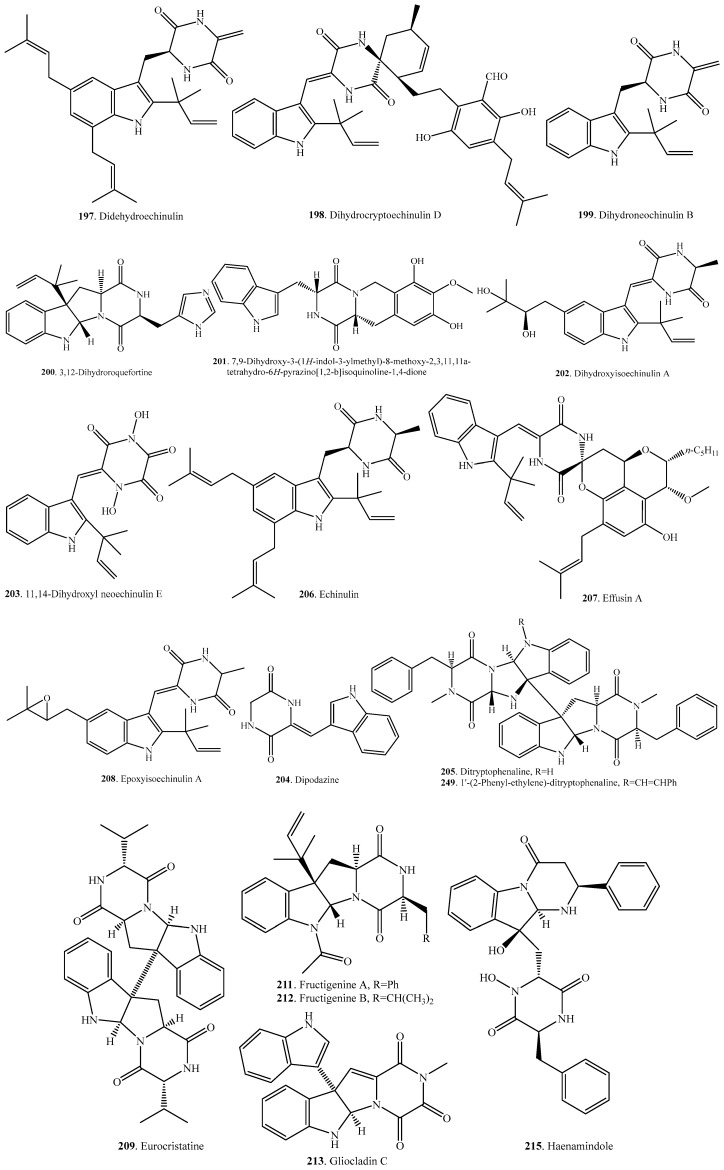

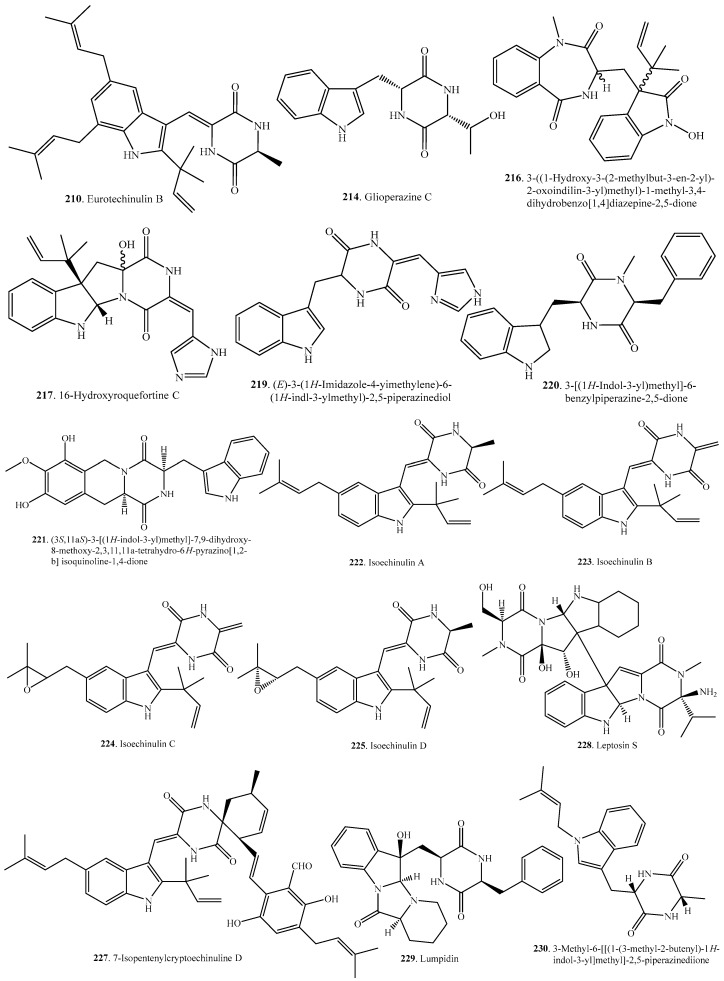

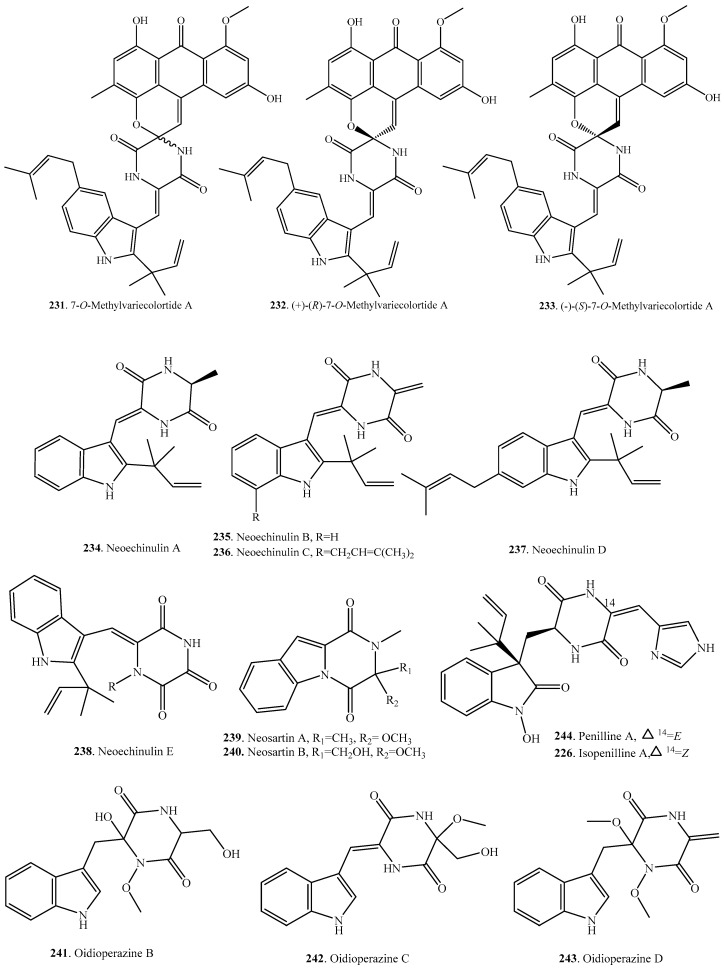

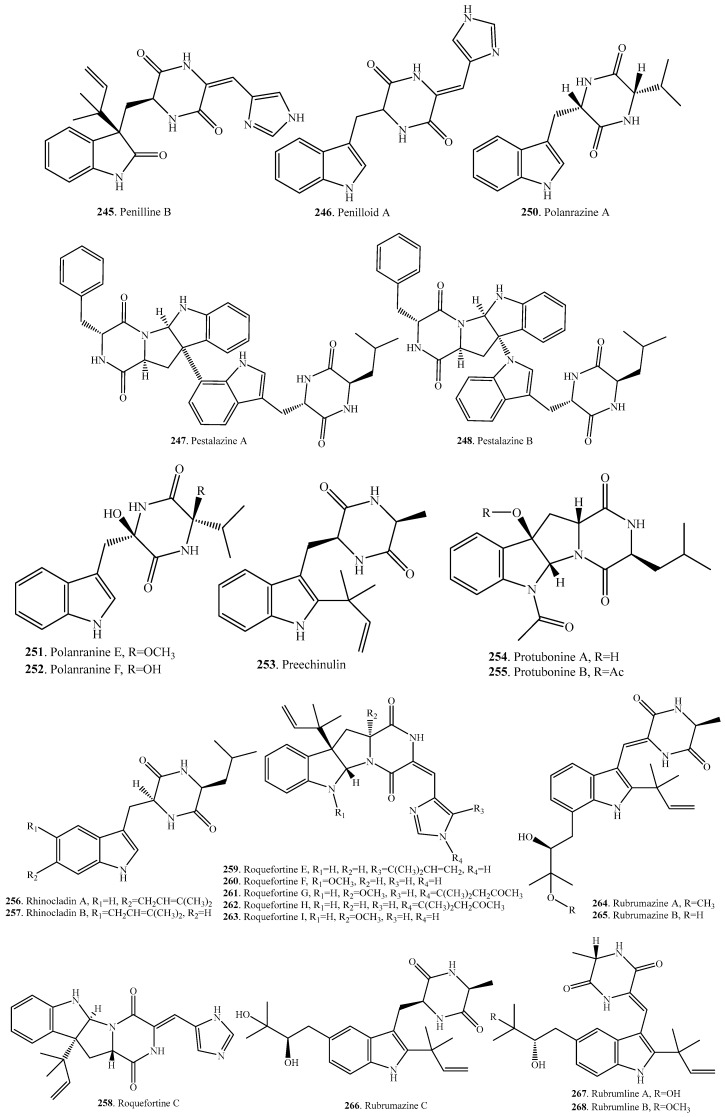

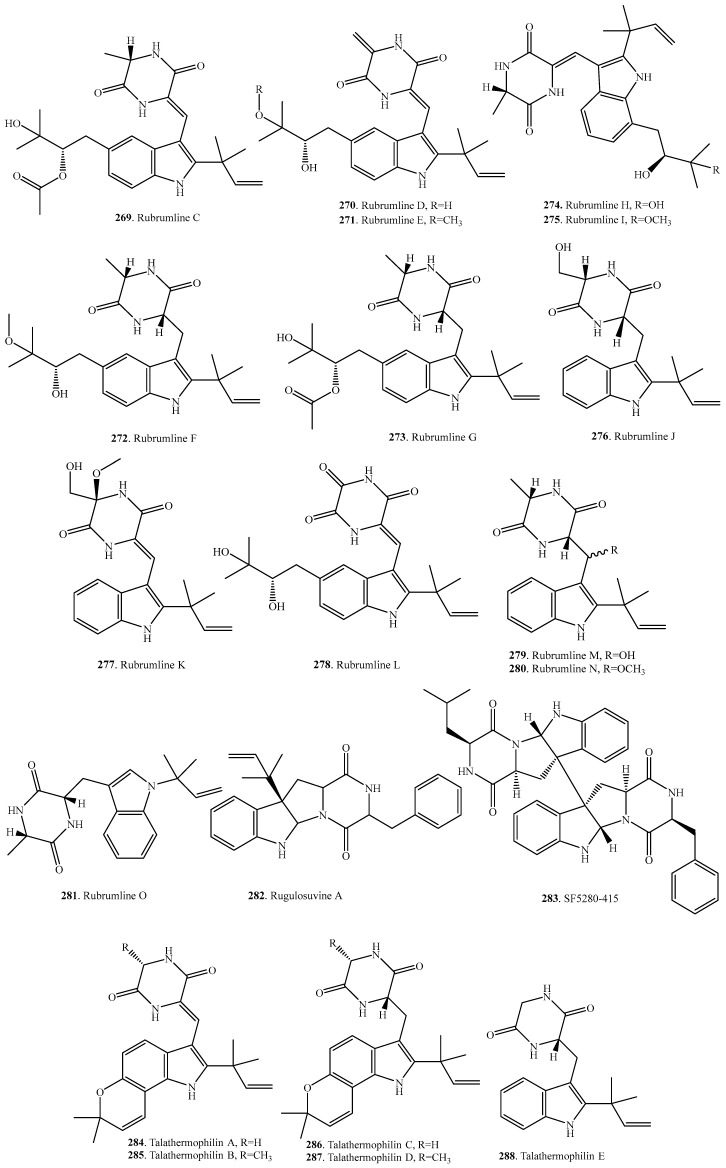

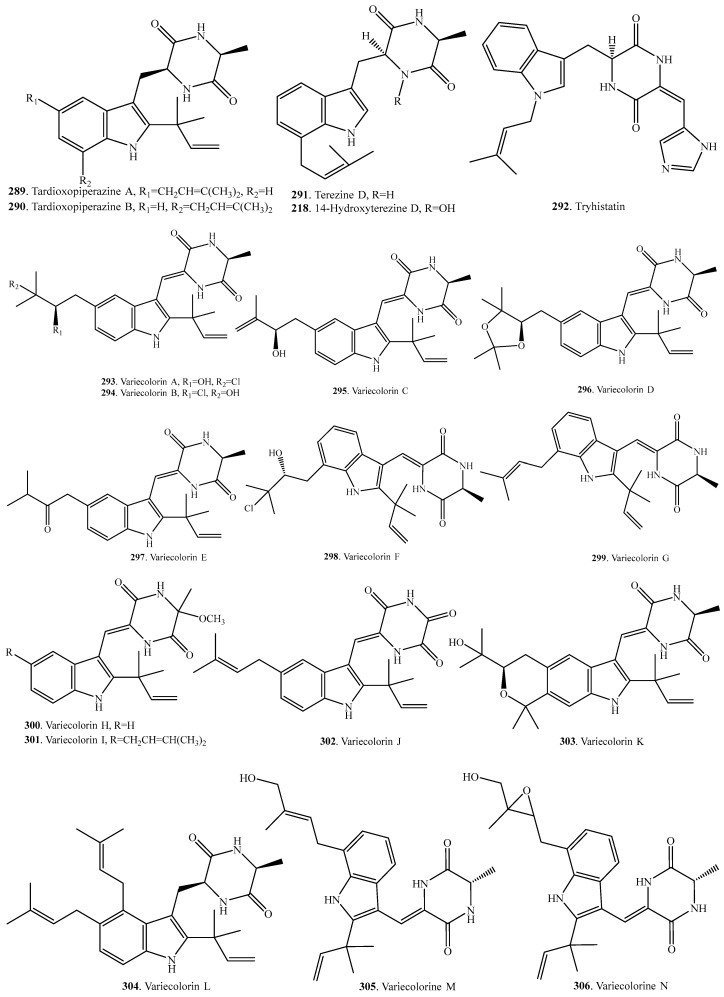

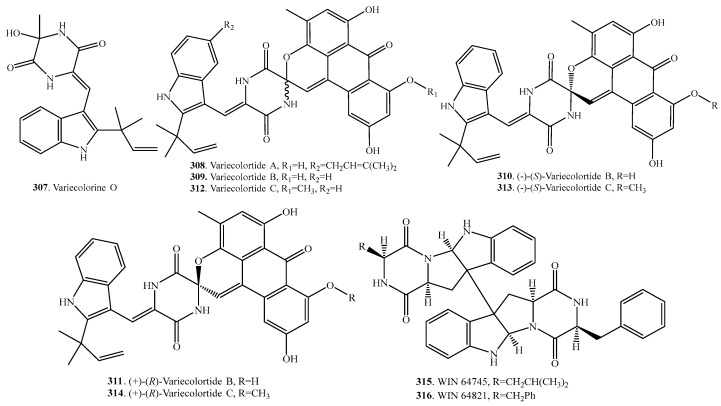
Structures of the tryptophan-Xaa cyclodipeptide analogs isolated from fungi.

Apart from Trp–Pro and Trp–Trp cyclodipeptides, other tryptophan cyclodipeptides are also abundant in fungi and represent a structurally diverse group of natural products. Their occurrence and biological activities are shown in Table 3, and their structures are provided in Figure 3.

Aspertryptanthrins A–C (**156**–**158**) were obtained from a terrestrial-derived fungus *Aspergillus* sp. These compounds all contain an anthranilate unit and a tryptophan residue. In addition, aspertryptanthrin C (**158**) contains a rare 16-membered ring [74].

The cyclodipeptides brevicompanine C (**163**), cyclo(l-Trp–d-Ile) (**182**) and cyclo(l-Trp–d-Leu) (**183**) from *Penicillium brevi-compactum* [75], as well as cyclo(l-Trp–l-Phe) (**187**) from *Penicillium* sp. [76] accelerated the root growth of the lettuce seedlings in proportion to their concentrations from 1 to 100 mg/L.

Brevicompanines D–H (**164**–**168**) were isolated from a deep ocean sediment derived fungus *Penicillium* sp. [77]. Both brevicompanines E (**165**) and H (**168**) inhibited lipopolysaccharide (LPS)-induced nitric oxide production in BV2 microglial cells. Further studies showed that brevicompanine E (**165**) reduced lipopolysaccharide-induced production of pro-inflammatory cytokines and enzymes in microglia by inhibiting activation of activator protein-1 and nuclear factor κB and, hence, may be potentially useful for modulating neuroinflammation [78].

Cycloechinulin (**190**) was isolated from the sclerotia of *Aspergillus ochraceus*, and it showed moderate insecticidal activity against the lepidopteran crop pest *Helicoverpa zea* [57].

Echinulin (**206**) is one of the simplest classes of isoprenylated tryptophan cyclodipeptides. It was toxic to rabbits, producing a significant degree of damage to lung and liver [79].

Fructigenines A (**211**) and B (**212**) are annulated derivatives of cyclo(l-Trp–l-Phe) (**187**), and were isolated from *Penicillium fructigenum*. Only fructigenine A (**211**) inhibited growth of *Avena coleoptiles* and L-5178Y (mouse lymphoma cells), and was subsequently found to have more potent anti-inflammatory activity than indomethacin in the mouse ear edema model [80]. Fructigenine A (**211**) was also named rugulosuvine B (**211**) in *Penicillium rugulosum*, and showed potent anti-inflammatory and antitumor activities in vitro [81].

Neoechinulin A (**234**) had scavenging, neurotrophic factor-like and antiapoptotic activities. The protective properties of neoechinulin A (**234**) against SIN-1-induced neuronal cell death suggested that neoechinulin A (**234**) could protect against neuronal cell death in neurodegenerative diseases [82].

The valine analog polanrazine A (**250**) was isolated from the blackleg fungus *Phoma lingam* (teleomorph: *Leptosphaeria maculans*). This compound was toxic to canola (*Brassica napus* and *B. rapa*) [83].

Rubrumlines A–O (**267**–**281**) were isolated from the marine-derived fungus *Eurototium rubrum*. Among them, rubrumlines D (**270**), F (**272**), G (**273**), J (**276**), M (**279**), N (**280**), and O (**281**) showed inhibitory activity against influenza A/WSN/33 virus. Further analysis of the structure–activity relationship revealed that the analogs with an isoprenyl unit in indole ring displayed stronger cytotoxic effects than those linked by an oxygenated isoprenyl unit. Neoechinulin B (**235**) was also isolated from *Eurototium rubrum*. This compound was efficient in inhibiting influenza A/WSN/33 virus propagation even after the fifth passage. In addition, it exerted potent inhibition against H1N1 virus infected in MDCK cells, and was able to inhibit a panel of influenza virus strains including amantadine- and oseltamivir-resistant clinical isolates. The high potency and broad-spectrum activities against influenza viruses with less drug resistance made neoechinulin B (**235**) a new lead for the development of potential inhibitor of influenza viruses [84].

The prenylated pyranoindole derivatives talathermophilins A (**284**) and B (**285**) were isolated from a thermophilic fungus *Talaromyces thermophilus* strain YM1-3. Both talathermophilins A (**284**) and B (**285**) showed nematicidal activity toward the worms of the free-living nematode *Panagrellus redivevus* [85].

Variecolorins A–L (**293**–**304**) were isolated from halotolerant fungus *Aspergillus variecolor* [80], and variecolorins M–O (**305**–**307**) from deep ocean sediment-derived fungus *Penicillium griseofulvum* [86]. They all showed weak radical scavenging activity against DPPH [86,87].

## 5. Proline–Xaa Cyclodipeptides

**Figure 4:**
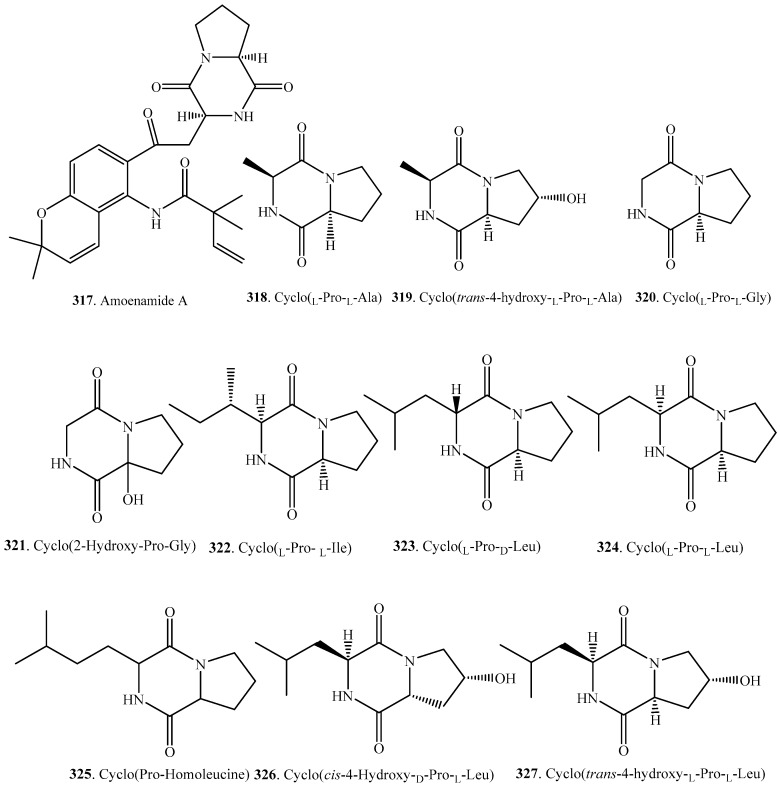

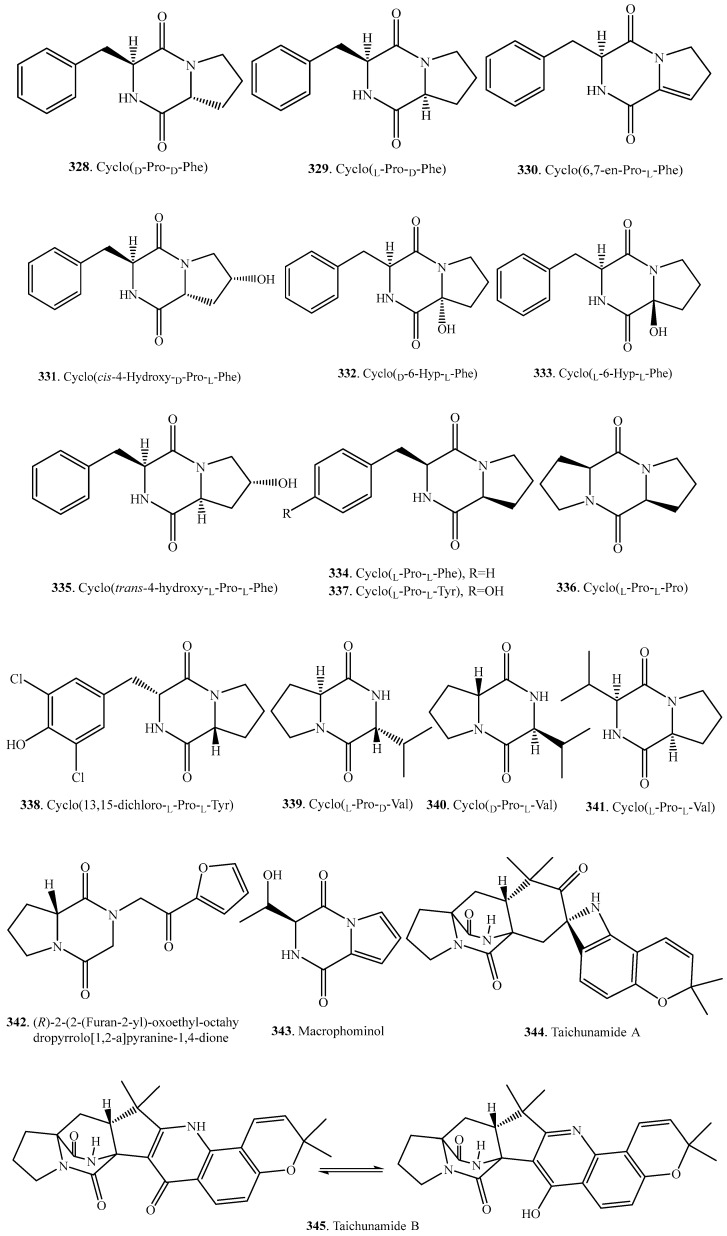
Structures of the proline-Xaa cyclodipeptide analogs isolated from fungi.

Except Trp–Pro cyclodipeptides, other proline containing cyclodipeptides (Pro–Xaa) are also abundantly distributed in fungi. Their occurrence and biological activities are shown in Table 4, and their structures are provided in Figure 4.

Cyclo(l-Pro–l-Ala) (**318**) was isolated from *Alternaria alternata* [149] and the phytopathogenic fungus *Colletotrichum gloesporoides* [150]. This compound inhibited aflatoxin production in aflatoxigenic fungi without affecting fungal growth. Further investigation on the mode of action suggested that this cyclodipeptide inhibited aflatoxin biosynthesis by affecting glutathione *S*-transferase (GST) function in *Aspergillus flavus* to show its potency as the biocontrol agent [151].

Cyclo(l-Pro–l-Phe) (**334**) and cyclo(l-Pro–l-Tyr) (**337**), which were also called maculosin-2 (**334**) and maculosin-1 (**337**), were host-specific fungal phytotoxins produced by *Alternaria alternata* on spotted knapweed (*Centaurea maculosa*) to show their protential as the bioherbicides [149].

The dechlorinated cyclodipeptide, cyclo(13,15-dichloro-l-Pro–l-Tyr) (**338**), isolated from the fungus *Leptoxyphium* sp. is considered as an inhibitor of monocyte chemotactic protein-1 (CCL2)-induced chemotaxis and was 10- to 20-fold more active than the nonchlorinated from maculosin-1 (**337**). In addition, no cellular toxicity was observed when cyclo(13,15-dichloro-l-Pro–l-Tyr) (**338**) at 100 μM was in contact with human monocyte culture for 24 h, suggesting its potential as a lead structure for the future development of anti-inflammatory compounds [152].

## 6. Non-Tryptophan–Non-Proline Cyclodipeptides

**Figure 5:**
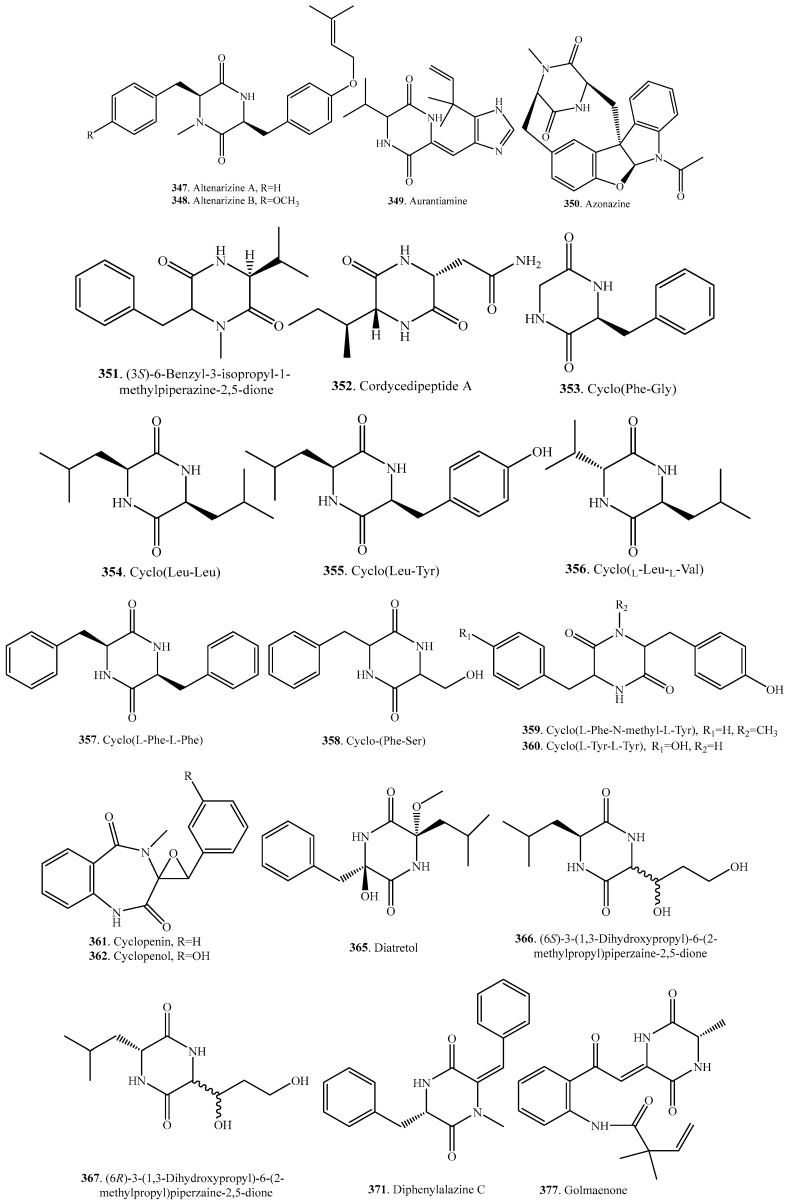

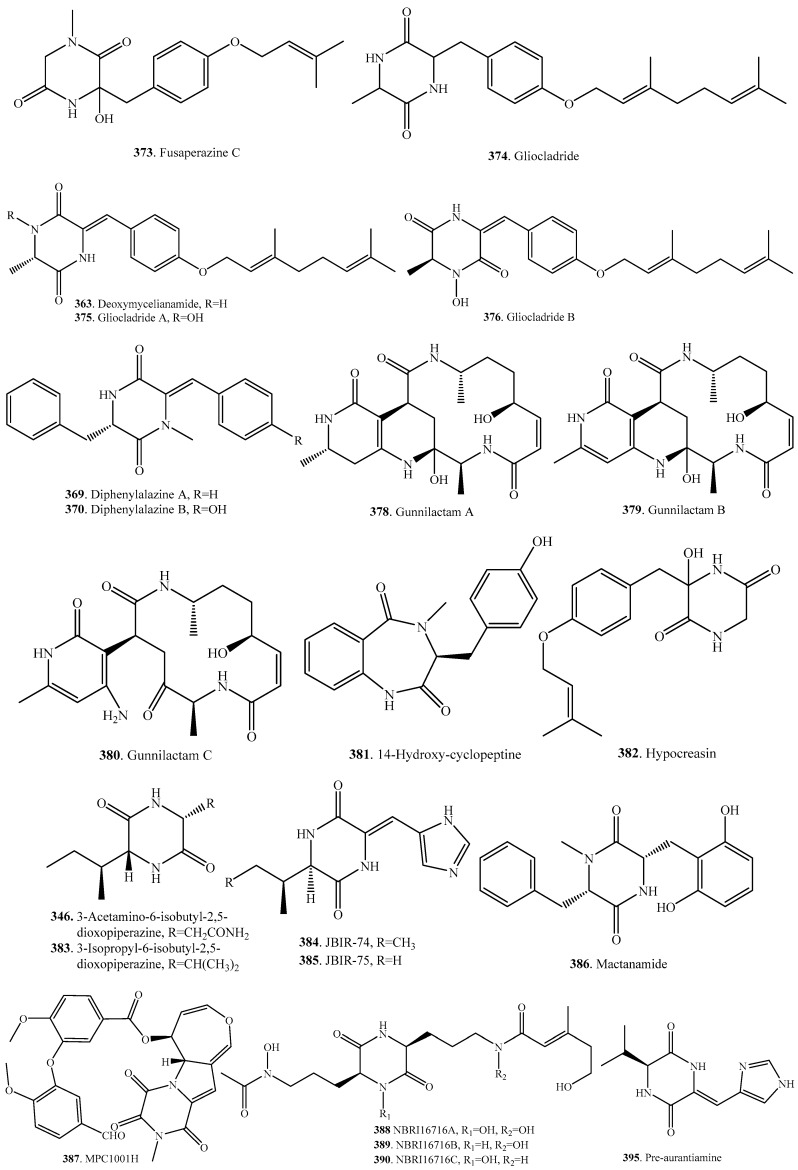

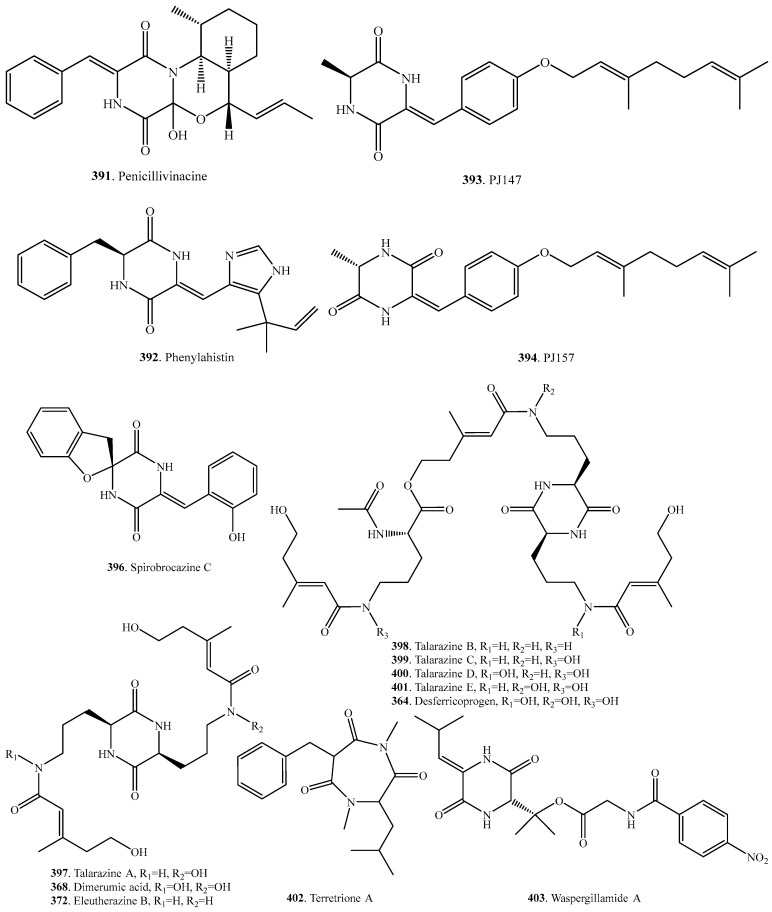
Structures of the non-tryptophan-non-proline cyclodipeptide analogs isolated from fungi.

Non-tryptophan–non-proline cyclodipeptides mean neither tryptophan nor proline is incorporated into this group of cyclodipeptides in the fungi. Their occurrence and biological activities are shown in Table 5, and the structures are provided in Figure 5.

3-Acetamino-6-isobutyl-2,5-dioxopiperazine (**346**) and 3-isopropyl-6-isobutyl-2,5-dioxopiperazine (**383**), belonging to the aliphatic isoleucine cyclodipeptide, were isolated from *Cordyceps sinensis*. Only 3-acetamino-6-isobutyl-2,5-dioxopiperazine (**346**) had cytotoxic activity against L-929, A375, and HeLa cells [169].

Azonazine (**350**) was isolated from a Hawaiian marine sediment-derived fungus *Aspergillus insulicola*, and exhibited anti-inflammatory activity by inhibiting NF-κB luciferase (IC_50_, 8.37 μM) and nitrate production (IC_50_, 13.7 μM) [170].

Cyclo(l-Phe–l-Phe) (**357**) originally isolated from *Penicillium nigricans* was also isolated from a marine mangrove endophytic fungus [171], and exhibited good anthelmintic activity against *Hymenolepis nata* and *Schistosoma mansoni* in mice [172].

Cyclic phenylalanyl serine cyclo(Phe–Ser) (**358**) was isolated from the insect pathogenic fungus *Verticillium hemipterigenum*. It exhibited concentration-dependent atypical intestinal absorption in the small intestine of rats, which consisted of passive transport, carrier-mediated absorptive transport by PEPT1, and carrier-mediated excretive transport. It also exhibited weak inhibition of several cancer cell lines and selected microorganisms [173,174].

The tyrosine analog cyclo(l-Tyr–l-Tyr) (**360**) isolated from the culture broth of *Cordyceps sinensis* reversibly blocked voltage-dependent L-type calcium channels [169].

Diatretol (**365**) from the fungus *Clitocybe diatreta* exhibited a weak antibacterial activity. A single-crystal X-ray analysis showed that diatretol (**365**) has a nearly planar boat conformation in the solid state [175].

Dimerumic acid (**368**) has been isolated from the fungus *Monascus anka*, traditionally used for fermentation of food, and shown to be an antioxidant with hepatoprotective actions against chemically induced liver injuries [176], as well as protecting against oxidative stress-induced cytotoxicity in the isolated rat hepatocytes [177].

Gliocladride (**374**) isolated from marine fungus *Gliocladium* sp. showed a cytotoxic effect with an IC_50_ value of 3.86 mg/mL against human A375-S2 melanoma cell line [178].

Gliocladrides A (**375**) and B (**376**) as well as deoxymycelianamide (**363**) were isolated from the marine fungus *Gliocladium* sp. to show cytotoxic activity against the three cell lines (HL-60, U937 and T47D) with IC_50_ values 11.6–52.8 μg/mL, while deoxymycelianamide (**363**) showed the strongest cytotoxic activity against U937 cell line with an IC_50_ value of 0.8 μg/mL [179].

Golmaenone (**377**) from the culture broth of the marine-derived fungus *Aspergillus* sp. exhibited a significant radical scavenging activity against 1,1-diphenyl-2-picrylhydrazyl (DPPH) and showed UV-A (320–390 nm) protecting activity which was more active than oxybenzone currently used as a sunscreen [103].

The marine-derived fungus *Aspergillus* sp. yielded mactanamide (**386**) containing an *R*-2,6-dihydroxyphenylalanine, which showed fungistatic activity to *Candida albicans* at nontoxic concentration [180].

Three siderophores NBRI16716A (**388**), NBRI16716B (**389**), and NBRI16716C (**390**) were isolated from the fungus *Perisporiopsis melioloides* Mer-f16716. Compounds NBRI16716A (**388**) and NBRI16716B (**389**) inhibited the growth of human prostate cancer DU-145 cells in the coculture with human prostate stromal cells (PrSCs) more strongly than that of DU-145 cells alone. Furthermore, both compounds showed antitumor effect against xenograft models of DU-145 cells and PrSCs in vivo [181].

Phenylahistin (**392**) from the culture broth of *Aspergillus ustus* NSC-F038 exhibited a strong growth inhibition on various tumor cell lines for its microtubule binding function to show its potency as the tubulin depolymerizing agent [182].

## 7. Thio-Cyclodipeptides

The thio-cyclodipeptides are 2,5-diketopiperazines containing thio functionality in bridged or open form, and their occurrence and activities have been reviewed in 2006 and 2014 [1,207]. According to the positions of the sulfur linkages, we divide thio cyclodipeptides into four subgroups: 1,4-bridged epipolythioxopiperazines (ETPs), derivatives with sulfur-bridge outside 2,5-DKP ring, nonbridged dimethylthio derivatives, and other sulfur-containing cyclodipeptides. About 232 thio-cyclodipeptides have been isolated from fungi.

### 7.1. 1,4-Bridged Epiplythiodioxopiperazine Analogs

**Figure 6:**
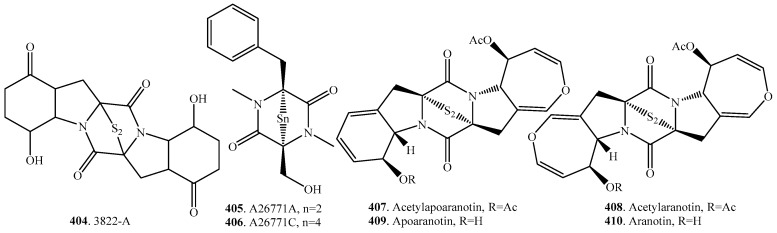

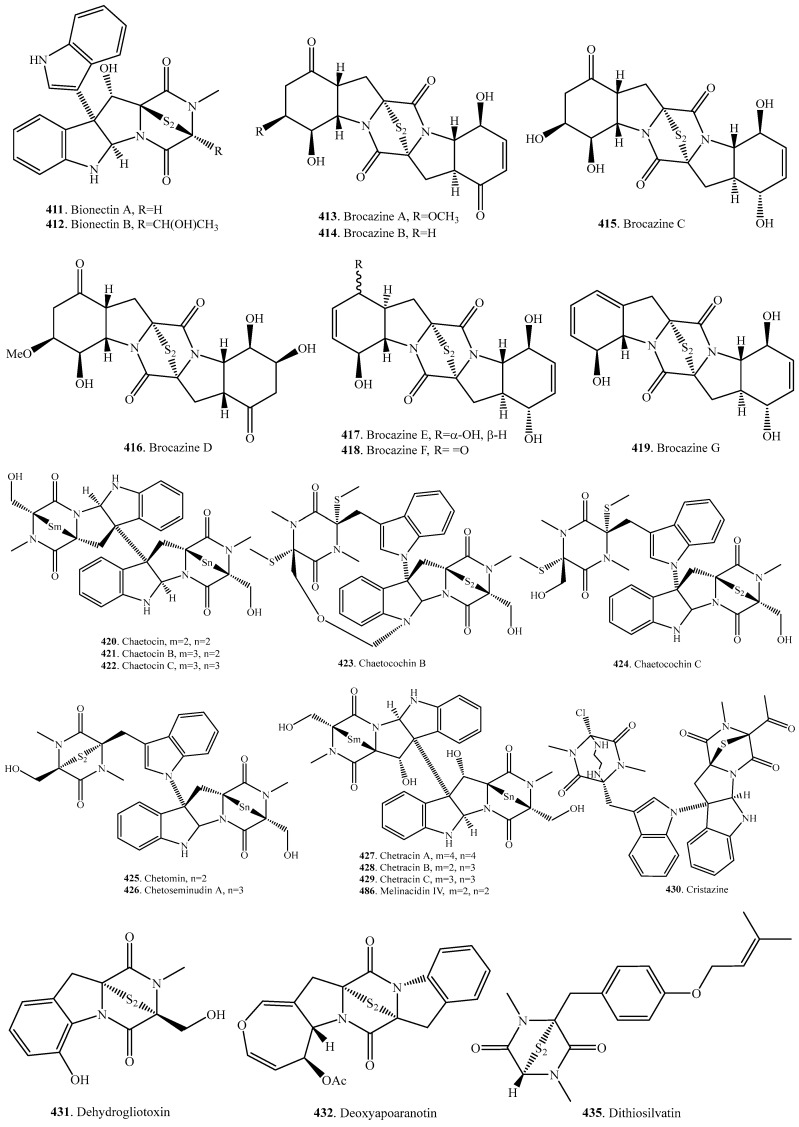

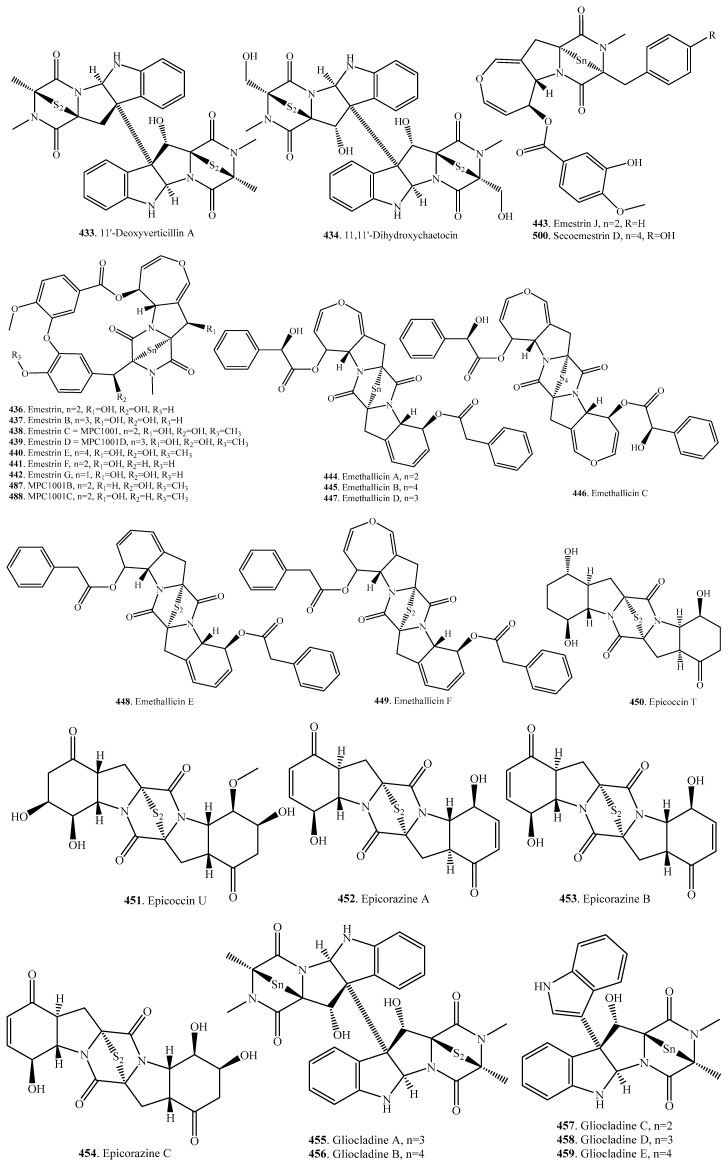

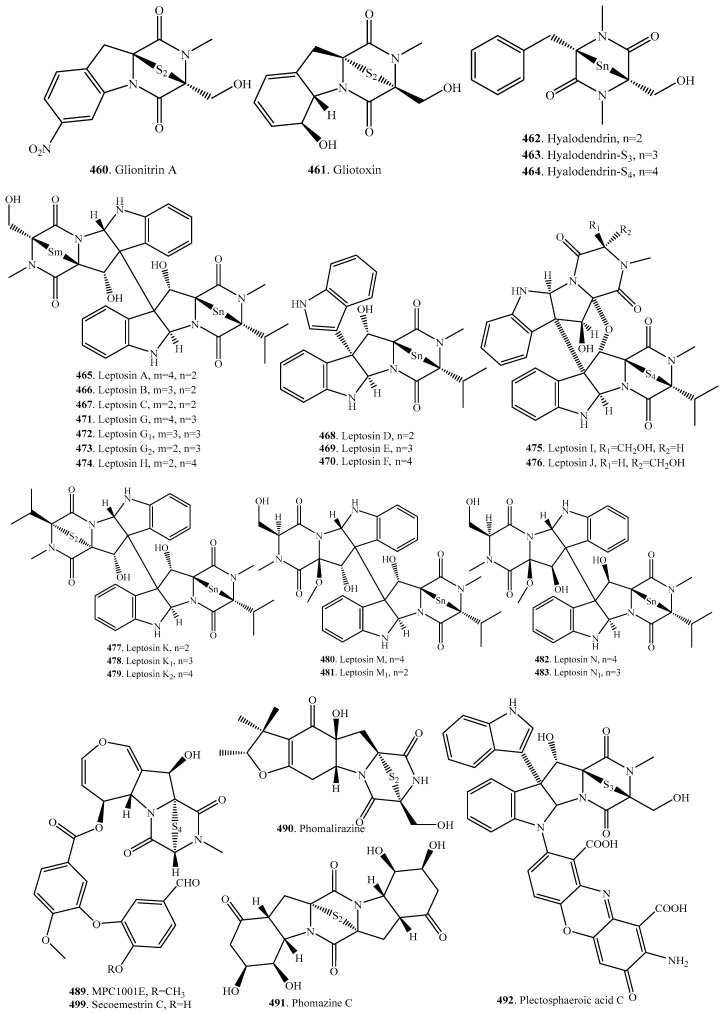

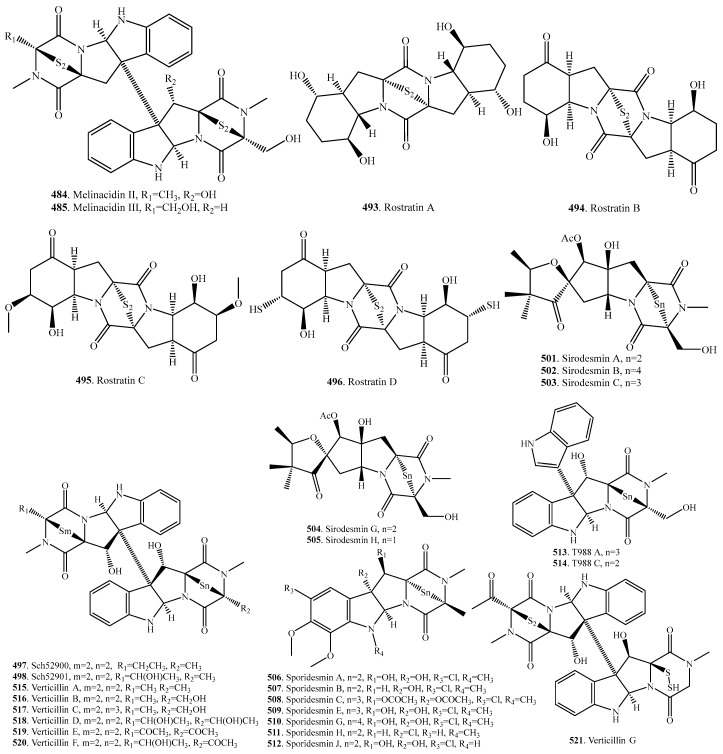
Structures of the 1,4-bridged epiplythiodioxopiperazine cyclodipeptide analogs isolated from fungi.

The sulfur-bridged cyclodipeptides are a class of metabolites mainly dominated by the epipolythiodioxopiperazines (ETPs) [1]. The toxicity of ETPs is due to the presence of a sulfide bridge, which can inactivate proteins via reaction with thiol groups and by generation of reactive oxygen species by redox cycling [5]. The ETPs are known for their cytotoxic effect on cancer cell lines, and it has been shown mechanistically that ETPs were transcriptional antagonists that block the interaction of the p300/CBP coactivator with the hypoxia-inducible transcription factor HIF-1α by a zinc ejection mechanism, which resulted in rapid down regulation of hypoxia-inducible genes critical for cancer progression [208]. The occurrence and biological activities of 1,4-bridged epiplythiodioxopiperazine analogs from fungi are shown in Table 6, and their structures are provided in Figure 6.

The dimeric ETP chaetocin (**420**) was isolated from the fungus *Chaetomium minutum*, and, in addition to its antibacterial and cytostatic activity, was reported to have inhibitory activity against lysine-specific histone methyltransferases (HMTs), which are key enzymes in the epigenetic control of gene expression [209]. Chaetocins B (**421**) and C (**422**) from a *Chaetomium* sp. fermentation broth were potent inhibitors of *Staphylococcus aureus*, and exhibited potent cytotoxic activity against HeLa cells with IC_50_ values of 0.03 and 0.02 μg/mL, respectively [210].

Chaetocochins B (**423**) and C (**424**), which were isolated from *Chaetomium cochliodes*, showed cytotoxic activity to the cells of Bre-04 (breast cancer cells), Lu-04 (big cell lung cells), and N-04 (glioma cells) [211].

Three ETPs deoxyapoaranotin (**432**), acetylaranotin (**408**) and acetylapoaranotin (**407**) were isolated from *Aspergillus* sp. KMD 901 found in the marine sediment obtained from the East Sea of Korea. They had directly cytotoxic and apoptosis inducing effects toward HCT116 colon cancer cell lines [212].

Emethallicins A–F (**444**–**449**) were obtained from *Emericella heterothallica*. These compunds all displayed inhibitory activity on histamine release from mast cells [213,214,215].

The best known ETP was the small lipid-soluble gliotoxin (**461**), which exerted toxic effects on phagocytic cells and T-lymphocytes at low concentrations in vitro. This compound was the first ETP to be obtained from fungi. It has been isolated from a variety of fungi including species in the genera of *Penicillium*, *Aspergillus*, *Gliocladium*, *Thermoascus*, and *Candida* [2]. High levels of gliotoxin (**461**) were produced by *Aspergillus fumigatus* in vivo, and it appeared to be a virulence factor associated with invasive aspergillosis of immunocompromised patients [216]. Gliotoxin (**461**) was also a dual inhibitor of farnesyltransferase and geranylgeranyltransferase I with antitumor activity against breast cancer in vivo [217].

Two bridged disulfides epicoccin T (**450**) and rostratin A (**493**) were isolated from the endophytic fungus *Epicoccum nigrum* obtained from the leaves of *Lysidice rhodostegia*. However, they did not show detectable cytotoxic activities toward six tumor cell lines in the 3-(4,5-dimethylthiazol-2-yl)-2,5-diphenyltetrazolium bromide (MTT) assay [188]. In contrast, the rostratins A–D (**493**–**496**), which were extracted from *Exserohilum rostratum*, were found to be modestly cytotoxic against human colon carcinoma HCT-116 [218]. Similarly, the unsaturated analogs epicorazines A (**452**), B (**453**), and C (**454**) isolated from the culture broth of the basidiomycete *Stereum hirsutum* HKI 0195 were highly cytotoxic against HeLa cells and also exhibited antiproliferative effects against several mouse fibroblast and cancer cell lines [219].

The antifungal macrolide MPC1001 (**438**) possessing an ETP ring was isolated from the fungus *Cladorrhinum* sp. KY4922, which was found in a soil sample collected in Indonesia. It possessed antiproliferative activity against the human prostate cancer cell line (DU145) with an IC_50_ of 9.3 nM, and had antibacterial activity against Gram-positive bacteria [220].

Secoemestrin D (**500**) was obtained from the endophytic fungus *Emericella* sp. AST0036 isolated from a healthy leaf tissue of *Astragalus lentiginosus*. This compound showd cytotoxic activity with IC_50_ values ranging from 0.06 to 0.24 μM on tumor cell lines [221].

Verticillin A (**515**) was obtained from *Gliocladium roseum* derived from submerged wood. It showed antinematodal activity against *Panagrellus redivivus* and *Caenorhabditis elegans*, and cytotoxic activity against HeLa cells with an IC_50_ value of 0.2 μg/mL [222]. Verticillins B (**516**) and C (**517**) were isolated from *Verticillium* sp. [223]. Verticillin D (**518**), which was isolated from *Bionectria byssicola* [222] and *Gliocladium catenulatum* [224], showed antibacterial activity against *Staphylococcus aureus*. Verticillins E (**519**) and F (**520**) from *Gliocladium catenulatum* showed antibacterial activity [224]. Verticillin G (**521**) from *Bionectra byssicola* showed antibacterial activity against *Staphylococcus aureus* including methicillin-resistant and quinolone-resistant varieties with minimum inhibitory concentrations (MICs) of 3–10 μg/mL [225].

### 7.2. Analogs with Sulfur-Bridge outside 2,5-DKP Ring

**Figure 7:**
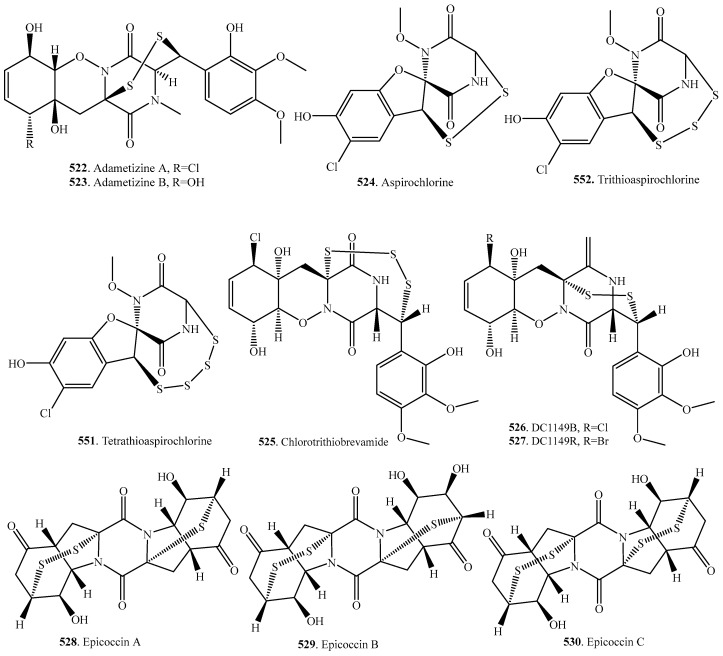

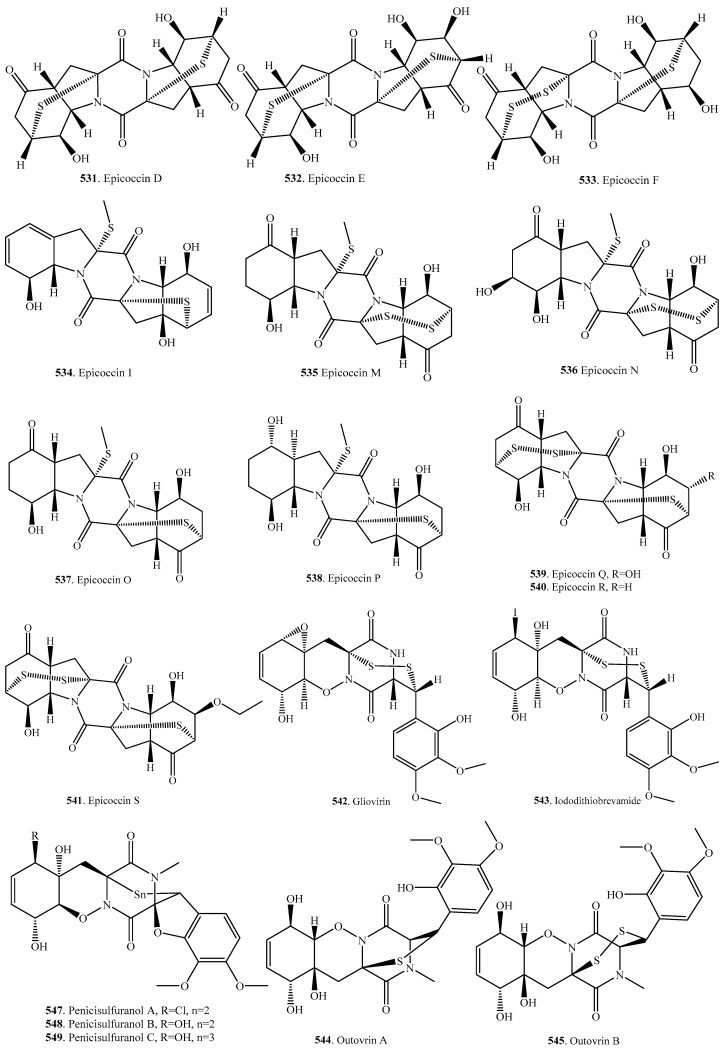

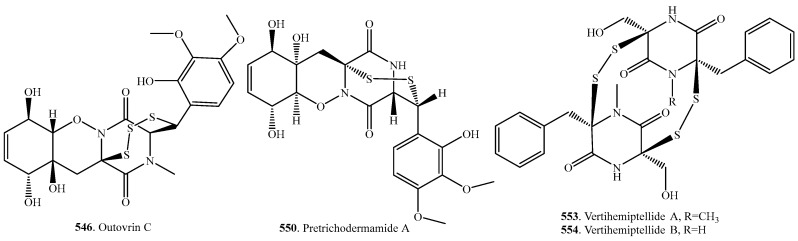
Structures of the analogs with sulfur-bridge outside 2,5-DKP ring isolated from fungi.

There are a group of sulfur-bridged cyclodipeptides where the sulfur linkage is outside the 2,5-diketoperazine ring. The occurrence and biological activities of fungal analogs with sulfur-bridges outside 2,5-DKP ring are shown in Table 7, and their structures are provided in Figure 7.

Both aspirochlorine (**524**) and tetrathioaspirochlorine (**551**) were isolated from *Aspergillus flavus* and were potent antifungals that inhibited azole-resistant *Candida albicans* [107]. Aspirochlorine (**524**) was a rather potent and selective inhibitor of fungal protein synthesis that did not inhibit bacterial or mammalian protein synthesis [267].

Epicoccins are diannulated 2,5-DKPs containing mono- or bis-cross ring sulfide/disulfide bridges. Epicoccins A–F (**528**–**533**) have been isolated from the solid-substrate fermentation culture of the *Cordyceps*-colonizing fungus *Epicoccum nigrum* [194,268]. Epicoccin A (**528**) showed modest antimicrobial activity against *Bacillus subtilis* [268].

The dimeric 2,5-DKPs vertihemiptellides A (**553**) and B (**554**) were isolated from the insect pathogenic fungus *Verticillium hemipterigenum*, and exhibited growth inhibitory activity against *Mycobacterium tuberculosis* H37Ra, and also showed moderate cytotoxic activity [174].

### 7.3. Nonbridged Methylthio-Containing Cyclodipeptide Analogs

**Figure 8:**
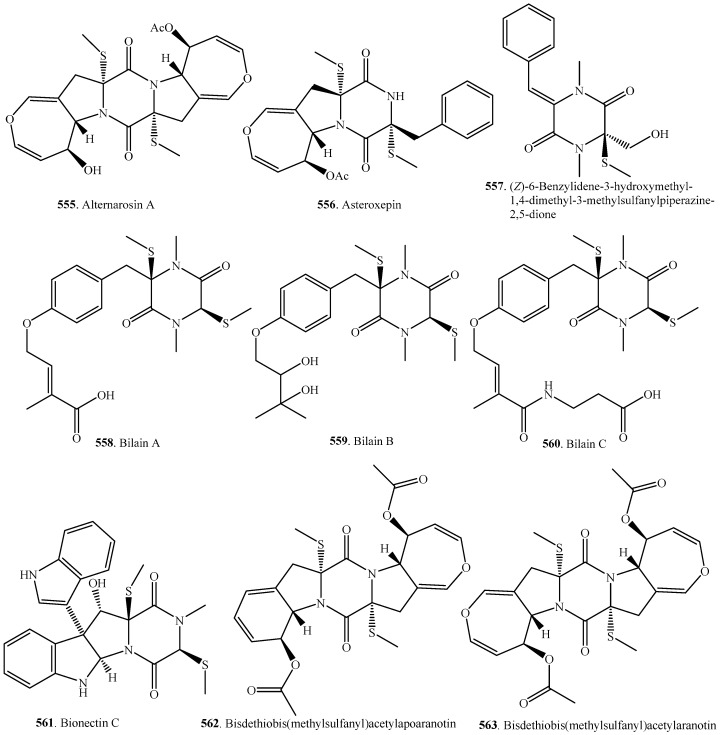

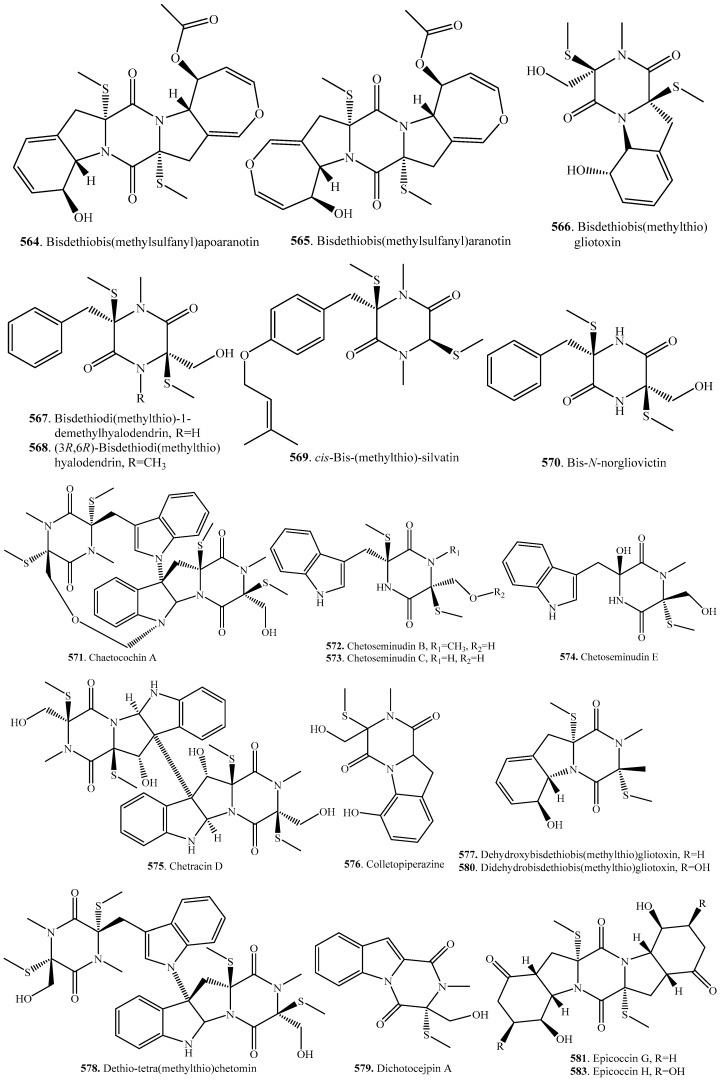

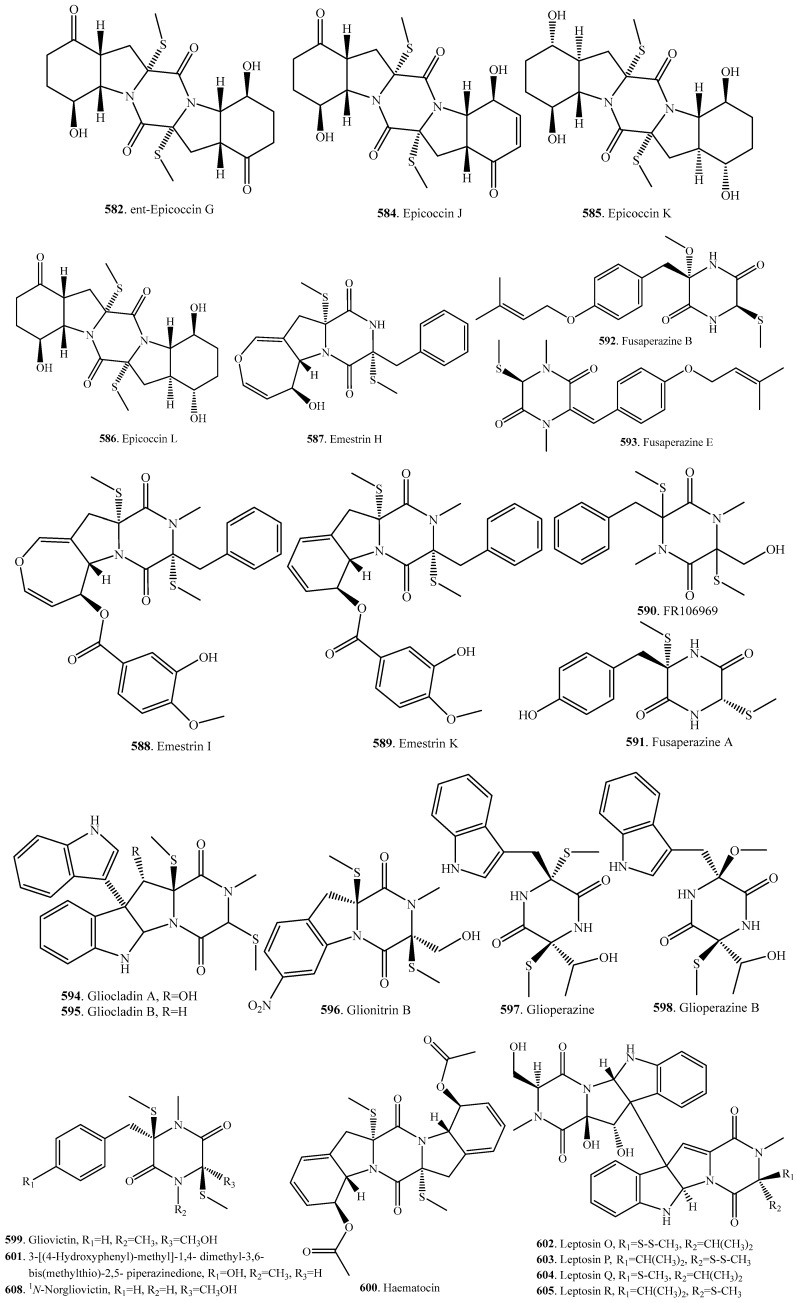

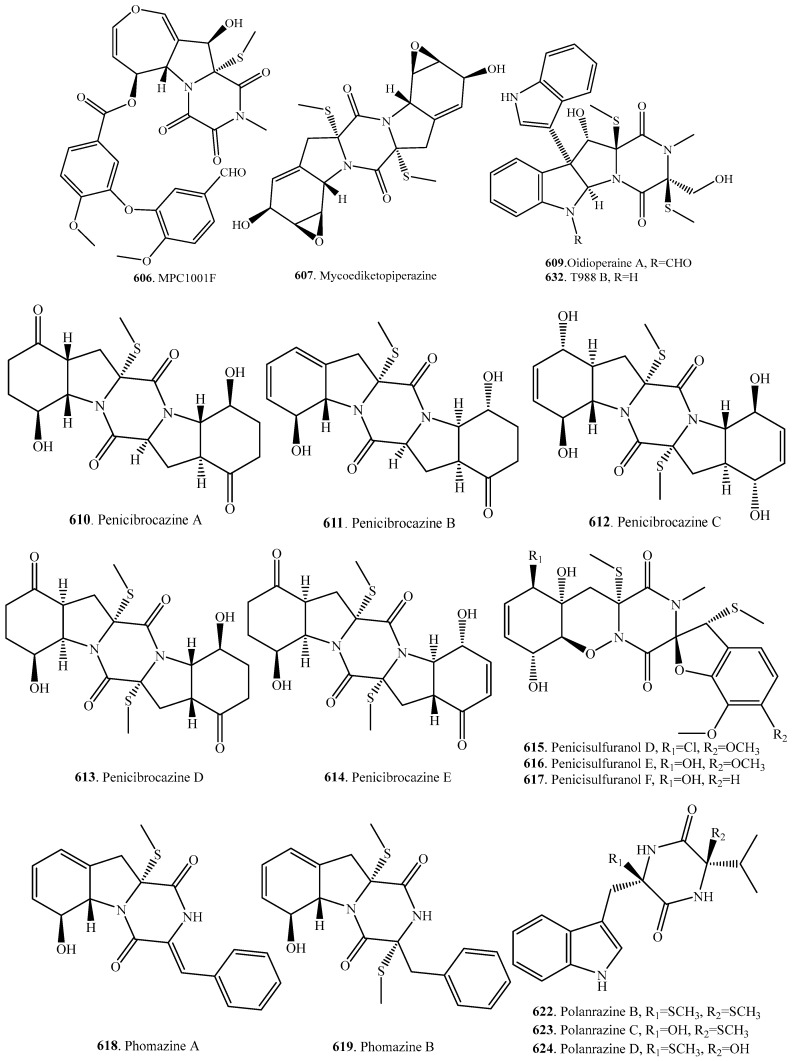

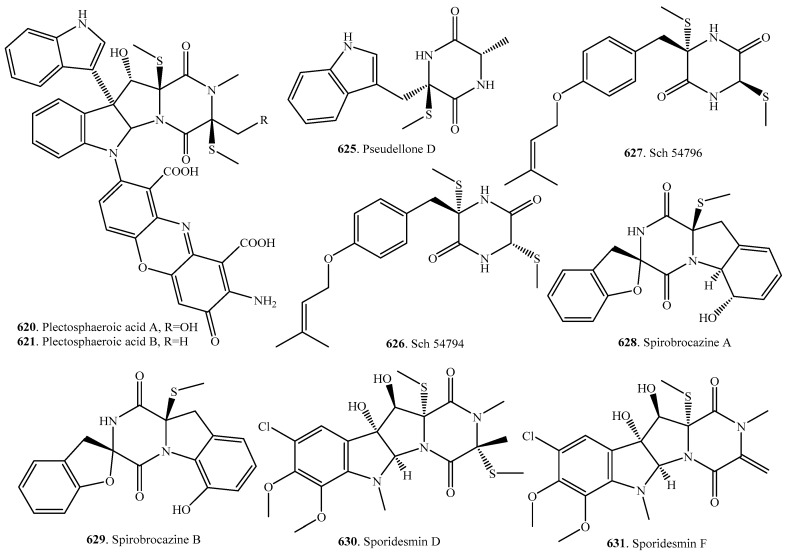
Structures of the nonbridged methylthio-containing cyclodipeptide analogs isolated from fungi.

Nonbridged methylthio-containing analogs were often isolated from fungi as co-metabolites with their sulfur-bridge 2,5-DKP parents. They had a related biosynthetic pathway [275]. The occurrence and biological activities of this group of fungal metabolites are shown in Table 8, and their corresponding structures are provided in Figure 8.

Alternarosin A (**555**) from *Alternaria raphanin*, a halotolerant marine fungus obtained from the sediment of the Hongdao sea salt field, showed very weak antimicrobial activity against *Escherichia coli*, *Bacillus subtilis*, and *Candida albicans* with MIC values ranging from 200 to 400 μM [276].

Three 2,5-DKPs, bilains A–C (**558**–**560**) and *cis*-bis(methylthio)silvatin (**480**) were isolated from the marine-derived fungus *Penicillium bilaii* collected in Tasmania. However, only *cis*-bis(methylthio)silvatin (**569**) showed weak cytotoxicity against NS-1 cells [161]. Both *cis*-bis(methylthio)silvatin (**569**) and its enantiomer Sch 54794 (**524**) were previously isolated from the fungus *Fusarium chlamydosporum* OUPS-N124 obtained from the marine alga *Carpopeltis affinis*. They exhibited weak cytotoxic activity against P388 lymphocytic leukemia cells [277].

Bis-*N*-norgliovictin (**570**) was first isolated from *Gliocladium virens* [233]. It was also isolated from three marine-derived fungi *Aspergillus fumigatus* [278], *Neosartorya pseudosidcheri* [136], and *Dichotomomyces* sp. L-8 [113]. Bis-*N*-norgliovictin (**570**) significantly inhibited lipopolysaccharide (LPS)-induced inflammation in macrophages and improved survival in sepsis, and it should be a therapeutic candidate for the treatment of sepsis and other inflammatory diseases [279].

Dehydroxybisdethiobis(methylthio)gliotoxin (**577**) has been isolated from the broth of a marine-derived fungus *Pseudallescheria* sp. and exhibited weak antibacterial activity against methicillin-resistant and multidrug-resistant *Staphylococcus aureus* with MIC values of 31.2 μg/mL [280].

Ent-epicoccin G (**582**) from the endophytic fungus *Epicoccum nigrum* showed potent in vitro activity (IC_50_, 3.07 μM) against the release of *β*-glucuronidase in rat polymorphonuclear leukocytes induced by platelet-activating factor [188].

The bis(methylthio)-2,5-DKP FR106969 (**590**) isolated from *Penicillium citrinum* showed high inhibitory activity against the platelet activating factor (PAF)-induced rabbit platelet aggregation to have its potency as the anti-inflammatory inhibitor [281].

The indole derivatives gliocladins A (**594**) and B (**595**) as well as glioperazine (**597**) have been obtained from a marine-derived fungus *Gliocladium roseum* PS-N132 isolated from the sea hare *Aplysia qkurodai*. All three 2,5-DKPs exhibited cytotoxicity against murine P388 lymphocytic leukemia cells [125].

Both haematocin (**600**) and mycoediketopiperazine (**607**) were dimethylthio 2,5-DKPs. Haematocin (**600**) was isolated from the phytopathogenic fungus *Nectria haematococca* and had inhibitory activity on the spore germination and germ-tube elongation of *Magnarporte oryzae* [282]. Mycoediketopiperazine (**607**) from the fungus *Papularia* sp. exhibited potent cytotoxic activity on KB cells with an IC_50_ value of 120 μg/mL [283].

Plectosphaeroic acids A (**620**) and B (**621**) were obtained from marine-derived fungus *Plectosphaerella cucumerina*. They were inhibitors of indoeamine 2,3-dioxygenase (IDO), which existed in primary tumor cells. IDO has been considered as an important molecular target for cancer therapy [254].

### 7.4. Other Sulfur-Containing Cyclodipeptide Analogs

**Figure 9:**
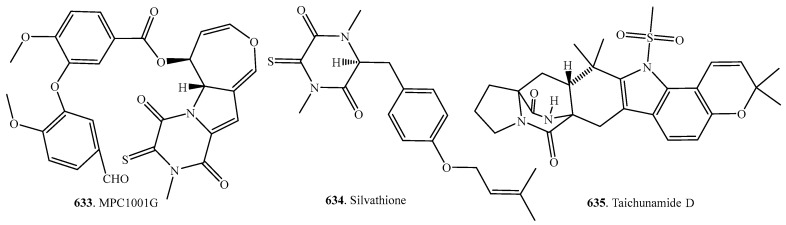
Structures of the other sulfur-containing cyclodipeptide analogs isolated from fungi.

Other sulfur-containing cyclodipeptide analogs included MPC1001G (**633**), silvathione (**634**) and taichunamide D (**635**) with their structures shown in Figure 9. MPC1001G (**633**) was isolated from *Cladorrhinum* sp. KY4922 [236], silvathione (**634**) from *Aspergillus silvaticus* [235], and taichunamide D (**635**) from *Aspergillus taichungensis* IBT 19404 [53]. Their biological activities have not been reported.

## 8. Conclusions and Future Perspectives

A large number of cyclodipeptides have been identified in fungi, and many have received attention not only as challenging synthetic targets but also because some of these compounds displayed diverse and interesting biological activities. Since then, interest has increased in the biosynthesis, genetics, total synthesis, biological activities, and medicinal properties of this class of natural products. Some cyclodipeptides such as tryprostatins A (**103**) and B (**104**), cyclo(l-Pro–l-Ala) (**318**), cyclo(l-Pro–l-Phe) (**334**), cyclo(l-Pro–l-Tyr) (**337**), phenylahistin (**392**), and FR106969 (**590**) have displayed their potential applications in agriculture and medicinal industry [16,17,149,151,182,281].

The fungal cyclodipeptides are mainly distributed in the genera of *Aspergillus* and *Penicillium*. However, the cyclodipeptides in the remaining genera seem to be less explored. Further identification and exploration of the cyclodipeptides from all of the fungal genera are needed. In recent years, more and more cyclodipeptides have been isolated from marine-derived and plant endophytic fungi [297,298,299,300]. These fungi inhabiting particular environments could be rich sources of biologically active cyclodipeptides that are indispensable for medicinal and agricultural applications.

The biological activities (shown in Table 1, Table 2, Table 3, Table 4, Table 5, Table 6, Table 7 and Table 8) of the cyclodipeptides reported by each investigator were random and limited. Systematical screening of biological activities for each cyclodipeptide should be necessary. In most cases, the biological activities as well as the mode of action of fungal cyclodipeptides have been investigated based on in vitro studies or animal modes. Few studies have been performed at the level of clinical trials in patients. Effective research and development methods for these compounds should be explored to maximize their usefulness in the drug discovery and development processes [6]. For the diverse biological activities, the cyclodipeptides from fungi are expected to inspire medicinal chemists in their search for better agents such as antitumors, antifungals, and antibacterials than existing ones [297].

It is very important to understand biosynthetic mechanisms of the cyclodipeptides in fungi. These need to combine their biochemical and genetic approaches. More and more designed biologically active cyclodipeptides will be expected to be produced by genetic manipulation. With a good understanding of the biosynthetic pathways of bioactive cyclodipeptides, we can not only increase outputs of the beneficial cyclodipeptides but also block biosynthesis of some harmful cyclodipeptides by specific interferences [6].

## Figures and Tables

**Table 1:** Fungal tryptophan-proline cyclodipeptide analogs and their biological activities.

Name	Fungus and Its Origin	Biological Activity	Ref.
Aspergamide A (**1**)	*Aspergillus ochraceus*	-	[5]
Aspergamide B (**2**)	*Aspergillus ochraceus*	-	[5]
Aspergilazine A (**3**)	Marine-derived *Aspergillus taichungensis* ZHN-7-07	Weak activity against influenza A (H_1_N_1_) virus	[18]
(+)-Austamide (**4**)	*Aspergillus ustus*	Acute toxicosis in day-old ducklings	[7]
6-*epi*-Avrainvillamide (**5**)	*Aspergillus taichungensis*	-	[19]
Brevianamide E (**6**)	Deep sea derived *Aspergillus versicolor* CXCTD-06-6a	Moderate radical scavenging activity against DPPH	[20]
Brevianamide F = Cyclo(l-Trp–l-Pro) (**7**)	Endophytic *Aspergillus fumigatus*	-	[21]
	Endophytic *Aspergillus fumigatus* from *Melia azedarach*	Plant growth inhibitory activity	[11]
	Marine-derived *Aspergillus taichungensis* ZHN-7-07	-	[18]
	Marine-derived *Penicillium vinaceum*	Antimicrobial activity	[22]
	Marine-derived *Pseudallescheria* sp. isolated from the surface of the drift wood	Antibacterial activity against *Staphylococcus aureus*	[23]
Brevianamide J (**8**)	*Aspergillus versicolor*	-	[24]
Brevianamide K (**9**)	*Aspergillus versicolor*	-	[24]
	Marine-derived *Aspergillus versicolor* from the sediment collected from the Bohai Sea of China	-	[25]
	*Aspergillus versicolor* from the marine brown alga *Sargassum thunbergii*	-	[26]
	Deep sea derived *Aspergillus versicolor* CXCTD-06-6a	Moderate radical scavenging activity against DPPH	[20]
Brevianamide Q (**10**)	*Aspergillus versicolor*	-	[27]
	Deep sea derived *Aspergillus versicolor* CXCTD-06-6a	Moderate radical scavenging activity against DPPH	[20]
Brevianamide R (**11**)	*Aspergillus versicolor*	-	[27]
	Deep sea derived *Aspergillus versicolor* CXCTD-06-6a	Moderate radical scavenging activity against DPPH	[20]
Brevianamide S (**12**)	Marine-derived *Aspergillus versicolor* from the sediment collected from the Bohai Sea of China	Selective antibacterial activity	[25]
Brevianamide T (**13**)	Marine-derived *Aspergillus versicolor* from the sediment collected from the Bohai Sea of China	-	[25]
Brevianamide U (**14**)	Marine-derived *Aspergillus versicolor* from the sediment collected from the Bohai Sea of China	-	[25]
Brevianamide V (**15**)	Marine-derived *Aspergillus versicolor* from the sediment collected from the Bohai Sea of China	-	[25]
	Deep sea derived *Aspergillus versicolor* CXCTD-06-6a	Moderate radical scavenging activity against DPPH	[20]
Brevianamide W (**16**)	Deep sea derived *Aspergillus versicolor* CXCTD-06-6a	Moderate radical scavenging activity against DPPH	[20]
5-Chlorosclerotiamide (**17**)	Deep sea derived *Aspergillus westerdijkiae*	-	[28]
Cyclo(d-Trp–l-Pro) (**18**)	Marine-derived *Penicillium vinaceum*	Antimicrobial activity	[22]
Cyclo(*N*-benzyl-Trp–Pro) (**19**)	Endophytic *Aspergillus tamari* from *Ficus carica*	-	[29]
Cyclo(*N*’-prenyl-l-Trp–l-Pro) (**20**)	Endophytic *Aspergillus fumigatus*	-	[21]
Cyclotryprostatin A (**21**)	Endophytic *Aspergillus fumigatus* from *Melia azedarach*	-	[11]
	*Aspergillus fumigatus*	Inhibitory activity at G2/M-phase of the mammalian cell cycle	[30]
	Endophytic *Aspergillus tamari* from *Ficus carica*	-	[29]
Cyclotryprostatin B (**22**)	Endophytic *Aspergillus fumigatus* from *Melia azedarach*	Plant shoot elongation inhibitory activity	[11]
	*Aspergillus fumigatus*	Inhibitory activity at G2/M-phase of the mammalian cell cycle	[30]
	Endophytic *Aspergillus tamari* from *Ficus carica*	-	[29]
Cyclotryprostatin C (**23**)	*Aspergillus fumigatus*	Inhibitory activity at G2/M-phase of the mammalian cell cycle	[30]
	Endophytic *Aspergillus tamari* from *Ficus carica*	-	[29]
Cyclotryprostatin D (**24**)	*Aspergillus fumigatus*	Inhibitory activity at G2/M-phase of the mammalian cell cycle	[30]
	Endophytic *Aspergillus tamari* from *Ficus carica*	-	[29]
12,13-Dehydroprolyltryptopha-nyldiketopiperazine (**25**)	*Penicillium piscarium*	-	[31]
Demethoxyfumitremorgin C (**26**)	*Aspergillus fumigatus*	Cytotoxic activity	[9]
Deoxybrevianamide E (**27**)	*Aspergillus* sp.	-	[32]
	Marine-derived *Aspergillus versicolor* from the sediment collected from the Bohai Sea of China	-	[25]
(+)-Deoxyisoaustamide (**28**)	*Aspergillus ustus*	-	[7]
Dihydrocarneamide A (**29**)	Marine-derived *Paecilomyces variotii*	Weak cytotoxic activity	[33]
8,9-Dihydroxyfumitremorgin C = 12,13-Dihydroxyfumitremorgin C (**30**)	Endophytic *Aspergillus fumigatus*	-	[21]
	Marine-derived *Aspergillus sydowi* from a driftwood sample	-	[10]
	Marine-derived *Aspergillus* sp.	Cytotoxic activity	[34]
	Deep-sea derived *Aspergillus* sp. SCSIO Ind09F01	Anti-tuberculosis and cytotoxic activity	[35]
	Marine-derived *Pseudallescheria* sp. isolated from the surface of the drift wood	Antibacterial activity against *Staphylococcus aureus*	[23]
*rel*-(8*S*)-19,20-Dihydro-9,20-dihydroxy-8-methoxy-9,18-di-*epi*-fumitremorgin C (**31**)	Endophytic *Aspergillus fumigatus*	-	[21]
*rel*-(8*S*,19*S*)-19,20-Dihydro-,19,20-trihydroxy-8-methoxy-9-*epi*-fumitremorgin C (**32**)	Endophytic *Aspergillus fumigatus*	-	[21]
(3*S*,8*S*,9*S*,18*S*)-8,9-Dihydroxyspirotryprostatin A (**33**)	Endophytic *Aspergillus fumigatus*	-	[21]
9ξ-*O*-2(2,3-Dimethylbut-3-enyl) brevianamide Q (**34**)	*Aspergillus versicolor* from the marine brown alga *Sargassum thunbergii*	-	[26]
(-)-Enamide (**35**)	Marine-derived *Aspergillus versicolor*	-	[36]
Fumitremorgin A (**36**)	Marine sediment-derived *Penicillium brefeldianum* SD-273	-	[37]
Fumitremorgin B (**37**)	Endophytic *Aspergillus tamari* from *Ficus carica*	-	[29]
	Endophytic *Aspergillus fumigatus* from *Melia azedarach*	Plant shoot elongation inhibitory activity	[11]
	*Aspergillus fumigatus*	-	[8]
	Endophytic *Alternaria* sp. FL25 from *Ficus carica*	Antiphytopathogenic fungal activity	[38]
Derivative of fumitremorgin B (24*R*) (**38**)	*Aspergillus fumigatus*	Cytotoxic activity	[8]
Derivative of fumitremorgin B (24*S*) (**39**)	*Aspergillus fumigatus*	Cytotoxic activity	[8]
Fumitremorgin C (**40**)	Endophytic *Aspergillus fumigatus*	-	[21]
	Marine-derived *Aspergillus sydowi* from a driftwood sample	-	[10]
	Endophytic *Aspergillus tamari* from *Ficus carica*	-	[29]
	Marine-derived *Aspergillus* sp.	Cytotoxic activity	[34]
	Marine-derived *Pseudallescheria* sp. from the surface of driftwood	Antibacterial activity against *Staphylococcus aureus*	[23]
	Endophytic *Alternaria* sp. FL25 from *Ficus carica*	Antiphytopathogenic fungi activity	[38]
	Endophytic *Aspergillus fumigatus* from *Melia azedarach*	Plant shoot elongation inhibitory activity	[11]
*rel*-(8*R*)-9-Hydroxy-8-methoxy-18-*epi*-fumitremorgin C (**41**)	Endophytic *Aspergillus fumigatus*	-	[21]
12β-Hydroxy-13α-methoxyverruculogen TR-2 (**42**)	Endophytic *Aspergillus fumigatus* from *Melia azedarach*	Plant shoot elongation inhibitory activity	[11]
*N*-Hydroxy-6-*epi*-stephacidin (**43**)	*Aspergillus taichungensis*	-	[19]
21-Hydroxystephacidin A (**44**)	Marine-derived *Aspergillus ostianus*	-	[39]
12β-Hydroxyverruculogen TR-2 (**45**)	Endophytic *Aspergillus fumigatus* from *Melia azedarach*	-	[11]
24-Hydroxyverruculogen (**46**)	Marine sediment-derived *Penicillium brefeldianum* SD-273	-	[37]
26-Hydroxyverruculogen (**47**)	Marine sediment-derived *Penicillium brefeldianum* SD-273	-	[37]
6-Methoxyspirotryprostatin B (**48**)	Marine-derived *Aspergillus sydowi* from a driftwood sample	Weak cytotoxicity against HL-60 cells and A-549 cells	[10]
	Endophytic *Aspergillus fumigatus* from *Melia azedarach*	Inhibition on elongation of lettuce shoots	[11]
	Endophytic *Aspergillus fumigatus* from the stem of *Erythrophloeum fordii*	-	[40]
Notoamide A (**49**)	Marine-derived *Aspergillus* sp.	Moderate cytotoxicity on Hela and L1210 cells	[32]
Notoamide B (**50**)	Marine-derived *Aspergillus* sp.	Moderate cytotoxicity on Hela and L1210 cells	[32]
(-)-Notoamide B (**51**)	*Aspergillus protuberus* MF297-2	-	[32]
(+)-Notoamide B (**52**)	*Aspergillus versicolor* NRRL 35600	-	[41]
*iso*-Notoamide B (**53**)	Marine-derived *Paecilomyces variotii*	Weak cytotoxic activity	[33]
Notoamide C (**54**)	Marine-derived *Aspergillus* sp.	-	[32]
3-*epi*-Notoamide C (**55**)	Marine-derived *Aspergillus* sp.	-	[42]
Notoamide D (**56**)	Marine-derived *Aspergillus* sp.	-	[32]
Notoamide E (**57**)	*Aspergillus versicolor* NRRL 35600	-	[43]
Notoamide E_2_ (**58**)	Marine-derived *Aspergillus* sp.	-	[42]
Notoamide E_3_ (**59**)	Marine-derived *Aspergillus* sp.	-	[42]
Notoamide F (**60**)	Marine-derived *Aspergillus* sp.	-	[44]
	Marine-derived *Aspergillus ostianus*	-	[39]
Notoamide G (**61**)	Marine-derived *Aspergillus* sp.	-	[44]
Notoamide H (**62**)	Marine-derived *Aspergillus* sp.	-	[44]
Notoamide I (**63**)	Marine-derived *Aspergillus* sp.	Weak cytotoxicity on HeLa cells	[44]
Notoamide J (**64**)	Marine-derived *Aspergillus* sp.	-	[44]
Notoamide K (**65**)	Marine-derived *Aspergillus* sp.	-	[44]
Notoamide L (**66**)	Marine-derived *Aspergillus* sp.	-	[45]
Notoamide M (**67**)	Marine-derived *Aspergillus* sp.	-	[45]
Notoamide N (**68**)	Marine-derived *Aspergillus* sp.	-	[45]
Notoamide O (**69**)	Marine-derived *Aspergillus* sp.	-	[46]
Notoamide P (**70**)	Marine-derived *Aspergillus* sp.	-	[46]
Notoamide Q (**71**)	Marine-derived *Aspergillus* sp.	-	[46]
Notoamide R (**72**)	*Aspergillus ostianus*	-	[39]
	Marine-derived *Aspergillus* sp.	-	[46]
Notoamide S (**73**)	*Aspergillus amoenus*	-	[47]
Notoamide T (**74**)	Marine-derived *Aspergillus* sp.	-	[48]
6-*epi*-Notoamide T (**75**)	Marine-derived *Aspergillus* sp.	-	[48]
13-Oxofumitremorgin B (**76**)	Endophytic *Aspergillus tamari* from *Ficus carica*	-	[29]
18-Oxotryprostatin A (**77**)	Marine-derived *Aspergillus sydowi* from a driftwood sample	Weak cytotoxicity against A-549 cells	[10]
	Endophytic *Aspergillus fumigatus*	-	[21]
	Endophytic *Aspergillus fumigatus* from *Melia azedarach*	Plant growth inhibitory activity	[11]
13-Oxoverruculogen (**78**)	*Aspergillus fumigatus*	Moderate cytotoxic activity on four cancer cell lines	[8]
Piscarinine A (**79**)	*Penicillium piscarium* VKM F-691	Cytotoxic and antimicrobial activities	[49]
Piscarinine B (**80**)	*Penicillium piscarium* VKM F-691	Cytotoxic and antimicrobial activities	[49]
13-*O*-Prenyl-26-hydroxyverruculogen (**81**)	Marine sediment-derived *Penicillium brefeldianum* SD-273	Lethal activity against brine shrimp	[37]
Sclerotiamide (**82**)	*Aspergillus sclerotiorum*	Antiinsectan activity against the earworm *Helicoverpa zea*	[50]
10-*epi*-Sclerotiamide (**83**)	Deep-sea-derived *Aspergillus westerdijkiae*	-	[28]
Speramide A (**84**)	Freshwater-derived *Aspergillus ochraceus* KM007	Moderate activity against *Pseudomonas aeruginosa*	[51]
Speramide B (**85**)	Freshwater-derived *Aspergillus ochraceus* KM007	-	[51]
Spiro[5*H*,10*H*-dipyrrolo-[1,2-a:1’2’-d]pyrazine-2(3*H*),2’-[2*H*]-indol]-3’,5,10(1’*H*) trione (**86**)	Endophytic *Aspergillus fumigatus* from the stem of *Erythrophloeum fordii*	-	[40]
Spirotryprostatin A (**87**)	*Aspergillus fumigatus*	Inhibitory activity on mammalian cell cycle at G2/M phase	[12]
	Endophytic *Aspergillus fumigatus* from *Melia azedarach*	The elongation of lettuce shoots inhibitory activity	[11]
	Marine-derived *Aspergillus sydowi* from a driftwood sample	-	[10]
Spirotryprostatin B (**88**)	*Aspergillus fumigatus*	Inhibitory activity on mammalian cell cycle at G2/M phase	[12]
Spirotryprostatin C (**89**)	Holothurian-derived *Aspergillus fumigatus* from *Stichopus japonicus*	Cytotoxic activity	[8]
Spirotryprostatin D (**90**)	Holothurian-derived *Aspergillus fumigatus* from *Stichopus japonicus*	Cytotoxic activity	[8]
Spirotryprostatin E (**91**)	Holothurian-derived *Aspergillus fumigatus* from *Stichopus japonicus*	Cytotoxic activity	[8]
Spirotryprostatin Fa (**92**)	Marine-derived *Aspergillus fumigatus* from soft coral *Sinularia* sp.	Stimulating action on the growth of sprout roots of soy, buckwheat and corn	[52]
Spirotryprostatin Fb (**93**)	Plant endophytic *Penicillium brefeldianum* from the rhizome of *Pinellia ternata*	Cytotoxic activity against HepG2 and MDA-MB-231 cells	[13]
Spirotryprostatin K (**94**)	Endophytic *Aspergillus fumigatus* from the stem of *Erythrophloeum fordii*	-	[40]
(-)-Stephacidin A (**95**)	*Aspergillus amoenus* (formerly *A. versicolor*) NRRL 35600	-	[41]
(+)-Stephacidin A (**96**)	*Aspergillus protuberus* MF297-2	-	[32]
6-*epi*-Stephacidin A (**97**)	*Aspergillus taichungensis*	-	[19]
Stephacidin B (**98**)	*Aspergillus ochraceus*	Cytotoxic activity	[14]
Taichunamide C (**99**)	*Aspergillus taichungensis* (IBT 19404)	-	[53]
Taichunamide E (**100**)	*Aspergillus taichungensis* (IBT 19404)	-	[53]
Taichunamide F (**101**)	*Aspergillus taichungensis* (IBT 19404)	-	[53]
Taichunamide G (**102**)	*Aspergillus taichungensis* (IBT 19404)	-	[53]
Tryprostatin A (**103**)	Endophytic *Aspergillus fumigatus* from *Melia azedarach*	Inhibitory activities on elongation of lettuce shoots, and on multidrug-resistance protein	[11,16]
	Endophytic *Aspergillus tamari* from *Ficus carica*	-	[29]
Tryprostatin B (**104**)	Endophytic *Aspergillus fumigatus*	-	[21]
	Endophytic *Aspergillus tamari* from *Ficus carica*	-	[29]
	-	Inhibitory activity on mammalian cell-cycle	[17]
Verruculogen (**105**)	Endophytic *Aspergillus fumigatus* from *Melia azedarach*	The elongation of lettuce shoots inhibitory activity	[11]
	Endophytic *Aspergillus tamari* from *Ficus carica*	-	[29]
	Marine sediment-derived fungus *Penicillium brefeldianum* SD-273	-	[37]
Verruculogen TR-2 = TR-2 (**106**)	Endophytic *Aspergillus fumigatus* from *Melia azedarach*	Inhibitory activity on elongation of lettuce shoots	[11]
	Marine sediment-derived *Penicillium brefeldianum* SD-273	-	[37]
Versicamide A (**107**)	Marine-derived *Aspergillus versicolor*	-	[36]
Versicamide B (**108**)	Marine-derived *Aspergillus versicolor*	-	[36]
Versicamide C (**109**)	Marine-derived *Aspergillus versicolor*	-	[36]
Versicamide D (**110**)	Marine-derived *Aspergillus versicolor*	-	[36]
Versicamide E (**111**)	Marine-derived *Aspergillus versicolor*	-	[36]
Versicamide F (**112**)	Marine-derived *Aspergillus versicolor*	-	[36]
Versicamide G (**113**)	Marine-derived *Aspergillus versicolor*	-	[36]
(−)-Versicolamide B (**114**)	*Aspergillus* sp.	-	[45]
(+)-Versicolamide B (**115**)	*Aspergillus versicolor* NRRL 35600	-	[41]
(−)-Versicolamide C (**116**)	*Aspergillus taichungensis*	-	[19]

**Table 2:** Fungal tryptophan-tryptophan cyclodipeptide analogs and their biological activities.

Name	Fungus and its Origin	Biological Activity	Ref.
Amauromine = Nigriforine (**117**)	*Amauroascus* sp.	Hypotensive vasodilating activity	[54]
	*Penicillium nigricans*	-	[55]
	*Auxarthron reticulatum*	Selective cannabinoid CB1 receptor antagonist	[56]
Amauromine B (**118**)	*Aspergillus terreus* 3.05358	Inhibitory activity on α-glucosidase	[68]
Cyclo(l-Trp–l-Trp) (**119**)	Endophytic *Aspergillus niger* from the liverwort *Heteroscyphus tener*	-	[69]
Epiamauromine (**120**)	*Aspergillus ochraceus*	Moderate reduction in weight gain activity against the corn earworm	[57]
Fellutanine A (**121**)	*Penicillium fellutanum*	-	[58]
	Marine sponge-derived *Neosartorya glabra* KUFA 0702	-	[70]
Fellutanine A 2’*S*,3’*S*-epoxide (**122**)	Marine sponge-derived *Neosartorya glabra* KUFA 0702	-	[70]
Fellutanine B (**123**)	*Penicillium fellutanum*	-	[58]
Fellutanine C (**124**)	*Penicillium fellutanum*	-	[58]
Fellutanine D (**125**)	*Penicillium fellutanum*	Cytotoxic activity	[58]
Gypsetin (**126**)	*Nannizzia gypsea* var. *incurvata*	Inhibitory activity on acyl-CoA:cholesterol acyltransferase	[71]
*N*-Methylepiamauromine (**127**)	*Aspergillus ochraceus*	Moderate reduction in weight gain activity against the corn earworm	[57]
Novoamauromine (**128**)	*Aspergillus novofumigatus*	Inhibitory activity on the cell proliferation of A549, Hela, and LNCap cells	[59]
Okaramine A (**129**)	*Penicillium simplicissimum* AK-40	Insecticidal activity	[62]
Okaramine B (**130**)	*Penicillium simplicissimum* AK-40	Insecticidal activity	[62]
Okaramine C (**131**)	*Penicillium simplicissimum*	-	[65]
	*Penicillium simplicissimum*	Oral insecticide activity against silkworms	[72]
Okaramine D (**132**)	*Penicillium simplicissimum*	Insecticidal activity	[63]
Okaramine E (**133**)	*Penicillium simplicissimum*	-	[63]
Okaramine F (**134**)	*Penicillium simplicissimum*	-	[63]
Okaramine G (**135**)	*Penicillium simplicissimum*	Insecticidal activity	[73]
Okaramine H (**136**)	*Aspergillus aculeatus*	-	[60]
Okaramine I (**137**)	*Aspergillus aculeatus*	-	[60]
Okaramine J (**138**)	*Penicillium simplicissimum*	-	[64]
Okaramine K (**139**)	*Penicillium simplicissimum*	-	[64]
Okaramine L (**140**)	*Penicillium simplicissimum*	-	[64]
Okaramine M (**141**)	*Penicillium simplicissimum*	-	[64]
Okaramine N (**142**)	*Penicillium simplicissimum*	-	[65]
Okaramine O (**143**)	*Penicillium simplicissimum*	-	[65]
Okaramine P (**144**)	*Penicillium simplicissimum*	-	[65]
Okaramine Q (**145**)	*Penicillium simplicissimum*	-	[65]
Okaramine R (**146**)	*Penicillium simplicissimum*	-	[65]
Okaramine S (**147**)	*Aspergillus taichungensis* ZHN-7-07	Cytotoxic activity against HL-60 cells with IC_50_ value of 0.78 μM	[61]
Okaramine T (**148**)	*Aspergillus taichungensis* ZHN-7-07	-	[61]
Okaramine U (**149**)	*Aspergillus taichungensis* ZHN-7-07	-	[61]

Note: IC_50_, median inhibitory concentration.

**Table 3:** Fungal tryptophan-Xaa cyclodipeptide analogs and their biological activities.

Name	Fungus and its Origin	Biological Activity	Ref.
Acyl aszonalenin (**150**)	*Aspergillus flavipes*	Substance P inhibitory activity	[88]
Alkaloid E-7 (**151**)	Halotolerant *Aspergillus variecolor* from sediments collected in the Jilantai salt field of China	Weak radical scavenging activity against DPPH	[87]
	Mangrove-derived *Eurotium rubrum* from *Hibiscus tiliaceus*	Cytotoxic activity	[89]
	Marine-derived *Eurotium rubrum* MPUC136	Inhibitory activity against melanin synthesis	[90]
Arestrictin A (**152**)	*Aspergillus restrictus*	-	[91]
Arestrictin B (**153**)	*Aspergillus restrictus*	-	[91]
	*Aspergillus penicilloides*	-	[91]
Asperazine (**154**)	Marine-derived *Aspergillus niger*	Cytotoxic activity	[92]
	Endophytic *Aspergillus niger* from the liverwort *Heteroscyphus tener*	Weak cytotoxic activity	[69]
	Endophytic *Aspergillus* sp. KJ-9 from *Melia azedarach*	Antifungal and antibacterial activity	[93]
	Plant fungal pathogen *Pestalotiopsis theae*	Inhibitory effect on HIV-1 replication in C8166 cells	[94]
Asperazine A (**155**)	Endophytic *Aspergillus niger* from the liverwort *Heteroscyphus tener*	Weak cytotoxic activity	[69]
Aspertryptanthrin A (**156**)	Endophytic *Aspergillus* sp. from the stem bark of *Melia azedarach*	-	[74]
Aspertryptanthrin B (**157**)	Endophytic *Aspergillus* sp. from the stem bark of *Melia azedarach*	-	[74]
Aspertryptanthrin C (**158**)	Endophytic *Aspergillus* sp. from the stem bark of *Melia azedarach*	-	[74]
Benzodiazepinedione (**159**)	*Aspergillus flavipes*	-	[88]
Brevicompanine A (**160**)	*Penicillium brevicompactum*	Acceleration of the root growth of the lettuce seedlings	[95]
Brevicompanine B (**161**)	*Penicillium brevicompactum*	Acceleration of the root growth of the lettuce seedlings	[95]
	*Aspergillus janus*	Inhibitory activity against the malaria parasite *Plasmodium falciparum* 3D7	[96]
*allo*-Brevicompanine B (**162**)	Deep ocean sediment derived fungus *Penicillium* sp.	-	[77]
Brevicompanine C (**163**)	*Penicillium brevi-compactum*	Acceleration of the root growth of the lettuce seedlings	[75]
Brevicompanine D (**164**)	Deep ocean sediment derived *Penicillium* sp.	-	[77]
Brevicompanine E (**165**)	Deep ocean sediment derived *Penicillium* sp.	Inhibitory activity on lipopolysaccharide-induced nitric oxide production in BV2 microglial cells	[77,78]
Brevicompanine F (**166**)	Deep ocean sediment derived *Penicillium* sp.	-	[77]
Brevicompanine G (**167**)	Deep ocean sediment derived *Penicillium* sp.	-	[77]
Brevicompanine H (**168**)	Deep ocean sediment derived *Penicillium* sp.	Inhibitory activity on lipopolysaccharide-induced nitric oxide production in BV2 microglial cells	[77]
Citreoindole (**169**)	*Penicillium citreoviride*	Cytotoxicity against HeLa cells	[97]
Cristatin A (**170**)	*Aspergillus penicilloides*	-	[91]
Cristatumin A (**171**)	Mangrove-derived endophytic *Eurotium cristatum* EN-220	Antibacterial activity against *Escherichia coli* and *Staphyloccocus aureus*	[98]
Cristatumin B (**172**)	Mangrove-derived endophytic *Eurotium cristatum* EN-220	Moderate lethal activity against brine shrimp	[98]
Cristatumin C (**173**)	Mangrove-derived endophytic *Eurotium cristatum* EN-220	-	[98]
Cristatumin E (**174**)	Algal-derived *Eurotium herbariorum* HT-2	Cytotoxic activity on K562 tumor cell line and antibacterial activity on *Enterobacter aerogenes* and *Escherichia coli*	[99]
Cristatumin F (**175**)	*Eurotium cristatum* isolated from Fuzhuan brick tea	Modest radical scavenging activity against DPPH radicals, and marginal attenuation of 3T3L1 pre-adipocytes	[100]
Cryptoechinuline C (**176**)	Marine-derived *Eurotium rubrum*	-	[84]
Cryptoechinuline D (**177**)	Mangrove rhizosphere soil-derived *Aspergillus effuses* H1-1	Cytotoxic activity	[101]
	Mangrove-derived *Eurotium rubrum* from the inner tissue of stems of *Hibiscus tiliaceus*	Radical scavenging activity against DPPH	[102]
Cryptoechinuline G (**178**)	Halotolerant *Aspergillus variecolor* from sediments collected in the Jilantai salt field of China	Weak radical scavenging activity against 1,1‘-diphenyl-1-picryhydrazyl (DPPH)	[87]
	Marine-derived *Eurotium rubrum* MPUC136	Inhibitory activityagainst melanin synthesis	[90]
	Mangrove-derived *Eurotium rubrum* from *Hibiscus tiliaceus*	-	[89]
Cyclo(l-Trp–l-Ala) (**179**)	Marine-derived *Eurotium rubrum*	Weak antiviral effects	[84]
	Marine-derived *Aspergillus* sp.	-	[103]
	*Eurotium rubrum* MA-150 obtained from mangrove-derived rhizospheric soil	Modest lethal activity on brine shrimp	[104]
Cyclo(Trp–Gly) (**180**)	Thermophilic *Talaromyces thermophilus* YM3-4 collected in Tengchong hot spring, Yunnan of China	-	[105]
Cyclo(l-Trp–dehydro-His) (**181**)	Endophytic *Penicillium* sp. HS-3 from the stems of *Huperzia serrata*	-	[106]
Cyclo(l-Trp–d-Ile) (**182**)	*Penicillium brevi-compactum*	Acceleration of root growth of lettuce seedlings	[75]
Cyclo(l-Trp–d-Leu) (**183**)	*Penicillium brevi-compactum*	Acceleration of root growth of lettuce seedlings	[75]
Cyclo(*N*-methyl-Trp–Leu) (**184**)	Endophytic *Aspergillus tamari* from *Ficus carica*	-	[29]
Cyclo(l-Trp-d-*N*-methyl-Leu) (**185**)	*Aspergillus flavus*	-	[107]
Cyclo(l-Trp–d-Phe) (**186**)	Endophytic *Aspergillus niger* from the liverwort *Heteroscyphus tener*	-	[69]
Cyclo(l-Trp–l-Phe) (**187**)	*Aspergillus syndowi*	-	[108]
	*Penicillium* sp.	Acceleration of root growth of lettuce seedlings	[76]
Cyclo(Trp–Tyr) (**188**)	Terrestrial *Aspergillus oryzae*	-	[109]
Cyclo(l-*N*-isopropyl-Trp–l-Val) (**189**)	An unidentified marine derived fungus M-3 from laver (*Porphyra yezoensis*)	Antifungal activity against the rice pathogen *Pyricularia oryzae* with MIC 0.36 μM	[110]
Cycloechinulin (**190**)	*Aspergillus ochraceus*	Insecticidal activity against the lepidopteran crop pest *Helicoverpa zea*	[57]
*ent*-Cycloechinulin (**191**)	*Aspergillus novofumigatus* CBS117520	Antifungal activity against *Aspergillus fumigatus*, *A. Niger*, *Candida albicans*, and *Cryptococcus neoformans*	[59]
Dehydroechinulin (**192**)	*Eurotium cristatum* isolated from Fuzhuan brick tea	-	[100]
	Mangrove-derived *Eurotium rubrum* from the inner tissue of stems of *Hibiscus tiliaceus*	-	[102]
	Mangrove rhizosphere soil-derived *Eurotium rubrum* MA-150	Lethal activity on brine shrimp	[104]
	Lichen-derived *Eurotium* sp. No. 17-11-8-1 from *Cladina grisea* collected in Changbaishan Mountain of China	-	[111]
Dehydrovariecolorin L (**193**)	Mangrove-derived *Eurotium rubrum* from the inner tissue of stems of *Hibiscus tiliaceus*	-	[102]
12-Demethyl-12-oxo-eurotechinulin B (**194**)	Mangrove-derived *Eurotium rubrum* from *Hibiscus tiliaceus*	Cytotoxic activity	[89]
Dichotocejpin B (**195**)	*Dichotomomyces cejpii* FS110	-	[112]
Dichotocejpin C (**196**)	*Dichotomomyces cejpii* FS110	-	[112]
	Marine-derived *Dichotomomyces* sp. L-8	-	[113]
Didehydroechinulin = Didehydroechinulin B (**197**)	Deep ocean sediment-derived *Penicillium griseofulvum*	-	[86]
	Mangrove rhizosphere soil-derived *Aspergillus effuses* H1-1	Cytotoxic activity	[101]
Dihydrocryptoechinulin D (**198**)	Mangrove rhizosphere soil-derived *Aspergillus effuses* H1-1	Cytotoxic activity against P388 cells	[114]
Dihydroneochinulin B (**199**)	Mangrove rhizosphere soil-derived *Aspergillus effuses* H1-1	Weak cytotoxic activity against BEL-7402 and A-549 cell lines	[101]
3,12-Dihydroroquefortine (**200**)	Permafrost sediment derived *Penicillium aurantiogrieum*	-	[115]
7,9-Dihydroxy-3-(1*H*-indol--ylmethyl)-8-methoxy-2,3,11,11a-tetrahydro-6*H*-pyrazino[1,2-*b*]isoquinoline-1,4-dione (**201**)	Terrestrial *Aspergillus oryzae*	-	[109]
Dihydroxyisoechinulin A (**202**)	Halotolerant *Aspergillus variecolor* from sediments collected in the Jilantai salt field of China	Weak radical scavenging activity against DPPH	[87]
	Mangrove-derived *Eurotium rubrum* from the inner tissue of stems of *Hibiscus tiliaceus*	-	[102]
	Mangrove rhizosphere soil-derived *Eurotium rubrum* MA-150	Modest brine shrimp lethal and antibacterial activities	[104]
11,14-Dihydroxylneoechinulin E (**203**)	Mushroom *Psilocybe merdaria* from suburban district of Haikou of China	-	[116]
Dipodazine (**204**)	*Penicillium dipodomyis*	-	[117]
Ditryptophenaline (**205**)	*Aspergillus flavus*	-	[118]
	*Aspergillus flavus*	Weak substance-P inhibitor activity	[119]
	Marine-derived *Aspergillus flavus* C-F-3	-	[120]
	Terrestrial *Aspergillus oryzae*	-	[109]
Echinulin (**206**)	Halotolerant *Aspergillus variecolor* from sediments collected in the Jilantai salt field of China	-	[87]
	*Aspergillus chevalieri* in rabbits	Toxic activity to rabbits by producing a significant degree of damage to lung and liver	[79]
	Soil-derived *Chaetomium globosum*	-	[121]
	*Eurotium cristatum* isolated from Fuzhuan brick tea	-	[100]
	Mangrove-derived *Eurotium rubrum* from the inner tissue of stems of *Hibiscus tiliaceus*	-	[102]
	Marine-derived *Eurotium rubrum*	Weak antiviral effect	[84]
	Marine-derived *Eurotium rubrum* MPUC136	Inhibitory activity against melanin synthesis	[90]
	Deep ocean sediment-derived *Penicillium griseofulvum*	-	[86]
	Mangrove rhizosphere soil-derived *Eurotium rubrum* MA-150	Modest lethal activity on brine shrimp	[104]
Effusin A (**207**)	Mangrove rhizosphere soil-derived *Aspergillus effuses* H1-1	-	[114]
Epoxyisoechinulin A (**208**)	Marine-derived *Aspergillus ruber* 1017 from a crinoid *Himerometra magnipinna*	-	[122]
Eurocristatine (**209**)	Sponge-associated *Eurotium cristatum* KUFC 7356	-	[123]
Eurotechinulin B (**210**)	Mangrove-derived *Eurotium rubrum* from *Hibiscus tiliaceus*	-	[89]
Fructigenine A = Rugulosuvine B (**211**)	*Penicillium fructigenum*	Inhibitory activity on the growth of *Avena coleoptiles* and L-5178Y cells	[80]
	*Penicillium rugulosum*	Moderate cytotoxic activity	[81]
Fructigenine B = Verrucofortine (**212**)	Deep ocean sediment derived *Penicillium* sp.	-	[77]
	*Penicillium verrucosum* var. c*yclopium*	-	[124]
	*Penicillium fructigenum*	-	[80]
Gliocladin C (**213**)	*Gliocladium roseum* PS-N132	Significant cytotoxicity against murine P388 lymphocytic leukemia cells	[125]
Glioperazine C (**214**)	*Bionectra byssicola* F120	-	[126]
Haenamindole (**215**)	Marine-derived *Penicillium* sp. KCB12F005	-	[127]
3-((1-Hydroxy-3-(2-methylbut- 3-en-2-yl)-2-oxoindilin- 3-yl)methyl)-1-methyl-3,4-dihydrobenzo[1,4] diazepine-2,5-dione (**216**)	Sponge-derived *Aspergillus* sp. from the Mediterranean sponge *Tethya auranthium*	Antibacteria activity on a few marine-derived *Vibrio* species	[128]
16-Hydroxyroquefortine C (**217**)	Endophytic *Penicillium* sp. HS-3 from the stems of *Huperzia serrata*	-	[106]
14-Hydroxyterezine D (**218**)	Marine-derived *Aspergillus sydowi* from a driftwood sample	Weak cytotoxic activity against A-549 cells	[10]
(*E*)-3-(1*H*-Imidazole-4-yimethylene)-6-(1*H*-indl-3-ylmethyl)-2,5-piperazinediol (**219**)	Antarctic soil-derived *Penicillium* sp. SCSIO 05705	-	[129]
3-[(1*H*-Indol-3-yl)methyl]-6-benzylpiperazine-2,5-dione (**220**)	Marine-derived *Aspergillus flavus* C-F-3	-	[120]
(3*S*, 11a*S*)-3- [(1*H*-Indol-3-yl) methyl]-7,9- dihydroxy-8-methoxy- 2,3,11,11a-tetrahydro- 6*H*- pyrazino [1,2-*b*] isoquinoline-1,4-dione (**221**)	Algicolous *Aspergillus flavus*	Weak cytotoxicity against HL-60 cells	[130]
Isoechinulin A (**222**)	Halotolerant *Aspergillus variecolor* from sediments collected in the Jilantai salt field of China	Weak radical scavenging activity against DPPH	[87]
	Mangrove-derived *Eurotium rubrum* from the inner tissue of stems of *Hibiscus tiliaceus*	-	[102]
	Mangrove rhizosphere soil-derived *Eurotium rubrum* MA-150	Modest lethal activity on brine shrimp	[104]
	Lichen-derived *Eurotium* sp. No. 17-11-8-1 from *Cladina grisea* collected in Changbaishan Mountain of China	-	[111]
	Marine-derived *Eurotium rubrum*	Inhibitory activity against influenza A/WSN/33 virus	[84]
Isoechinulin B (**223**)	Halotolerant *Aspergillus variecolor* from sediments collected in the Jilantai salt field of China	Weak radical scavenging activity against DPPH	[87]
	Marine-derived *Eurotium rubrum* MPUC136	Inhibitory activity against melanin synthesis	[90]
	Marine-derived *Eurotium rubrum*	-	[84]
	Mangrove-derived *Eurotium rubrum* from *Hibiscus tiliaceus*	-	[89]
	Deep ocean sediment-derived *Penicillium griseofulvum*	-	[86]
Isoechinulin C (**224**)	Marine-derived *Eurotium rubrum* MPUC136	-	[90]
Isoechinulin D (**225**)	Marine-derived *Eurotium rubrum* MPUC136	-	[90]
Isopenilline A (**226**)	Antarctic soil-derived *Penicillium* sp. SCSIO 05705	-	[129]
7-Isopentenylcryptoechinuline D (**227**)	Mangrove-derived *Eurotium rubrum* from *Hibiscus tiliaceus*	-	[89]
Leptosin S (**228**)	*Leptosphaeria* sp. from a marine alga	Cytotoxicity on P388 cells	[131]
Lumpidin (**229**)	*Penicillium nordicum*	-	[132]
3-Methyl-6-[[(1-(3-methyl-2-butenyl)-1*H*-indol-3-yl)methyl)-2,5-piperazinediione (**230**)	Marine-derived *Eurotium rubrum*	Weak antiviral effect	[84]
7-*O*-Methylvariecolortide A (**231**)	Mangrove derived endophytic *Eurotium rubrum* from the inner tissue of the stems of *Hibiscus tilliaceus*	-	[133]
	Lichen-derived *Eurotium* sp. No. 17-11-8-1 from *Cladina grisea* collected in Changbaishan Mountain of China	Inhibitory activity on caspase-3	[111]
(+)-(*R*)-7-*O*-Methylvariecolortide A (**232**)	Lichen-derived *Eurotium* sp. No. 17-11-8-1 from *Cladina grisea* collected in Changbaishan Mountain of China	-	[111]
(−)-(*S*)-7-*O*-Methylvariecolortide A (**233**)	Lichen-derived *Eurotium* sp. No. 17-11-8-1 from *Cladina grisea* collected in Changbaishan Mountain of China	-	[111]
Neoechinulin A (**234**)	*Aspergillus* spp.	Scavenging, neurotrophic factor-like and antiapoptotic activities	[82]
	Halotolerant *Aspergillus variecolor* from sediments collected in the Jilantai salt field of China	Weak radical scavenging activity against DPPH	[87]
	Marine-derived *Aspergillus* sp.	Ultraviolet-A (320-390 nm) protecting activity with IC_50_ value of 170 μM.	[103]
	Marine mudflat sediment derived *Chaetomium cristatum* collected at Suncheon Bay of Korea	Radical-scavenging activity against DPPH with IC_50_ value of 24 μM	[134]
	*Eurotium cristatum* from Fuzhuan brick tea	-	[100]
	Mangrove-derived *Eurotium rubrum* from the inner tissue of stems of *Hibiscus tiliaceus*	-	[102]
	Mangrove rhizosphere soil-derived *Eurotium rubrum* MA-150	Modest brine shrimp lethal activity	[104]
	Marine-derived *Eurotium rubrum* MPUC136	-	[90]
	Lichen-derived *Eurotium* sp. No. 17-11-8-1 from *Cladina grisea* collected in Changbaishan Mountain of China	-	[111]
	Deep ocean sediment-derived *Penicillium griseofulvum*	-	[86]
	Mushroom *Psilocybe merdaria* from suburban district of Haikou of China	-	[116]
	Marine-derived *Eurotium rubrum*	-	[84]
Neoechinulin B (**235**)	Mangrove rhizosphere soil-derived *Aspergillus effuses* H1-1	-	[101]
	Halotolerant *Aspergillus variecolor* from sediments collected in the Jilantai salt field of China	Weak radical scavenging activity against DPPH	[87]
	Marine-derived *Eurotium rubrum*	Inhibition against H_1_N_1_ virus infected in MDCK cells, and a panel of influenza virus strains	[84]
	Deep ocean sediment-derived *Penicillium griseofulvum*	-	[86]
Neoechinulin C (**236**)	Marine-derived *Eurotium rubrum*	Inhibitory activity against influenza A/WSN/33 virus	[84]
Neoechinulin D (**237**)	*Aspergillus amstelodami*	-	[135]
Neoechinulin E (**238**)	Mangrove-derived *Eurotium rubrum* from the inner tissue of stems of *Hibiscus tiliaceus*	DPPH radical scavenging activity	[102]
	Mangrove rhizosphere soil-derived *Eurotium rubrum* MA-150	Lethal activity on brine shrimp	[104]
Neosartin A (**239**)	Marine-derived *Neosartorya pseudofischeri* from the inner tissue of starfish *Acanthaster planci*	-	[136]
Neosartin B (**240**)	Marine-derived *Neosartorya pseudofischeri* from the inner tissue of starfish *Acanthaster planci*	-	[136]
Oidioperazine B (**241**)	Antarctic psychrophilic fungus *Oidiodendron truncatum*	-	[137]
Oidioperazine C (**242**)	Antarctic psychrophilic fungus *Oidiodendron truncatum*	-	[137]
Oidioperazine D (**243**)	Antarctic psychrophilic fungus *Oidiodendron truncatum*	-	[137]
Penilline A (**244**)	Antarctic soil-derived *Penicillium* sp. SCSIO 05705	-	[129]
Penilline B (**245**)	Antarctic soil-derived *Penicillium* sp. SCSIO 05705	-	[129]
Penilloid A (**246**)	Antarctic soil-derived *Penicillium* sp. SCSIO 05705	-	[129]
Pestalazine A (**247**)	Plant pathogen *Pestalotiopsis theae*	Inhibitory activity on HIV-1 replication in C8166 cells	[94]
Pestalazine B (**248**)	Plant pathogen *Pestalotiopsis theae*	-	[94]
1’-(2-Phenyl-ethylene)- ditryptophenaline (**249**)	*Aspergillus flavus*	Weak substance-P inhibitor activity	[119]
Polanrazine A = Cyclo(l-Trp–l-Val) (**250**)	Plant pathogen *Phoma lingam*	Phytotoxic activity	[83]
Polanranine E (**251**)	Plant pathogen *Phoma lingam*	Moderate and selective phytotoxicity by causing necrotic and chlorotic lesions	[138]
Polanranine F (**252**)	Plant pathogen *Phoma lingam*	-	[138]
Preechinulin (**253**)	Halotolerant *Aspergillus variecolor* from sediments collected in the Jilantai salt field of China	-	[87]
	Mangrove-derived *Eurotium rubrum* from the inner tissue of stems of *Hibiscus tiliaceus*	-	[102]
	Marine-derived *Eurotium rubrum*	Weak antiviral effect	[84]
	Deep ocean sediment-derived *Penicillium griseofulvum*	-	[86]
Protubonine A (**254**)	Marine-derived *Aspergillus* sp. SF-5044 from an intertidal sediment sample	-	[139]
Protubonine B (**255**)	Marine-derived *Aspergillus* sp. SF-5044 from an intertidal sediment sample	-	[139]
Rhinocladin A (**256**)	Endophytic *Rhinocladiella* sp. lgt-3 from *Tripterygium wilfordii*	Weak inhibitory activity on monoamine oxidase	[140]
Rhinocladin B (**257**)	Endophytic *Rhinocladiella* sp. lgt-3 from *Tripterygium wilfordii*	Weak inhibitory activity on monoamine oxidase	[140]
Roquefortine C = Roquefortine (**258**)	Permafrost sediment derived *Penicillium aurantiogriseum*	-	[115]
	*Penicillium roqueforti* from soil	-	[141]
	Endophytic *Penicillium* sp. HS-3 from the stems of *Huperzia serrata*	-	[106]
Roquefortine E (**259**)	*Gymnoascus reessii*	Weak cytotoxic activity to mammalian cells	[142]
Roquefortine F (**260**)	Marine-derived *Penicillium* sp.	-	[143]
Roquefortine G (**261**)	Marine-derived *Penicillium* sp.	-	[143]
Roquefortine H (**262**)	Deep ocean sediment-derived *Penicillium* sp.	-	[144]
Roquefortine I (**263**)	Deep ocean sediment-derived *Penicillium* sp.	-	[144]
Rubrumazine A (**264**)	Mangrove rhizosphere soil-derived *Eurotium rubrum* MA-150	Modest brine shrimp lethal activity	[104]
Rubrumazine B (**265**)	Mangrove rhizosphere soil-derived *Eurotium rubrum* MA-150	Brine shrimp lethal activity	[104]
Rubrumazine C (**266**)	Mangrove rhizosphere soil-derived *Eurotium rubrum* MA-150	Modest brine shrimp lethal activity	[104]
Rubrumline A (**267**)	Marine-derived *Eurotium rubrum*	-	[84]
	Marine-derived *Eurotium rubrum* MPUC136	-	[90]
Rubrumline B (**268**)	Marine-derived *Eurotium rubrum*	-	[84]
Rubrumline C (**269**)	Marine-derived *Eurotium rubrum*	-	[84]
Rubrumline D (**270**)	Marine-derived *Eurotium rubrum*	Inhibitory activity against influenza A/WSN/33 virus	[84]
	Marine-derived *Eurotium rubrum* MPUC136	-	[90]
Rubrumline E (**271**)	Marine-derived *Eurotium rubrum*	-	[84]
Rubrumline F (**272**)	Marine-derived *Eurotium rubrum*	Weak antiviral effect	[84]
Rubrumline G (**273**)	Marine-derived *Eurotium rubrum*	Weak antiviral effect	[84]
Rubrumline H (**274**)	Marine-derived *Eurotium rubrum*	-	[84]
Rubrumline I (**275**)	Marine-derived *Eurotium rubrum*	-	[84]
Rubrumline J (**276**)	Marine-derived *Eurotium rubrum*	Weak antiviral effect	[84]
Rubrumline K (**277**)	Marine-derived *Eurotium rubrum*	-	[84]
Rubrumline L (**278**)	Marine-derived *Eurotium rubrum*	-	[84]
Rubrumline M (**279**)	Marine-derived *Eurotium rubrum*	Weak antiviral effect	[84]
Rubrumline N (**280**)	Marine-derived *Eurotium rubrum*	Weak antiviral effect	[84]
Rubrumline O (**281**)	Marine-derived *Eurotium rubrum*	Weak antiviral effect	[84]
Rugulosuvine A (**282**)	*Penicillium rugulosum*	Moderate cytotoxic activity	[81]
SF5280-415 (**283**)	Marine-derived *Aspergillus* sp. SF-5280	-	[145]
Talathermophilin A (**284**)	Thermophilic *Talaromyces thermophilus* YM1-3	Nematicidal toxicity	[85]
	Thermophilic *Talaromyces thermophilus* YM3-4 collected in Tengchong hot spring, Yunnan of China	-	[105]
Talathermophilin B (**285**)	Thermophilic *Talaromyces thermophilus* YM1-3	Nematicidal toxicity	[85]
	Thermophilic *Talaromyces thermophilus* YM3-4 collected in Tengchong hot spring, Yunnan of China	-	[105]
Talathermophilin C (**286**)	Thermophilic *Talaromyces thermophilus* YM3-4 collected in Tengchong hot spring, Yunnan of China	-	[105]
Talathermophilin D (**287**)	Thermophilic *Talaromyces thermophilus* YM3-4 collected in Tengchong hot spring, Yunnan of China	-	[105]
Talathermophilin E (**288**)	Thermophilic *Talaromyces thermophilus* YM3-4 collected in Tengchong hot spring, Yunnan of China	-	[105]
Tardioxopiperazine A (**289**)	Halotolerant *Aspergillus variecolor* from sediments collected in the Jilantai salt field of China	-	[87]
	Mangrove rhizosphere soil-derived *Eurotium rubrum* MA-150	Modest lethal activity on brine shrimp	[104]
	Lichen-derived *Eurotium* sp. No. 17-11-8-1 from *Cladina grisea* collected in Changbaishan Mountain of China	-	[111]
	*Microascus tardifaciens*	Moderate inhibition on con A and LPS mediated T cell proliferation	[146]
	Deep ocean sediment-derived *Penicillium griseofulvum*	-	[86]
Tardioxopiperaine B (**290**)	Halotolerant *Aspergillus variecolor* from sediments collected in the Jilantai salt field of China	-	[87]
	*Microascus tardifaciens*	Weak inhibition on con A and LPS mediated T cell proliferation	[146]
Terezine D (**291**)	Marine-derived *Aspergillus sydowi* from a driftwood sample	-	[10]
Tryhistatin (**292**)	Endophytic fungus *Penicillium* sp. HS-3 from the stems of *Huperzia serrata*	-	[106]
Variecolorin A (**293**)	Halotolerant *Aspergillus variecolor* from sediments collected in the Jilantai salt field of China	Weak radical scavenging activity against DPPH	[87]
Variecolorin B (**294**)	Halotolerant *Aspergillus variecolor* from sediments collected in the Jilantai salt field of China	Weak radical scavenging activity against DPPH	[87]
Variecolorin C (**295**)	Halotolerant *Aspergillus variecolor* from sediments collected in the Jilantai salt field of China	Weak radical scavenging activity against DPPH	[87]
Variecolorin D (**296**)	Halotolerant *Aspergillus variecolor* from sediments collected in the Jilantai salt field of China	Weak radical scavenging activity against DPPH	[87]
Variecolorin E (**297**)	Halotolerant *Aspergillus variecolor* from sediments collected in the Jilantai salt field of China	Weak radical scavenging activity against DPPH	[87]
	*Eurotium rubrum* MA-150 obtained from mangrove-derived rhizospheric soil	Modest lethal activity on brine shrimp	[104]
Variecolorin F (**298**)	Halotolerant *Aspergillus variecolor* from sediments collected in the Jilantai salt field of China	Weak radical scavenging activity against DPPH	[87]
Variecolorin G (**299**)	Halotolerant *Aspergillus variecolor* from sediments collected in the Jilantai salt field of China	Weak radical scavenging activity against DPPH	[87]
	Mangrove-derived *Eurotium rubrum* from *Hibiscus tiliaceus*	Cytotoxic activity	[89]
	*Eurotium rubrum* MA-150 obtained from mangrove-derived rhizospheric soil	Modest lethal activity on brine shrimp	[104]
Variecolorin H (**300**)	Halotolerant *Aspergillus variecolor* from sediments collected in the Jilantai salt field of China	Weak radical scavenging activity against DPPH	[87]
	Deep ocean sediment-derived fungus *Penicillium griseofulvum*	-	[86]
Variecolorin I (**301**)	Halotolerant *Aspergillus variecolor* from sediments collected in the Jilantai salt field of China	Weak radical scavenging activity against DPPH	[87]
Variecolorin J (**302**)	Halotolerant *Aspergillus variecolor* from sediments collected in the Jilantai salt field of China	Weak radical scavenging activity against DPPH	[87]
	Mangrove-derived *Eurotium rubrum* from *Hibiscus tiliaceus*	-	[89]
Variecolorin K (**303**)	Halotolerant *Aspergillus variecolor* from sediments collected in the Jilantai salt field of China	Weak radical scavenging activity against DPPH	[87]
Variecolorin L (**304**)	Halotolerant *Aspergillus variecolor* from sediments collected in the Jilantai salt field of China	-	[87]
	Mangrove-derived *Eurotium rubrum* from the inner tissue of stems of *Hibiscus tiliaceus*	-	[102]
	Mangrove rhizosphere soil-derived *Eurotium rubrum* MA-150	Modest lethal activity on brine shrimp	[104]
Variecolorin M (**305**)	Deep ocean sediment-derived *Penicillium griseofulvum*	Weak radical scavenging activity against DPPH	[86]
Variecolorin N (**306**)	Deep ocean sediment-derived *Penicillium griseofulvum*	Weak radical scavenging activity against DPPH	[86]
Variecolorin O (**307**)	*Eurotium cristatum* isolated from Fuzhuan brick tea	-	[100]
	Deep ocean sediment-derived *Penicillium griseofulvum*	Weak radical scavenging activity against DPPH	[86]
Variecolortide A (**308**)	Halotolerant fungus *Aspergillus variecolor* B-17	Weak cytotoxic and antioxidant activities	[147]
Variecolortide B (**309**)	Halotolerant *Aspergillus variecolor* B-17	Weak cytotoxic and antioxidant activities	[147]
(−)-(*S*)-Variecolortide B (**310**)	Lichen-derived *Eurotium* sp. No. 17-11-8-1 from *Cladina grisea* collected in Changbaishan Mountain of China	-	[111]
(+)-(*R*)-Variecolortide B (**311**)	Lichen-derived *Eurotium* sp. No. 17-11-8-1 from *Cladina grisea* collected in Changbaishan Mountain of China	-	[111]
Variecolortide C (**312**)	Halotolerant fungus *Aspergillus variecolor* B-17	Weak cytotoxic and antioxidant activities	[147]
(−)-(*S*)-Variecolortide C (**313**)	Lichen-derived *Eurotium* sp. No. 17-11-8-1 from *Cladina grisea* collected in Changbaishan Mountain of China	-	[111]
(+)-(*R*)-Variecolortide C (**314**)	Lichen-derived *Eurotium* sp. No. 17-11-8-1 from *Cladina grisea* collected in Changbaishan Mountain of China	-	[111]
WIN 64745 (**315**)	*Aspergillus* sp. SC319	-	[148]
WIN 64821 (**316**)	*Aspergillus* sp. SC319	-	[148]

Note: IC_50_, median inhibitory concentration; MIC, minimum inhibitory concentration.

**Table 4:** Proline-Xaa cyclodipeptide analogs and their biological activities.

Name	Fungus and its Origin	Biological Activity	Ref.
Amoenamide A (**317**)	*Aspergillus amoenus* NRRL 35600	-	[153]
Cyclo(l-Pro–l-Ala) (**318**)	*Alternaria alternata*	-	[149]
	Phytopathogenic *Colletotrichum gloesporoides*	Inhibition of aflatoxin production in *Aspergillus flavus*	[150,151]
Cyclo(*trans*-4-hydroxy-l-Pro–l-Ala) (**319**)	Endophytic *Alternaria* *alternata* from grapevine	Antifungal activity on *Plasmopara viticola*	[154]
Cyclo(l-Pro–l-Gly) (**320**)	Phytopathogenic *Colletotrichum gloesporoides*	-	[150]
Cyclo(2-hydroxy-Pro–Gly) (**321**)	*Simplicillium* sp. YZ-11	-	[155]
Cyclo(l-Pro–l-Ile) (**322**)	Endophytic fungus *Alternaria* *tenuissima* from the bark of *Erythrophleum fordii*	-	[156]
	Endophytic *Aspergillus fumigatus*	-	[21]
	*Rhizoctonia solani*	-	[157]
Cyclo(l-Pro–d-Leu) (**323**)	Marine-derived *Chromocleista* sp. from a deep-water sediment sample collected in the Gulf of Mexico	-	[158]
Cyclo(l-Pro–l-Leu) (**324**)	Endophytic *Aspergillus fumigatus*	Weak inhibitory activity of β-glucuronidase release	[21]
	Phytopathogenic *Colletotrichum gloesporoides*	Phytotoxic, antitumoral and fungicide activity	[150]
	*Rhizoctonia solani*	-	[157]
	Endophytic *Alternaria* *tenuissima* from the bark of *Erythrophleum fordii*	-	[156]
Cyclo(Pro–Homoleucine) (**325**)	*Alternaria alternata*	-	[149]
Cyclo(4-hydroxy-*R*-Pro–*S*-Leu) = Cyclo(*cis*-4-hydroxy-d-Pro–l-Leu) (**326**)	Marine-sponge derived yeast *Aureobasidium pullulans* at Okinawa of Japan	-	[159]
	Mangrove-derived endophytic *Pestalotiopsis vaccinii* from a branch of *Kandelia candel*	-	[160]
Cyclo(*trans*-4-hydroxy-l-Pro–l-Leu) (**327**)	Endophytic *Alternaria* *alternata* from grapevine	Antifungal activity on *Plasmopara viticola*	[154]
	Endophytic *Alternaria* *tenuissima* from the bark of *Erythrophleum fordii*	-	[156]
Cyclo(d-Pro–d-Phe) (**328**)	Endophytic *Alternaria* *tenuissima* from the bark of *Erythrophleum fordii*	-	[156]
Cyclo(l-Pro–d-Phe) (**329**)	*Alternaria alternata*	-	[149]
	Marine-derived *Chromocleista* sp. from a deep-water sediment sample collected in the Gulf of Mexico	-	[158]
	Marine-derived *Penicillium bilaii*	-	[161]
Cyclo(6,7-en-Pro–l-Phe) (**330**)	Marine-derived *Chromocleista* sp. from a deep-water sediment sample collected in the Gulf of Mexico	-	[158]
Cyclo(4-Hydroxy-*R*-Pro–*S*-Phe) = Cyclo-(*cis*-4-Hydroxy-d-Pro–l-Phe) (**331**)	Marine-sponge derived yeast *Aureobasidium pullulans* at Okinawa of Japan	-	[159]
Cyclo(d-6-Hydroxy-Pro–l-Phe) = Cyclo(d-6-Hyp–l-Phe) (**332**)	Marine-derived *Chromocleista* sp. from a deep-water sediment sample collected in the Gulf of Mexico	-	[158]
	Marine-derived *Chromocleista* sp.	-	[162]
Cyclo(6-Hydroxy-l-Pro–l-Phe) = Cyclo(l-6-Hyp–l-Phe) (**333**)	Marine-derived *Chromocleista* sp. from a deep-water sediment sample collected in the Gulf of Mexico	-	[158]
	Marine-derived *Chromocleista* sp.	-	[162]
Cyclo(l-Pro–l-Phe) = Maculosin-2 (**334**)	*Alternaria* *alternata* from spotted knapweed (*Centaurea maculosa*)	Phytotoxic activity	[149,163]
Cyclo(*trans*-4-hydroxy-l-Pro–l-Phe) (**335**)	Endophytic *Alternaria* *alternata* from grapevine	Antifungal activity on *Plasmopara viticola*	[154]
Cyclo(l-Pro–l-Pro) (**336**)	Endophytic *Alternaria tenuissima* from the bark of *Erythrophleum fordii*	-	[156]
	Endophytic fungus *Stagonosporopsis oculihominis* from *Dendrobium huoshanense*	-	[164]
Cyclo(l-Pro–l-Tyr) = Maculosin-1 (**337**)	*Alternaria* *alternata* from spotted knapweed (*Centaurea maculosa*)	Phytotoxic activity	[149,163]
	Marine-derived *Chromocleista* sp. from a deep-water sediment sample collected in the Gulf of Mexico	-	[158]
	Marine-derived *Penicillium bilaii*	-	[161]
Cyclo(13,15-dichloro-l-Pro–l-Tyr) (**338**)	*Leptoxyphium* sp.	Inhibitory activity on CCL2-induced chemotaxis	[152]
Cyclo(l-Pro–d-Val) (**339**)	*Alternaria alternata*	-	[149]
	Endophytic *Alternaria* *tenuissima* from the bark of *Erythrophleum fordii*	-	[156]
Cyclo(d-Pro–l-Val) (**340**)	*Aspergillus* sp. F70609	Inhibitory activity on *β*-glucosidase	[165]
Cyclo(l-Pro–l-Val) (**341**)	Marine-derived *Chromocleista* sp. from a deep-water sediment sample collected in the Gulf of Mexico	-	[158]
	Phytopathogenic *Colletotrichum gloesporoides*	Phytotoxic and antibiotic activities	[150]
	Phytopathogenic *Fusarium oxysporum*	-	[166]
	Marine-derived *Penicillium bilaii*	-	[161]
	*Rhizoctonia solani*	-	[157]
(*R*)-2-(2-(Furan-2-yl)-oxoethyl- octahydropyrrolo[1,2-*a*] pyranine-1,4-dione (**342**)	Edible and medicinal *Armillaria mellea*	-	[167]
Macrophominol (**343**)	Phytopathogenic *Macrophomina phaseolina*	-	[168]
Taichunamide A (**344**)	*Aspergillus taichungensis* (IBT 19404)	-	[53]
Taichunamide B (**345**)	*Aspergillus taichungensis* (IBT 19404)	-	[53]

**Table 5:** Non-tryptophan–non-proline cyclodipeptide analogs and their biological activities.

Name	Fungus and its Origin	Biological Activity	Ref.
3-Acetamino-6-isobutyl-2,5-dioxopiperazine (**346**)	*Cordyceps sinensis*	Cytotoxic activity	[169]
Altenarizine A (**347**)	Endophytic *Alternaria alternata* from the root of *Ceratostigma griffithii*	-	[183]
Altenarizine B (**348**)	Endophytic *Alternaria alternata* from the root of *Ceratostigma griffithii*	-	[183]
Aurantiamine (**349**)	*Penicillium aurantiogriseum* var. a*urantiogriseum*	-	[184]
Azonazine (**350**)	*Aspergillus insulicola*	Anti-inflammatory activity	[170]
(3*S*)-6-Benzyl-3-isopropyl-1-methylpiperazine-2,5-dione (**351**)	Entomogenous *Paecilomyces tenuipes*	Moderate cytotoxicity against prostate cancer cells 22RV1 and DU-145	[185]
Cordycedipeptide A (**352**)	*Cordyceps sinensis*	Cytotoxic activity	[169]
Cyclo(Gly–Phe) (**353**)	Unidentified fungus from *Kandelia candel* leaf	-	[186]
Cyclo(Leu–Leu) (**354**)	Unidentified fungus from *Kandelia candel* leaf	-	[186]
Cyclo(Leu–Tyr) (**355**)	Unidentified fungus from *Kandelia candel* leaf	-	[186]
Cyclo(l-Leu–l-Val) (**356**)	Endophytic *Aspergillus fumigatus* from the stem of *Erythrophleum fordii*	-	[187]
Cyclo(l-Phe–l-Phe) (**357**)	*Penicillium nigricans*	Anthelmintic activity against *Hymenolepis nana* and *Schistosoma mansoni* in mice	[171,172]
	Endophytic *Epicoccum nigrum* from *Lysidice rhodostegia*	-	[188]
Cyclo(Phe–Ser) (**358**)	Endophytic *Alternaria* sp. FL25 from *Ficus carica*	Antiphytopathogenic fungal activity	[38]
	Insect pathogenic *Verticillium hemipterigenum*	Cytotoxic and antimicrobial activity	[173,174]
Cyclo(l-Phe-*N*-methyl-l-Tyr) (**359**)	*Geotrichum candidum*	Inhibitory activity against *Peronophythora litchii*	[189]
Cyclo(l-Tyr–l-Tyr) (**360**)	*Cordyceps sinensis*	-	[169]
Cyclopenin (**361**)	*Penicillium verrucosum* var. *cyclopium*	-	[124]
Cyclopenol (**362**)	*Penicillium verrucosum* var. c*yclopium*	-	[124]
	Mangrove endophytic *Penicillium sclerotiorum* from *Bruguiera gymnorrhiza*	-	[190]
Deoxymycelianamide (**363**)	Marine-derived *Gliocladium* sp.	Strong cytotoxic activity	[179]
Desferricoprogen (**364**)	Mud dauber wasp-derived *Talaromyces* sp. CMB-W045	-	[191]
Diatretol (**365**)	*Clitocybe diatreta*	Weak antibacterial activity	[175]
(6*S*)-3-(1,3-Dihydroxypropyl)-6-(2-methylpropyl)piperzaine-2,5-dione (**366**)	Plant endophytic *Trichosporum* sp. from the seeds of *Trigonella foenum*-*graecum*	Antileishmanial activity against *Leishmania donovani* with IC_50_ value of 96.3 μg/mL	[192]
(6*R*)-3-(1,3-Dihydroxypropyl)-6-(2-methylpropyl)piperzaine-2,5-dione (**367**)	Plant endophytic *Trichosporum* sp. from the seeds of T*rigonella foenum*-*graecum*	Antileishmanial activity against *Leishmania donovani* with IC_50_ value of 82.5 μg/mL	[192]
Dimerumic acid (**368**)	*Monascus anka*	Antioxidant activity	[176,177]
	Mud dauber wasp-derived *Talaromyces* sp. CMB-W045	Demonstratinghigh affinity for Fe(III)	[191]
	*Monascus anka*	Antioxidant activity by inhibition on lipid peroxidation and hemeprotein-mediated oxidation	[193]
Diphenylalazine A (**369**)	*Epicoccum nigrum* colonizing on *Cordyceps sinensis*	Inhibitory effects on HIV-1 replication in C8166 cells	[194]
Diphenylalazine B (**370**)	*Epicoccum nigrum* colonizing on *Cordyceps sinensis*	-	[194]
Diphenylalazine C (**371**)	Tin mine tailings-derived *Schizophyllum commune*	Weak antibacterial and cytotoxic activities	[195]
Eleutherazine B (**372**)	Mud dauber wasp-derived *Talaromyces* sp. CMB-W045	-	[191]
Fusaperazine C (**373**)	Endophytic *Fusarium* sp. from *Viguiera arenaria*	-	[196]
Gliocladride (**374**)	Marine-derived *Gliocladium* sp.	Cytotoxic activity	[178]
Gliocladride A (**375**)	Marine-derived *Gliocladium* sp.	Moderate cytotoxic activity	[179]
Gliocladride B (**376**)	Marine-derived *Gliocladium* sp.	Moderate cytotoxic activity	[179]
Golmaenone (**377**)	Marine-derived *Aspergillus* sp.	Radical scavenging activity against DPPH, UV-A protecting activity	[103]
	Marine mudflat sediment derived *Chaetomium cristatum* collected at Suncheon Bay of Korea	Radical-scavenging activity against DPPH with IC_50_ value of 20 μM	[134]
Gunnilactam A (**378**)	Entomogenous *Paecilomyces gunnii*	Cytotoxic activity against human prostate cancer C42B cells	[197]
Gunnilactam B (**379**)	Entomogenous *Paecilomyces gunnii*	-	[197]
Gunnilactam C (**380**)	Entomogenous *Paecilomyces gunnii*	-	[197]
14-Hydroxy-cyclopeptine (**381**)	*Aspergillus* sp. SCSIOW2	Inhibition of nitric oxide production with IC_50_ value of 40.3 μg/mL in a lipopolysaccharide and recombinant mouse interferon-γ-activated macrophage-like cell line	[198]
Hypocreasin (**382**)	*Hypocrea* spp.	-	[199]
3-Isopropyl-6-isobutyl-2,5-dioxopiperazine (**383**)	*Cordyceps sinensis*	-	[169]
JBIR-74 (**384**)	Marine-derived *Aspergillus* sp. fS14 from the unidentified marine sponge	-	[200]
JBIR-75 (**385**)	Marine-derived *Aspergillus* sp. fS14 from the unidentified marine sponge	-	[200]
Mactanamide (**386**)	Marine-derived *Aspergillus* sp.	Fungistatic activity to *Candida albicans*	[180]
MPC1001H (**387**)	*Podospora australis*	-	[201]
NBRI16716A (**388**)	*Perisporiopsis melioloides* Mer-f16716	Cytotoxic activity	[181]
NBRI16716B (**389**)	*Perisporiopsis melioloides* Mer-f16716	Cytotoxic activity	[181]
NBRI16716C (**390**)	*Perisporiopsis melioloides* Mer-f16716	-	[181]
Penicillivinacine (**391**)	Marine-derived *Penicillium vinaceum*	Antimigratory activity	[22]
Phenylahistin (**392**)	*Aspergillus ustus* NSC-F038	Growth inhibition of various tumor cell lines	[182]
PJ147 (**393**)	Marine-derived *Gliocladium* sp. YUP08 from soil	Cytotoxic activity on A375-S2, Hela, P388, A-549, HL-60, and BEL-7420 cell lines	[202,203]
PJ157 (**394**)	Marine-derived *Gliocladium* sp. YUP08 from soil	-	[202]
Pre-aurantiamine (**395**)	Marine-derived *Aspergillus aculeatus* CRI322-03 from the sponge *Stylissa flabeliformis*	-	[204]
Spirobrocazine C (**396**)	Mangrove-derived *Penicillium brocae* MA-231 from *Avicennia marina*	Moderate cytotoxic and antibacterial activities	[205]
Talarazine A (**397**)	Mud dauber wasp-derived *Talaromyces* sp. CMB-W045	-	[191]
Talarazine B (**398**)	Mud dauber wasp-derived *Talaromyces* sp. CMB-W045	-	[191]
Talarazine C (**399**)	Mud dauber wasp-derived *Talaromyces* sp. CMB-W045	-	[191]
Talarazine D (**400**)	Mud dauber wasp-derived *Talaromyces* sp. CMB-W045	-	[191]
Talarazine E (**401**)	Mud dauber wasp-derived *Talaromyces* sp. CMB-W045	-	[191]
Terretrione A (**402**)	Marine-derived *Penicillium vinaceum*	Antimigratory activity	[22]
Waspergillamide A (**403**)	*Aspergillus* sp. CMB-W031	-	[206]

Note: IC_50_, median inhibitory concentration.

**Table 6:** Fungal 1,4-bridged epiplythiodioxopiperazine analogs and their biological activities.

Name	Fungus and its Origin	Biological Activity	Ref.
3822-A (**404**)	*Stereum hirsutum* HKI 0195	-	[219]
A26771A (**405**)	*Penicillium turbatum*	Antiviral and antibacterial activity	[226]
A26771 C (**406**)	*Penicillium turbatum*	Antiviral and antibacterial activity	[226]
Acetylapoaranotin (**407**)	Marine-derived *Aspergillus* sp. KMD 901	Directly cytotoxic and apoptosis inducing effects on HCT116 colon cancer cell lines	[212]
Acetylaranotin (**408**)	Marine-derived *Aspergillus* sp. KMD 901	Directly cytotoxic and apoptosis inducing effects on HCT116 colon cancer cell lines	[212]
Apoaranotin (**409**)	*Arachniotus aureus*	-	[227]
Aranotin (**410**)	*Arachniotus aureus*	-	[227]
Bionectin A (**411**)	*Bionectra byssicola* F120	Anti-MRSA activity	[228]
Bionectin B (**412**)	*Bionectra byssicola* F120	Anti-MRSA activity	[228]
Brocazine A (**413**)	Endophytic *Penicillium brocae* MA-231 from mangrove *Avicennia marina*	Cytotoxic activity	[229]
Brocazine B (**414**)	Endophytic *Penicillium brocae* MA-231 from mangrove *Avicennia marina*	Cytotoxic activity	[229]
Brocazine C (**415**)	Endophytic *Penicillium brocae* MA-231 from mangrove *Avicennia marina*	-	[229]
Brocazine D (**416**)	Endophytic *Penicillium brocae* MA-231 from mangrove *Avicennia marina*	-	[229]
Brocazine E (**417**)	Endophytic *Penicillium brocae* MA-231 from mangrove *Avicennia marina*	Cytotoxic activity	[229]
Brocazine F (**418**)	Endophytic *Penicillium brocae* MA-231 from mangrove *Avicennia marina*	Cytotoxic activity	[229]
Brocazine G (**419**)	Marine-derived *Penicillium brocae* MA-231 from the mangrove *Avicennia marina*	Cytotoxicity against both sensitive and cisplatin-resistant human ovrian cancer cells and strong antimicrobial activity on pathogenic *Staphylococcus aureus*	[205]
Chaetocin = Chaetocin A (**420**)	*Chaetomium minutum*	Antibacterial, cytostatic, inhibitory activity on lysine-specific histone methyltransferases	[209]
Chaetocin B (**421**)	*Chaetomium* sp.	Cytotoxic activity	[210]
Chaetocin C (**422**)	*Chaetomium* sp.	Cytotoxic activity	[210]
Chaetocochin B (**423**)	*Chaetomium cochliodes*	Cytotoxic activity	[211]
Chaetocochin C (**424**)	*Chaetomium cochliodes*	Cytotoxic activity	[211]
Chetomin (**425**)	Marine mudflat sediment derived *Chaetomium cristatum* collected at Suncheon Bay of Korea	Radical-scavenging activity against DPPH with an IC_50_ value of 15 μM	[134]
Chetoseminudin A (**426**)	*Chaetomium globosum*	Antibacterial activity	[230]
	*Chaetomium seminudum*	Immunomodulatory activity	[231]
Chetracin A (**427**)	*Chaetomium* spp.	Cytotoxic activity	[210]
Chetracin B (**428**)	Antarctic psychrophilic fungus *Oidiodendron truncatum*	Cytotoxic activity	[137]
Chetracin C (**429**)	Antarctic psychrophilic fungus *Oidiodendron truncatum*	Cytotoxic activity	[137]
Cristazine (**430**)	Mudflat-sediment-derived *Chaetomium cristatum*	Radical-scavenging activity, cytotoxic activity against human cervical carcinoma (HeLa) cells	[134]
Dehydrogliotoxin (**431**)	*Gliocladium flavofuscum*	Antituberculosis activity	[232]
	*Gliocladium virens*	Antituberculosis activity	[233]
Deoxyapoaranotin (**432**)	Marine-derived *Aspergillus* sp. KMD 901	Directly cytotoxic and apoptosis inducing effects on HCT116 colon cancer cell lines	[212]
11‘-Dexoyverticillin A (**433**)	*Gliocladium roseum* from submerged wood	Antinematodal activity	[222]
11,11‘-Dihydroxychaetocin (**434**)	*Verticillium tenerum*	Antibacterial and antimitotic activities	[234]
Dithiosilvatin (**435**)	*Aspergillus silvaticus*	-	[235]
Emestrin (**436**)	*Cladorrhinum* sp. KY4922	Antiproliferative activity	[236]
Emestrin B (**437**)	*Emericella striata*	Antifungal activity	[237]
Emestrin C = MPC1001 (**438**)	*Podospora australis*	Antifungal activity	[201]
	*Cladorrhinum* sp. KY4922	Antiproliferative activity	[236]
	*Cladorrhinum* sp. KY4922	Antitumor and antibacterial activity	[220]
Emestrin D = MPC1001D (**439**)	*Podospora australis*	Antifungal activity	[201]
	*Cladorrhinum* sp. KY4922	Antiproliferative activity	[236]
Emestrin E (**440**)	*Podospora australis*	Antifungal activity	[201]
Emestrin F (**441**)	*Armillaria tabescens*	Antifungal activity	[238]
Emestrin G (**442**)	*Armillaria tabescens*	-	[238]
Emestrin J (**443**)	*Podospora australis*	-	[201]
Emethallicin A (**444**)	*Emericella heterothallica*	Inhibitory activity on histamine release from mast cells	[213]
Emethallicin B (**445**)	*Emericella heterothallica*	Inhibitory activity on histamine release from mast cells	[214]
Emethallicin C (**446**)	*Emericella heterothallica*	Inhibitory activity on histamine release from mast cells	[214]
Emethallicin D (**447**)	*Emericella heterothallica*	Inhibitory activity on histamine release from mast cells	[214]
Emethallicin E (**448**)	*Emericella heterothallica*	Inhibitory activity on histamine release from mast cells	[215]
Emethallicin F (**449**)	*Emericella heterothallica*	Inhibitory activity on histamine release mast cells	[215]
Epicoccin T (**450**)	Endophytic fungus *Epicoccum nigrum* from the leaves *Lysidice rhodostegia*	-	[188]
Epicoccin U (**451**)	Tin mie tailings-derived *Schizophyllum commune*	Weak antibacterial and cytotoxic activities	[195]
Epicorazine A (**452**)	*Capnodium* sp. from palm leaf litter collected in Quetzalito, Guatemala	-	[239]
	*Epicoccum purpurascens*	-	[240]
	Marine-derived *Penicillium brocae* MA-231 from the mangrove plant *Avicennia marina*	-	[229]
	Marine-derived *Phoma* sp. OUCMDZ-1847 from the mangrove plant *Kandelia candel*	Cytotoxic activity	[241]
	*Podaxis pistillaris*	Antibacterial and cytotoxic activities	[242]
	Basidiomycete *Stereum hirsutum* HKI 0195	Cytotoxic activity against HeLa cells and antiproliferative effects against several mouse fibrobalst and cancer cell lines	[219]
Epicorazine B (**453**)	*Epicoccum purpurascens*	-	[240]
	Marine-derived *Phoma* sp. OUCMDZ-1847 from the mangrove *Kandelia candel*	Cytotoxic activity	[241]
	*Podaxis pistillaris*	Antibacterial and cytotoxic activities	[242]
	Basidiomycete *Stereum hirsutum* HKI 0195	Cytotoxic activity against HeLa cells and antiproliferative effects against several mouse fibroblast and cancer cell lines	[219]
Epicorazine C (**454**)	Marine-derived *Phoma* sp. OUCMDZ-1847 from the mangrove plant *Kandelia candel*	Cytotoxic activity	[241]
	*Podaxis pistillaris*	Antibacterial activity	[242]
	*Stereum hirsutum* HKI 0195	Antibacterial, antifungal, antiproliferative and cytotoxic activities	[219]
Gliocladine A (**455**)	*Gliocladium roseum* from submerged wood	Atinematodal activity	[222]
Gliocladine B (**456**)	*Gliocladium roseum* from submerged wood	Atinematodal activity	[222]
Gliocladine C (**457**)	*Gliocladium roseum* from submerged wood	Atinematodal activity	[222]
Gliocladine D (**458**)	*Gliocladium roseum* from submerged wood	Atinematodal activity	[222]
Gliocladine E (**459**)	*Gliocladium roseum* from submerged wood	Atinematodal activity	[222]
Glionitrin A (**460**)	Coculture of the fungus *Aspergillus fumigatus* KMC-901 and the bacterium *Sphingomonas* sp. KMK-001	Antibacerial and cytotoxic activities	[243]
Gliotoxin (**461**)	*Aspergillus fumigatus*	Cytotoxic activity	[217]
	*Gliocladium flavofuscum*	-	[232]
	Deep-sea derived *Aspergillus* sp. SCSIO Ind09F01	Anti-tuberculosis and cytotoxic activities	[35]
Hyalodendrin (**462**)	Marine-derived *Asteromyces cruciatusis* 763 from an unidentified decaying green alga	-	[244]
Hyalodendrin-S_3_ (**463**)	Unidentified fungus NRRL 3888	-	[245]
Hyalodendrin-S_4_ (**464**)	*Hyalodendron* sp.	-	[246]
Leptosin A (**465**)	*Leptosphaeria* sp. from a marine alga	Cytotoxicity	[247]
Leptosin B (**466**)	*Leptosphaeria* sp. from a marine alga	-	[247]
Leptosin C (**467**)	*Leptosphaeria* sp. from a marine alga	Cytotoxicity	[247]
Leptosin D (**468**)	*Leptosphaeria* sp. from a marine alga	-	[247]
Leptosin E (**469**)	*Leptosphaeria* sp. from a marine alga	-	[247]
Leptosin F (**470**)	*Leptosphaeria* sp. from a marine alga	-	[247]
Leptosin G (**471**)	*Leptosphaeria* sp. from a marine alga	Cytotoxicity	[248]
Leptosin G1 (**472**)	*Leptosphaeria* sp. from a marine alga	Cytotoxicity	[248]
Leptosin G2 (**473**)	*Leptosphaeria* sp. from a marine alga	Cytotoxicity	[248]
Leptosin H (**474**)	*Leptosphaeria* sp. from a marine alga	Cytotoxicity	[248]
Leptosin I (**475**)	*Leptosphaeria* sp. from a marine alga	Cytotoxicity	[249]
Leptosin J (**476**)	*Leptosphaeria* sp. from a marine alga	Cytotoxicity	[249]
Leptosin K (**477**)	*Leptosphaeria* sp. from a marine alga	Cytotoxicity on P388 cells	[250]
Leptosin K_1_ (**478**)	*Leptosphaeria* sp. from a marine alga	Cytotoxicity on P388 cells	[250]
Leptosin K_2_ (**479**)	*Leptosphaeria* sp. from a marine alga	Cytotoxicity on P388 cells	[250]
Leptosin M (**480**)	*Leptosphaeria* sp. from a marine alga	Cytotoxicity on P388 cells; Inhibition on two protein kinases, PTK and CaMKIII, and human topoisomerase II	[251]
Leptosin M_1_ (**481**)	*Leptosphaeria* sp. from a marine alga	Cytotoxicity on P388 cells	[251]
Leptosin N (**482**)	*Leptosphaeria* sp. from a marine alga	Cytotoxicity on P388 cells	[251]
Leptosin N_1_ (**483**)	*Leptosphaeria* sp. from a marine alga	Cytotoxicity on P388 cells	[251]
Melinacidin II (**484**)	*Acrostalagmus cinnabarinus* var. *melinacidinus*	Antibacterial activity	[252]
Melinacidin III (**485**)	*Acrostalagmus cinnabarinus* var. *melinacidinus*	Antibacterial activity	[252]
Melinacidin IV (**486**)	*Acrostalagmus cinnabarinus* var. *melinacidinus*	Antibacterial activity	[252]
	Antarctic psychrophilic fungus *Oidiodendron truncatum*	Cytotoxic activity	[137]
MPC1001B (**487**)	*Cladorrhinum* sp. KY4922	Antiproliferative activity	[236]
MPC1001C (**488**)	*Cladorrhinum* sp. KY4922	Antiproliferative activity	[236]
	*Podospora australis*	Antifungal activity	[201]
MPC1001E (**489**)	*Cladorrhinum* sp. KY4922	Antiproliferative activity	[236]
Phomalirazine (**490)**	*Leptosphaeria maculans*	Phytotoxic activity	[253]
Phomazine C (**491**)	Marine-derived *Phoma* sp. OUCMDZ-1847 from the mangrove plant *Kandelia candel*	-	[241]
Plectosphaeroic acid C (**492**)	Marine-derived *Plectosphaerella cucumerina*	Inhibtion of indoleamine 2,3-dioxygenase	[254]
Rostratin A (**493**)	Endophytic *Epicoccum nigrum* from the leaves *Lysidice rhodostegia*	-	[188]
	*Exserohilum rostratum*	Moderate cytotoxicity	[218]
Rostratin B (**494**)	*Exserohilum rostratum*	Moderate cytotoxicity	[218]
Rostratin C (**495**)	*Exserohilum rostratum*	Moderate cytotoxicity	[218]
Rostratin D (**496**)	*Exserohilum rostratum*	Moderate cytotoxicity	[218]
Sch52900 (4**97**)	*Gliocladium roseum* from submerged wood	Antinematodal activity	[222]
Sch52901 (**498**)	*Gliocladium roseum* from submerged wood	Antinematodal activity	[222]
Secoemestrin C (**499**)	*Emericella foveolata*	-	[255]
Secoemestrin D (**500**)	*Podospora australis*	-	[201]
	Endophytic fungus *Emericella* sp. AST0036 from healthy leaf tissue of *Astragalus lentiginosus*	Cytotoxic activity	[221]
Sirodesmin A (**501**)	*Sirodesmium diversum*	Antiviral activity	[256]
Sirodesmin B (**502**)	*Leptosphaeria maculans*	Phytotoxic activity	[257]
	*Sirodesmium diversum*	Antiviral activity	[256]
Sirodesmin C (**503**)	*Leptosphaeria maculans*	Phytotoxic activity	[257]
	*Sirodesmium diversum*	Antiviral activity	[256]
Sirodesmin G = Sirodesmin PL (**504**)	*Leptosphaeria maculans*	Phytotoxic activity	[257,258]
	*Sirodesmium diversum*	Antiviral activity	[256]
Sirodesmin H (**505**)	*Leptosphaeria maculans*	Phytotoxic activity	[257]
Sporidesmin A = Sporidesmin (**506**)	*Pithomyces chartarum*	Immunoregulatory activity	[259,260]
	*Delitschia corticola*	Antibacterial and antifungal activities	[261]
Sporidesmin B (**507**)	*Pithomyces chartarum*	-	[259]
Sporidesmin C (**508**)	*Pithomyces chartarum*	-	[262]
Sporidesmin E (**509**)	*Penicillium terlikowskii*	-	[263]
Sporidesmin G (**510**)	*Pithomyces chartarum*	Antiproliferative, cytotoxic, immunomodulatory, antiviral, antibacterial, antifungal activities	[264]
Sporidesmin H (**511**)	*Pithomyces chartarum*	Antiproliferative, cytotoxic, immunomodulatory, antiviral, antibacterial, antifungal activities	[265]
Sporidesmin J (**512**)	*Pithomyces chartarum*	Antiproliferative, cytotoxic, immunomodulatory, antiviral, antibacterial, antifungal activities	[265]
T988 A (**513**)	*Tilachidium* sp.	Cytotoxic activity	[266]
	Antarctic psychrophilic fungus *Oidiodendron truncatum*	Cytotoxic activity	[211]
T988 C (**514**)	*Tilachidium* sp.	Cytotoxic activity	[266]
	Antarctic psychrophilic fungus *Oidiodendron truncatum*	Cytotoxic activity	[211]
Verticillin A (**515**)	*Gliocladium roseum* from submerged wood	Antinematodal and cytotoxic activities	[222]
	*Verticillium* sp.	-	[223]
Verticillin B (**516**)	*Verticillium* sp.	-	[223]
Verticillin C (**517**)	*Verticillium* sp.	-	[223]
Verticillin D (**518**)	*Bionectria byssicola*	Antibacterial activity	[225]
	*Gliocladium catenulatum*	Antibacterial activity	[224]
Verticillin E (**519**)	*Gliocladium catenulatum*	Antibacterial activity	[224]
Verticillin F (**520**)	*Gliocladium catenulatum*	Antibacterial activity	[224]
Verticillin G (**521**)	*Bionectra byssicola*	Antibacterial activity	[225]

Note: IC_50_, median inhibitory concentration.

**Table 7:** Fungal analogs with sulfur-bridge outside 2,5-DKP ring and their biological activities.

Name	Fungus and Its Origin	Biological Activity	Ref.
Adametizine A = *N*-methyl pretrichodermamide B (**522**)	Marine sponge-derived *Penicillium adametzioides* AS-53	Lethal activity against brine shrimp and antibacterial activity	[269]
Adametizine B = Pretrichodermamide C (**523**)	Marine sponge-derived *Penicillium adametzioides* AS-53	-	[269]
Aspirochlorine (**524**)	*Aspergillus flavus*	Antifungal activity on azole-resitant *Candida albicans*	[107]
Chlorotrithiobrevamide (**525**)	*Trichoderma* cf*. brevicompactum*	Cytotoxic effects against Jurkat cells with IC_50_ values of 16 µM	[270]
DC1149B (**526**)	*Trichoderma* cf. *brevicompactum*	Cytotoxic effect against Jurkat cells with IC_50_ values of 5.1 µM	[270,271]
DC1149R(**527**)	*Trichoderma* cf. *brevicompactum*	-	[271]
Epicoccin A (**528**)	*Cordyceps*-colonizing *Epicoccum nigrum*	Moderate antimicrobial activity	[268]
	Endophytic *Epicoccum nigrum* from *Lysidice rhodostegia*	-	[188]
Epicoccin B (**529**)	*Cordyceps*-colonizing *Epicoccum nigrum*	-	[268]
	Endophytic *Epicoccum nigrum* from *Lysidice rhodostegia*	-	[188]
Epicoccin C (**530**)	*Cordyceps*-colonizing *Epicoccum nigrum*	-	[268]
	Endophytic *Epicoccum nigrum* from *Lysidice rhodostegia*	-	[188]
Epicoccin D (**531**)	*Cordyceps*-colonizing *Epicoccum nigrum*	-	[268]
Epicoccin E (**532**)	*Cordyceps*-colonizing *Epicoccum nigrum*	-	[194]
	Endophytic *Epicoccum nigrum* from *Lysidice rhodostegia*	-	[188]
Epicoccin F (**533**)	*Cordyceps*-colonizing *Epicoccum nigrum*	-	[194]
Epicoccin I (**534**)	Endophytic *Epicoccum nigrum* from *Lysidice rhodostegia*	-	[188]
Epicoccin M (**535**)	Endophytic *Epicoccum nigrum* from *Lysidice rhodostegia*	-	[188]
Epicoccin N (**536**)	Endophytic *Epicoccum nigrum* from *Lysidice rhodostegia*	-	[188]
Epicoccin O (**537**)	Endophytic *Epicoccum nigrum* from *Lysidice rhodostegia*	-	[188]
Epicoccin P (**538**)	Endophytic *Epicoccum nigrum* from *Lysidice rhodostegia*	-	[188]
Epicoccin Q (**539**)	Endophytic *Epicoccum nigrum* from the leaves of *Lysidice rhodostegia*	-	[188]
Epicoccin R (**540**)	Endophytic *Epicoccum nigrum* from *Lysidice rhodostegia*	-	[188]
Epicoccin S (**541**)	Endophytic *Epicoccum nigrum* from *Lysidice rhodostegia*	-	[188]
Gliovirin (**542**)	*Trichoderma* cf. *brevicompactum*	-	[270,271]
Iododithiobrevamide (**543**)	*Trichoderma* cf. *brevicompactum*	-	[271]
Outovrin A (**544**)	Endophytic *Penicillium raciborskii* from *Rhododendron tomentosum*	-	[272]
Outovirin B (**545**)	Endophytivc *Penicillium raciborskii* from *Rhododendron tomentosum*	-	[272]
Outovirin C (**546**)	Endophytic *Penicillium raciborskii* from *Rhododendron tomentosum*	Antifungal activity	[272]
Penicisulfuranol A (**547**)	Mangrove-derived *Penicillium janthinellum* HDN13-309 from the roots of *Sonneratia caseolaris*	Cytotoxic activity on the cell lines HeLa and HL-60	[273]
Penicisulfuranol B (**548**)	Mangrove-derived *Penicillium janthinellum* HDN13-309 from the roots of *Sonneratia caseolaris*	Cytotoxic activity on the cell lines HeLa and HL-60	[273]
Penicisulfuranol C (**549**)	Mangrove-derived *Penicillium janthinellum* HDN13-309 from the roots of *Sonneratia caseolaris*	Cytotoxic activity on the cell lines HeLa and HL-60	[273]
Pretrichodermamide A (**550**)	*Trichoderma* cf. *brevicompactum*	-	[270,271]
	*Trichoderma* sp. BCC 5926	Activity against *Mycobacterium tuberculosis* H37Ra with an MIC value of 12.5 µg/mL	[274]
Tetrathioaspirochlorine (**551**)	*Aspergillus flavus*	Antifungal activity on azole-resitant *Candida albicans*	[107]
Trithioaspirochlorine (**552**)	*Aspergillus flavus*	-	[107]
Vertihemiptellide A (**553**)	Insect pathogenic *Verticillium hemipterigenum* BBC 1449	Inhibitory activity against *Mycobacterium tuberculosis* H_37_Ra, moderate cytotoxic activity	[174]
Vertihemiptellide B (**554**)	Insect pathogenic *Verticillium hemipterigenum* BBC 1449	Inhibitory activity against *Mycobacterium tuberculosis* H_37_Ra, moderate cytotoxic activity	[174]

Note: IC_50_, median inhibitory concentration; MIC, minimum inhibitory concentration.

**Table 8:** Nonbridged methylthio-containing cyclodipeptide analogs from fungi and their biological activities.

Name	Fungus and its Origin	Biological Activity	Ref.
Alternarosin A (**555**)	Marine-derived *Alternaria raphani*	Weak antimicrobial activity	[276]
Asteroxepin (**556**)	*Aspergillus terreus*	-	[284]
(*Z*)-6-Benzylidene-3-hydroxymethyl-1,4-dimethyl-3-methylsulfanylpiperazine-2,5-dione (**557**)	Marine-derived unidentified strain CRIF2 of the order Pleosporales	Weak cytotoxic activity	[285]
Bilain A (**558**)	Marine-derived *Penicillium bilaii*	-	[161]
Bilain B (**559**)	Marine-derived *Penicillium bilaii*	-	[161]
Bilain C (**560**)	Marine-derived *Penicillium bilaii*	-	[161]
Bionectin C (**561**)	*Bionectra byssicola* F120	Anti-MRSA activity	[228]
Bisdethiobis(methylsulfanyl)acetylapoaranotin (**562**)	*Aspergillus terreus* BCC 4651	-	[286]
Bisdethiobis(methylsulfanyl)acetylaranotin = Bisdethiodi(methylthio)-acetylaranotin (**563**)	*Aspergillus terreus* BCC 4651	-	[286]
Bisdethiobis(methylsulfanyl)apoaranotin = Bisdethiodi(methylthio)-acetylapoaranotin (**564**)	*Aspergillus terreus* BCC 4651	Weak antimycobacterial activity	[286]
Bisdethiobis(methylsulfanyl)aranotin (**565**)	*Aspergillus terreus* BCC 4651	-	[286]
Bisdethiobis(methylthio) gliotoxin (**566**)	Marine-derived *Aspergillus fumigatus*	-	[278]
	*Aspergillus fumigatus* from saltwater	Moderate trypanocidal activity	[287]
	Endophytic *Colletotrichum gloeosporioides* from *Viguiera robusta*	Specific inhibitor of the platelet activating factor and antibacterial activity	[196]
	Marine-derived fungus *Pseudallescheria* sp.	Antibacterial activity	[280]
Bisdethiodi(methylthio)-1-demethylhyalodendrin (**567**)	Insect pathogenic *Verticillium hemipterigenum* BCC 1449	Inhibitory activity against *Mycobacterium tuberculosis* H37Ra; moderate cytotoxic activity	[174]
(3*R*,6*R*)-Bisdethiodi(methylthio)-hyalodendrin (**568**)	Marine-derived unidentified strain CRIF2 of the order Pleosporales	Weak cytotoxic activity	[285]
	*Cordyceps*-colonizing fungus *Isaria farinosa*	-	[288]
*cis*-Bis(methylthio)silvatin = *cis*-Bisdethiodi(methylthio) silvatin (**569**)	*Fusarium chlamydosporum* from the marine alga *Carpopeltis affinis*	Weak cytotoxic activity against P388 lymphocytic leukemia cells	[277]
	Marine-derived *Penicillium bilaii*	Weak cytotoxicity against NS-1 cells	[161]
	Plant endophytic *Penicillium* sp.	Antibacterial activity against *Staphylococcus aureus* with an MIC value of 43.4 μg/mL	[289]
Bis-*N*-norgliovictin (**570**)	*Gliocladium virens*	-	[233]
	Marine-derived *Aspergillus fumigatus*	-	[278]
	Marine-derived fungus *Neosartorya pseudofischeri*	-	[136]
	Marine-derived *Dichotomomyces* sp. L-8	-	[113]
	-	Inhibitory activity on LPS-induced inflammation in macrophages	[279]
Chaetocochin A (**571**)	*Chaetomium cochliodes*	Cytotoxic activity	[211]
Chetoseminudin B (**572**)	*Nectria inventa*	Trypanocidal activity in the whole cell assay of *Trypanosoma brucei*	[287]
Chetoseminudin C (**573**)	Antarctic psychrophilic fungus *Oidiodendron truncatum*	-	[137]
	*Chaetomium seminudum*	-	[231]
Chetoseminudin E (**574**)	Endophytic fungus *Chaetomium* sp. 88194	-	[290]
Chetracin D (**575**)	Antarctic psychrophilic fungus *Oidiodendron truncatum*	Cytotoxic activity	[137]
Colletopiperazine (**576**)	Endophytic *Colletotrichum gloeosporioides* from *Viguiera robusta*	-	[196]
Dehydroxybisdethiobis(methylthio) gliotoxin (**577**)	Marine-derived *Pseudallescheria* sp.	Antibacterial activity	[280]
Dethio-tetra(methylthio)chetomin (**578**)	*Chaetomium cochliodes*	Cytotoxic activity	[211]
Dichotocejpin A (**579**)	*Dichotomomyces cejpii* FS110	Inhibitory activity against α-glucosidase	[112]
Didehydrobisdethiobis (methylthio) gliotoxins (**580**)	Marine-derived *Aspergillus sydowi* from a driftwood sample	-	[10]
Epicoccin G (**581**)	*Cordyceps*-colonizing *Epicoccum nigrum*	Inhibitory effect on HIV-1 replication in C8166 cells	[194]
ent-Epicoccin G (**582**)	Endophytic *Epicoccum nigrum* from *Lysidice rhodostegia*	Inhibitory activity against the release of β-glucuronidase in rat polymorphonuclear leukocytes induced by platelet-activating factor	[188]
Epicoccin H (**583**)	*Cordyceps*-colonizing *Epicoccum nigrum*	Inhibitory effect on HIV-1 replication in C8166 cells	[194]
Epicoccin J (**584**)	Endophytic *Epicoccum nigrum* from *Lysidice rhodostegia*	-	[188]
Epicoccin K (**585**)	Endophytic *Epicoccum nigrum* from *Lysidice rhodostegia*	-	[188]
Epicoccin L (**586**)	Endophytic *Epicoccum nigrum* from *Lysidice rhodostegia*	-	[188]
Emestrin H (**587**)	*Podospora australis*	-	[201]
Emestrin I (**588**)	*Podospora australis*	-	[201]
Emestrin K (**589**)	*Podospora australis*	-	[201]
FR106969 (**590**)	*Penicillium citrinum*	Inhibitory activity against PAF-induced rabbit platelet aggregation	[281]
Fusaperazine A (**591**)	*Fusarium chlamydosporum* OUPS-N124 from the marine alga *Carpopeltis affinis*	Weak cytotoxic activity	[277]
Fusaperazine B (**592**)	*Fusarium chlamydosporum* OUPS-N124 from the marine alga *Carpopeltis affinis*	-	[277]
Fusaperazine E (**593**)	Endophytic *Penicillium crustosum* from *Viguiera robusta*	-	[196]
Gliocladin A (**594**)	*Gliocladium roseum* PS-N132	Cytotoxicity against murine P388 lymphocytic leukemia cells	[125]
Gliocladin B (**595**)	*Gliocladium roseum* PS-N132	Cytotoxicity against murine P388 lymphocytic leukemia cells	[125]
Glionitrin B (**596**)	Coculture of the fungus *Aspergillus fumigatus* KMC-901 and the bacterium *Sphingomonas* sp. KMK-001	Suppression of DU145 cell invasion	[291]
Glioperazine (**597**)	*Gliocladium roseum* PS-N132	Cytotoxic activity	[125]
	*Bionectra byssicola* F120	-	[126]
Glioperazine B (**598**)	*Bionectra byssicola* F120	Weak antibacterial activity	[126]
Gliovictin = (−)-Gliovictin = A26771E (**599**)	Marine-derived *Asteromyces cruciatusis* 763 from an unidentified decaying green alga	-	[244]
	Marine-derived *Asteromyces cruciatus*	-	[292]
Haematocin (**600**)	Phytopathogenic *Nectria haematococca*	Antifungal activity by inhibiting spore-germination and germ-tube elongation	[282]
3-[(4-Hydroxyphenyl)-methyl]-1,4-dimethyl-3,6-bis(methylthio)-2,5-piperazinedione (**601**)	*Fusarium chlamydosporum* OUPS-N124 from the marine alga *Carpopeltis affinis*	-	[277]
Leptosin O (**602**)	*Leptosphaeria* sp. from a marine alga	Cytotoxicity on P388 cells	[131]
Leptosin P (**603**)	*Leptosphaeria* sp. from a marine alga	Cytotoxicity on P388 cells	[131]
Leptosin Q (**604**)	*Leptosphaeria* sp. from a marine alga	Cytotoxicity on P388 cells	[131]
Leptosin R (**605**)	*Leptosphaeria* sp. from a marine alga	Cytotoxicity on P388 cells	[131]
MPC1001F (**606**)	*Cladorrhinum* sp. KY4922	Antifungal activity	[236]
	*Podospora australis*	-	[201]
Mycoediketopiperazine (**607**)	*Papularia* sp.	Cytotoxicity on KB cells	[283]
^1^*N*-Norgliovictin (**608**)	Marine-derived *Asteromyces cruciatusis* 763 from an unidentified decaying green alga	-	[244]
Oidioperazine A (**609**)	Antarctic psychrophilic fungus *Oidiodendron truncatum*	-	[137]
Penicibrocazine A (**610**)	Marine-derived *Penicillium brocae* MA-231 from the mangrove plant *Avicennia marina*	-	[293]
Penicibrocazine B (**611**)	Marine-derived *Penicillium brocae* MA-231 from the mangrove plant *Avicennia marina*	Antimicrobial activity	[293]
Penicibrocazine C (**612**)	Marine-derived *Penicillium brocae* MA-231 from the mangrove plant *Avicennia marina*	Antimicrobial activity	[293]
Penicibrocazine D (**613**)	Marine-derived *Penicillium brocae* MA-231 from the mangrove plant *Avicennia marina*	Antimicrobial activity	[293]
Penicibrocazine E (**614**)	Marine-derived *Penicillium brocae* MA-231 from the mangrove plant *Avicennia marina*	Antimicrobial activity	[293]
Penicisulfuranol D (**615**)	Mangrove-derived *Penicillium janthinellum* HDN13-309 from the roots of *Sonneratia caseolaris*	-	[273]
Penicisulfuranol E (**616**)	Mangrove-derived *Penicillium janthinellum* HDN13-309 from the roots of *Sonneratia caseolaris*	-	[273]
Penicisulfuranol F (**617**)	Mangrove-derived *Penicillium janthinellum* HDN13-309 from the roots of *Sonneratia caseolaris*	-	[273]
Phomazine A (**618**)	Marine-derived *Phoma* sp. OUCMDZ-1847 from the mangrove plant *Kandelia candel*	-	[241]
Phomazine B (**619**)	Marine-derived *Phoma* sp. OUCMDZ-1847 from the mangrove plant *Kandelia candel*	Cytotoxic activity	[241]
	Marine-derived *Penicillium brocae* MA-231 from the mangrove plant *Avicennia marina*	Antimicrobial activity	[293]
Plectosphaeroic acid A (**620**)	Marine-derived *Plectosphaerella cucumerina*	Inhibition of indoleamine 2,3-dioxygenase	[254]
Plectosphaeroic acid B (**621**)	Marine-derived *Plectosphaerella cucumerina*	Inhibition of indoleamine 2,3-dioxygenase	[254]
Polanrazine B (**622**)	Plant pathogen *Phoma lingam*	Phytotoxic activity	[138]
Polanrazine C (**623**)	Plant pathogen *Phoma lingam*	Moderate and selective phytotoxicity by causing necrotic and chlorotic lesions	[138]
Polanrazine D (**624**)	Plant pathogen *Phoma lingam*	-	[138]
Pseudellone D (**625**)	Marine-derived *Pseudallescheria ellipsoidea* F42-3 associated with the soft coral *Lobophytum crassum*	-	[294]
Sch 54794 (**626**)	*Fusarium chlamydosporum* OUPS-N124 from the marine alga *Carpopeltis affinis*	Weak cytotoxic activity against P388 lymphocytic leukemia cells	[277]
Sch 54796 (**627**)	*Fusarium chlamydosporum* OUPS-N124 from the marine alga *Carpopeltis affinis*	-	[277]
Spirobrocazine A (**628**)	Mangrove-derived *Penicillium brocae* MA-231 from *Avicennia marina*	Moderate antibacterial activity	[205]
Spirobrocazine B (**629**)	Mangrove-derived *Penicillium brocae* MA-231 from *Avicennia marina*	-	[205]
Sporidesmin D (**630**)	*Pithomyces chartarum*	-	[295]
Sporidesmin F (**631**)	*Pithomyces chartarum*	-	[296]
T988 B (**632**)	*Tilachidium* sp.	Cytotoxic activity	[225]
	Antarctic psychrophilic fungus *Oidiodendron truncatum*	-	[211]

Note: MIC, minimum inhibitory concentration.
